# Genome-wide analysis of NGS data to compile cancer-specific panels of miRNA biomarkers

**DOI:** 10.1371/journal.pone.0200353

**Published:** 2018-07-26

**Authors:** Shib Sankar Bhowmick, Indrajit Saha, Debotosh Bhattacharjee, Loredana M. Genovese, Filippo Geraci

**Affiliations:** 1 Department of Computer Science and Engineering, Jadavpur University, Kolkata, India; 2 Department of Electronics & Communication Engineering, Heritage Institute of Technology, Kolkata, India; 3 Department of Computer Science and Engineering, National Institute of Technical Teachers’ Training & Research, Kolkata, India; 4 Institute for Informatics and telematics, National Research Council, Pisa, Italy; College of Bioinformatics Science and Technology, CHINA

## Abstract

MicroRNAs are small non-coding RNAs that influence gene expression by binding to the 3’ UTR of target mRNAs in order to repress protein synthesis. Soon after discovery, microRNA dysregulation has been associated to several pathologies. In particular, they have often been reported as differentially expressed in healthy and tumor samples. This fact suggested that microRNAs are likely to be good candidate biomarkers for cancer diagnosis and personalized medicine. With the advent of Next-Generation Sequencing (NGS), measuring the expression level of the whole miRNAome at once is now routine. Yet, the collaborative effort of sharing data opens to the possibility of population analyses. This context motivated us to perform an in-silico study to distill cancer-specific panels of microRNAs that can serve as biomarkers. We observed that the problem of finding biomarkers can be modeled as a two-class classification task where, given the miRNAomes of a population of healthy and cancerous samples, we want to find the subset of microRNAs that leads to the highest classification accuracy. We fulfill this task leveraging on a sensible combination of data mining tools. In particular, we used: differential evolution for candidate selection, component analysis to preserve the relationships among miRNAs, and SVM for sample classification. We identified 10 cancer-specific panels whose classification accuracy is always higher than 92%. These panels have a very little overlap suggesting that miRNAs are not only predictive of the onset of cancer, but can be used for classification purposes as well. We experimentally validated the contribution of each of the employed tools to the selection of discriminating miRNAs. Moreover, we tested the significance of each panel for the corresponding cancer type. In particular, enrichment analysis showed that the selected miRNAs are involved in oncogenesis pathways, while survival analysis proved that miRNAs can be used to evaluate cancer severity. Summarizing: results demonstrated that our method is able to produce cancer-specific panels that are promising candidates for a subsequent in vitro validation.

## Introduction

Timing and accuracy in cancer diagnosis are among the most critical factors that influence the clinical history of a patient. Until recently, the histological analysis of a small sample of tumor cells has been the only tool for cancer classification. The complexity of this pathology and the histological similarity of certain sub-classes, however, have motivated researchers to find easier diagnosis techniques that can also be used on a large scale [1].

MicroRNAs (miRNAs) are short non-coding RNAs, whose size is approximately ranged between 22 and 25bp, that influence the regulation of target genes by imperfect binding to complementary regions of messenger transcripts [2]. Being involved in several biological processes [3–5], modifications of the expression profile of this class of RNAs have been investigated in conjunction with cancer [5]. A further property that makes miRNAs an attractive target for research of non-invasive biomarkers is that they are released outside the cell and can be easily quantified in the serum [6] using RT-qPCR [7]. Moreover, the relationship between cellular and circulating miRNAs has already been elucidated [8]. These facts open to a new generation of miRNA-based non-invasive biomarkers [9].

Over the last decade, several studies on cancer have pointed out specific miRNAs as putative biomarkers for a variety of purposes (see reference [10] for a survey). Applications range from the classification of the tissue of origin [11] or the cancer type [12–14] to the personalized therapy [15, 16]. However, the discovery process has been complicated from the limited amount of data and from the fact that differential expression has been the only discriminating feature to drive researchers.

The drop of the cost of NGS technologies for whole miRNAome analysis and the availability of public repositories of miRNA profiles of cancerous samples (i.e. The Cancer Genome Atlas [17]), have raised the question whether the traditional pipeline in which the in-vitro exploratory activity is performed upstream the statistical analysis can be turned, making machine learning approaches to drive subsequent in-vitro experiments. Although few preliminary publications [18] foster a positive answer to this question, an agreed compendium of the miRNA profiles associated with all the cancer types is still far away.

In this work, we argue that panels based only on miRNAs differentially expressed between tumor and control samples might lack possible complex relationships among patterns of expression levels. Consequently, we propose to map the task of discovering putative miRNA biomarkers into the machine learning problem of selecting a restricted set of features that lead to the highest classification accuracy of a two-class classification task.

This problem formulation, however, raises the issue of building a pipeline that provides the highest classification accuracy. Since feature selection and classification are probably the two most studied problems in machine learning [19] [20] and in bioinformatics [21], [22] [23], exploring all the possible alternatives is impossible. We thus fixed some choices according to the general agreement in the literature and followed an explorative approach for other components.

Experiments on the 10 common cancer types from [24] showed that our proposed approach improves upon differential expression-based state-of-the-art methods not only in terms of accuracy, but also in terms of other relevant performance measures (i.e. FDR, sensitivity, specificity, etc.). The value of this result is twofold: firstly, it opens to a new methodological approach to differential expression analysis; moreover, it can be considered as a preliminary piece of evidence that relationships among the expression values of miRNAs might not be linear.

To conclude this work, we investigated the biological role of miRNAs in our panels. We found that most of them interact with the morphogenesis process and are involved in pathways which regulate cellular proliferation, growth, and survival.

## Materials and methods

The key idea behind our approach is that of mapping the problem of finding putative miRNA biomarkers into a two-class classification task. However, important differences between the two problems exist. In fact, increasing the accuracy of the canonical classification task can be achieved by providing new training examples; while in our setting the number of involved elements is bounded by the limited availability of samples. Another important difference is that, in our case, a high accuracy is not enough. In fact, we are interested in finding a restricted panel of miRNAs responsible for the correct classification. This last fact suggests that we can leverage on feature selection so as to maximize classification accuracy.

In this paper, we used an evolutionary optimization method called *Differential Evolution* (DE) [25–27] to explore the space of the subsets of miRNAs. The outcome of the classification performed via a *Support Vector Machine* (SVM) [28] is used to assess the quality of the selection while *Kernel Principal Component Analysis* (KPCA) [29] and *Principal Component Analysis* (PCA) [30] are used to keep unaltered the structural characteristics of the whole dataset during the selection process. In order to prevent the intrinsic randomness of DE causes the inclusion of irrelevant results in the returned panel, we have run the optimization algorithm 50 times and selected the final set of miRNAs by means of a finishing algorithm based on a majority voting scheme.

### Component analysis

Feature selection is a common tool to scale down a high-dimensional space. According with the purpose of the application, two main strategies can be chosen: dimensionality reduction and variable elimination (see [31] for an in-depth examination). Component analysis, as all the orthogonal transformations, belongs to the first category. The main advantage of this strategy is that the reduced space still maintains the structural properties of the original space. However, once a correlation in the reduced space is found, reverting to the original correlated dimensions is not possible. The *Differential Evolution* algorithm, instead, is a wrapper-based approach belonging to the class of variable elimination. Methods of this category iteratively select a subset of dimensions of the original space attempting to maximize a certain objective function (usually classification accuracy). Working with dimensions in the original space, the advantage of this approach is that of exactly knowing the variables under consideration. On the other hand, complex relationships among the features are not taken into account causing the removal of a dimension to brake possible unknown relationships.

In our framework, the need to identify the subset of miRNAs that maximize classification would force us to use variable elimination. However, this would mean giving up with the possibility of involving complex expression patterns in the process of selection of a panel and, in turn, reducing to a standard differential analysis. Combining the strengths of dimensionality reduction and variable elimination is hence needed. We achieved this goal by leveraging on the following two results. In [32] the authors proposed a dimensionality reduction scheme in which the dataset is randomly partitioned into *K* homogeneous groups and PCA is applied to each of them. The final dataset consists in a matrix in which the *i*-th column is the principal component of the corresponding partition. Experiments in [32] showed that this reduction strategy still preserves the structural characteristics of the original dataset, suggesting that for large-enough partitions, PCA obeys a sort of distributive law. In [33] the authors showed that a wrapper-based dimensionality reduction approach (i.e. a method that exploits the outcome of classification to select features) can be improved applying in cascade a filter-based method (i.e. an algorithm independent of the classification algorithm).

Let *E* be a *s* × *d* matrix with the expression levels of *d* miRNAs on *s* samples and let *E*(*v*) be a subset of *n* < *d* columns of *E* as specified by a *n*-dimensional vector *v*.

Building upon [32], we pre-processed *E* by applying component analysis and extracting the first *n* components (for a fixed value of *n*). The resulting matrix $\hat{E}$ represents the projection of *E* into a new smaller space with *s* rows and *n* columns. We observed that, when $\hat{E}$ maintains the same structural characteristics of the original expression matrix (i.e. saving most of its variance), it can be used as an encoder to compress a miRNA vector of *E* into a smaller one with a marginal loss of information. Consequently, multiplying each vector of *E*(*v*) (thus selecting *n* miRNAs) with the matrix $\hat{E}$ we obtain a new matrix $\hat{E}\left(v\right)$ that: keeps unaltered most of the structural characteristics of the original expression matrix, and highlights the contribution of the selected miRNAs.

Choosing a convenient component analysis algorithm able to preserve variance is complicated by the fact that different datasets can have profound differences that reflect on the outcome of the methods. PCA is fast and appropriate when data do not exhibit non-linear relationships, while KPCA is more able to preserve complex relationships. We empirically chose per dataset component analysis by testing the accuracy of classification either after PCA or KPCA and deciding for the most promising.

Following a philosophy similar to that of [33], we used the DE algorithm to select the miRNAs specified in the vector *v* and the matrix $\hat{E}\left(v\right)$ (in place of *E*(*v*)) as input for the classifier that computes the fitness function. As a consequence, the fitness value does no longer reflect the classification accuracy of a panel based on *E*(*v*). Nevertheless, $\hat{E}\left(v\right)$ is likely to be more effective than *E*(*v*) to quickly lead DE to an optimal solution.

### Differential evolution

Differential Evolution (DE) [25–27] is an evolutionary optimization tool aimed at finding a global optimum solution in a *n*-dimensional real parameter space $R^{n}$.

Like any other evolutionary algorithm, DE maintains a set *V* = {*v*[1], …, *v*[*I*]} of *I* candidate solutions (*I* = 50 in our experiments) and applies a series of operators to evolve to the next generation. Each solution, in turn, is a vector *v* storing *n* features (miRNAs in our case). For clarity, we often add a subscript in the notation to denote the generation.

Let *M* be the sorted static list of miRNAs involved in a given cancer dataset. Say |*M*| = *d*. A vector *v* ∈ *V* consists of *n* disjoint references (as index) to elements of the table *M*.

Since the choice of the length *n* of *v* is arbitrary, in our experiments we tested several possible assignments of it. We found that consistently the lower *n*, the better our method works. In particular, *n* = 10 has shown to be an optimal value for our purposes (see section “Parameter setting”).

**Fig 1:**
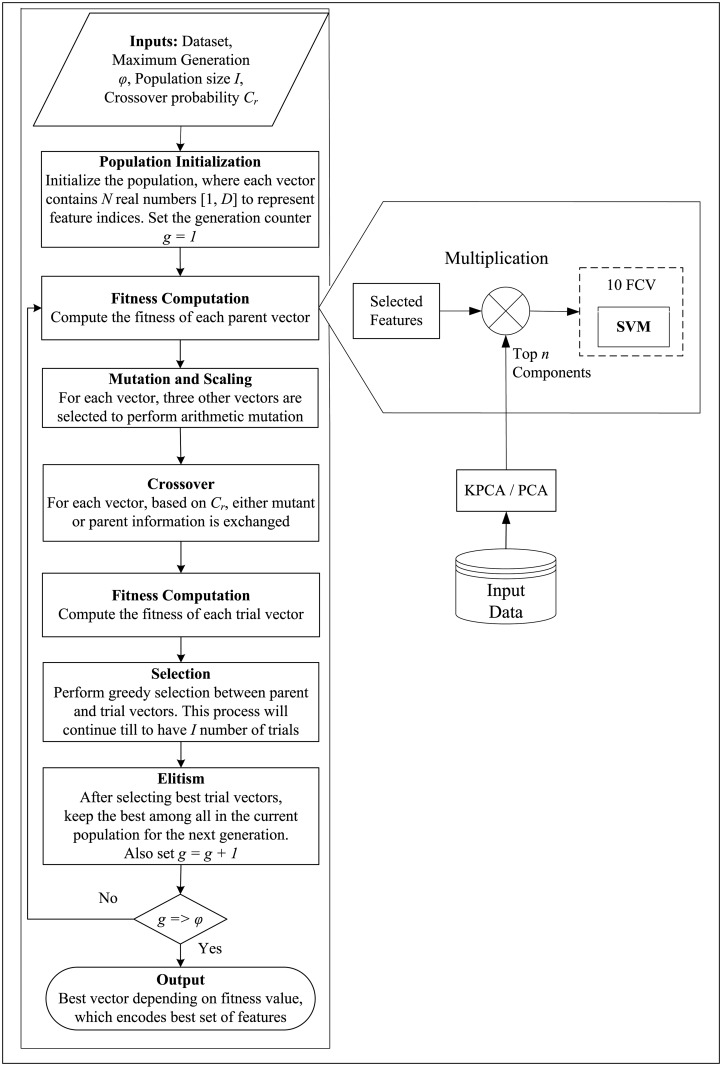
Graphical representation of the differential evolution based feature selection.

In order to initialize the first generation of candidate solutions, for each *v* ∈ *V*, DE selects *n* random elements from the list *M* of miRNAs. Subsequently, at the *i*-th generation (*i* ∈ [1, …, *φ*]), the mutation and scaling operators are used to evolve every individual vector *v*_*i*_[*j*], *j* ∈ [1, *I*] (called target) into a new vector *v*_*i*+1_[*j*] (called trail) and the operator of crossover is applied to hybridize a fraction *C*_*r*_ of the new population. We set *C*_*r*_ = 0.8 in our experiments. Finally, a fitness function is computed to compare each trail vector with its corresponding target. The vector with highest fitness is selected and promoted to the next generation, while the other is discarded. As a result, the population never deteriorates, giving either the same or a better fitness status. Elitism operator is used to prevent the DE algorithm to fall into a local maximum. To do this, the operator selects the solution with highest fitness value in the current population and promotes it to the next generation. The evolution process is repeated until a predefined stopping criterion is met (50 iterations in our experiments). A flow chart of DE based feature selection is shown in Fig 1.

#### Population initialization

The first generation of the population consists of a randomly initialized set of vectors *V*_0_ = {*v*_0_[1], …, *v*_0_[*I*]}. For each vector *v* we used a uniform random number generator to select *n* disjoint items from the table *M* of miRNAs.

#### Fitness computation

Designing the fitness function to evolve *V*_*i*_ into *V*_*i*+1_ is the key choice for evolutionary algorithm developers. In fact, the selection of the candidate solutions at each generation strictly depends on it. In our case, we were interested in a function that promotes a vector *v* if the corresponding miRNAs are responsible of the discrimination between control and tumor samples. As many wrapper-based approaches, we used the outcome of classification to compute fitness.

Following the intuition in [33], we combined the classification-based fitness evaluation with a method independent from classification. In particular, by multiplying the matrix *E*(*v*) times the matrix $\hat{E}$ derived from the component analysis, we obtained a new matrix $\hat{E}\left(v\right)$ that preserves the variability of the original dataset *E*, but enhances the contribution of the miRNAs in *v*. The matrix $\hat{E}\left(v\right)$ is subsequently used as input for a SVM endowed with RBF kernel and the average accuracy is returned as a value of fitness. Aimed at enforcing stability, we applied 10-fold cross-validation to classification.

Notice that, as a consequence of the use of $\hat{E}\left(v\right)$, the fitness value does no longer reflect the real classification accuracy of a panel based on *E*(*v*). Nevertheless, $\hat{E}\left(v\right)$ is more effective than *E*(*v*) to quickly lead DE to discover an optimal solution.

#### Mutation and scaling

After fitness computation the mutation operator is applied so as to evolve *V*_*i*_ to the next generation. Each vector *v* is hybridized with two other random vectors by means of a linear combination of a subset of features. In particular, for each *j* ∈ [1, *I*] let *a*, *b*, *c* be three random values in the same range so that *j* ≠ *a* ≠ *b* ≠ *c*:

$$\begin{array}{c} v_{i+1}\left[j\right]\left[k\right]=v_{i}\left[c\right]\left[k\right]+\left\lfloor F\left(v_{i}\left[a\right]\left[k\right]-v_{i}\left[b\right]\left[k\right]\right)\right\rfloor \;\forall k\in \left[1,n\right] \end{array}$$

where the mutation factor *F* is a constant value in the range [0, 2]. As an effect of mutation, it may happen that the value of some elements in *v*_*i*+1_ fall outside the range [1, *d*]. Hence, scaling was applied as follows:

$$\begin{array}{c} v_{i+1}\left[j\right]\left[k\right]=\{\begin{array}{cc} v_{i+1}\left[j\right]\left[k\right]+d & \;\text{if}\;v_{i+1}\left[j\right]\left[k\right]<1\\ v_{i+1}\left[j\right]\left[k\right]-d & \;\text{if}\;v_{i+1}\left[j\right]\left[k\right]>d\\ v_{i+1}\left[j\right]\left[k\right] & \;\text{otherwise}. \end{array} \end{array}$$

#### Crossover

Along the execution DE could enter in a loop that converges very slowly to the optimal fitness value causing the algorithm to waste a considerable amount of time performing iterations that little add to the final solution. Crossover is an operator introduced to break these loops switching a fraction of the elements of a vector *v*[*i*] with the corresponding elements of another vector *v*[*j*]. In our case, crossover was applied to restore a fraction of the elements from the target vector *v*_*i*_.

Let *C*_*r*_ ∈ [0, 1] be an user defined real constant, and *rand*_*k*_() be the *k*-th evaluation of a function that returns a random value within the same range; given *j* ∈ [1, *I*], the elements of the vector *v*_*i*+1_[*j*] are modified according to the following equations:

$$\begin{array}{c} v_{i+1}\left[j\right]\left[k\right]=\{\begin{array}{cc} v_{i+1}\left[j\right]\left[k\right] & \;\text{if}\;rand_{k}\left(\right)\leq C_{r}\\ v_{i}\left[j\right]\left[k\right] & \;\text{otherwise}\;. \end{array} \end{array}$$

#### Selection

As a result of mutation and crossover, a new generation of candidate solutions *V*_*i*+1_ is computed. However, the random processes involved in the evolution do not guarantee that all the elements of the next generation attain a fitness score higher than their targets. Retaining solutions with lower fitness can misdirect DE searching by triggering a cascade of evolutions in which the overall fitness reduces.

In order to avoid such a situation, the selection operator compares the fitness value of each *v*_*i*+1_[*j*] with the corresponding target *v*_*i*_[*j*] and evolves to the next generation the vector with higher fitness value. This ensures that the overall fitness of the population can never degenerate but remains stable in the worst case.

More formally: for *j* ∈ [1, *I*] the next generation is represented as follows:

$$\begin{array}{c} v_{i+1}\left[j\right]=\{\begin{array}{cc} v_{i+1}\left[j\right] & \;\text{if}\;fitness\left(v_{i+1}\left[j\right]\right)>fitness\left(v_{i}\left[j\right]\right)\\ v_{i}\left[j\right] & \;\text{if}\;fitness\left(v_{i+1}\left[j\right]\right)\leq fitness\left(v_{i}\left[j\right]\right). \end{array} \end{array}$$

#### Elitism

Although the selection operator ensures that the overall fitness of the next generation can never be lower than that of the previous one, it does not prevent convergence to a local maximum. In fact, it may happen that exist *j*, *k* ∈ [1, *I*], *j* ≠ *k* such that the highest fitness value between *v*_*i*_[*j*] and *v*_*i*+1_[*j*] is lower than the minimum fitness over the pair *v*_*i*_[*k*] and *v*_*i*+1_[*k*]. In this case, it would happen that the maximum over *j* would be evolved to the next generation even though it has fitness value lower than the minimum over *k*. The elitism operator is applied in order to mitigate this phenomenon.

Given the vectors: *v*_*i*_[*k*] with the highest fitness in generation *i* and *v*_*i*+1_[*j*] with the lowest fitness in generation *i* + 1, the elitism operator carries over the element that satisfies the following equation:

$$\begin{array}{c} argmax(fitness\left(v_{i}\left[k\right]\right),fitness\left(v_{i+1}\left[j\right]\right)) \end{array}$$

### Panel finishing

As all the evolutionary methods, DE needs to know in advance the number of elements *n* that will constitute the final solution and heavily relies on randomization to dwindle the probability of returning suboptimal solutions containing false positives and/or false negatives. The consequence of these facts, however, is that different runs of DE are likely to lead to slightly different panels each of which potentially containing a small fraction of uninteresting miRNAs.

Aimed at counteracting the effect of randomization so as to produce a stable and reliable panel in which the probability of false positives or false negatives is minimized, we run DE *θ* = 50 times, then we used a majority voting schema to select (a variable number of) miRNAs.

Let *R* = {*r*_1_, …, *r*_*k*_} be the list of miRNAs reported by any of the runs of DE and let #(*r*_*i*_) be the number of times it was reported. We maintained *R* sorted so that *i* < *j* ⇒ #(*r*_*i*_) ≥ #(*r*_*j*_). For increasing values of *i* ∈ [1, *k*], we run the classification (endowed with 10-fold cross-validation) using the subset of the first *i* elements of *R* and computed the accuracy (See S1 Table) As a final panel, the subset of *R* maximizing accuracy was chosen.

Although this procedure does not ensure the absence of false positives or false negatives, some considerations can be done. The presence of false positives is made unlikely by the sorting procedure and the adaptive computation of the number of elements in the panel. In fact, since a given false positive is supposed to be reported less frequently than all the true positive miRNAs, it would appear in the tail of *R* after sorting and would contribute to reduce the classification accuracy of a panel. Consequently, it is likely that it would be filtered out by the selection algorithm. The presence of false negatives, instead, is related to the choice of the number of runs and the corresponding expected number *k* of miRNAs belonging to *R*. If the selection of miRNAs were a random process, the expected value of *k* after 50 runs would be 399 with an expected increase of 6 new miRNAs in the subsequent run (see next section for an in-depth explanation of these estimations). S1 Table instead shows that *k* fell in a range between 11 and 17 in our experiments. This difference suggests that running DE 50 times is enough to argue that all the true positives were included in *R* and thus false negatives are unlikely.

#### Expected number of miRNAs in a panel

We provide here a succinct mathematical justification of the chosen number of independent runs of DE as well as we show that the final selection of miRNAs is not the result of a random process. Let us suppose that the set of *n* miRNAs that DE selects at each run is random. According to our experimental setting we have the following parameters: *n* = 10 is the number of selected elements in an independent run of DE, *d* = 1046 is the number of miRNAs measured in a sample in TGCA, *θ* = 50 is the number of runs.

For *i* = 1, …, *d*, let *X*_*i*_ be a Bernouilli distributed indicator variable where *X*_*i*_ = 1 if the miRNA *m*_*i*_ never shows up. The probability that *m*_*i*_ is selected in one run is *n*/*d*, thus the probability that it is never selected is 1 − *n*/*d*. Since each of the *θ* runs is independent, we have that

$$\begin{array}{c} E\left[X_{i}\right]=Pr\left(X_{i}=1\right)={\left(1-\frac{n}{d}\right)}^{\theta } \end{array}$$

Let $X=\sum_{i=1}^{d}X_{i}$ be the random variable that counts the number of miRNAs that does not belong to the final set *R* of the miRNAs reported at least once. By linearity of the expectation we have:

$$\begin{array}{c} E\left[X\right]=\sum_{i=1}^{d}E\left[X_{i}\right]=d*{\left(1-\frac{n}{d}\right)}^{\theta } \end{array}$$

In turn, the number *k* of expected miRNAs reported at least once is:

$$\begin{array}{c} E[(d-X)]=d-E[X] \end{array}$$

Substituting the parameters with our values we obtain that:

1. the expected number of miRNAs reported at least once after *θ* = 50 independent executions of the random algorithm is 398.96 and,
2. the number of new miRNAs added in a further run would be 6.18.

As shown in S1 Table, after 50 independent runs the expected number of miRNAs selected using a random algorithm is already at least one order of magnitude larger than that reported by our method. This result proves that the panel selection procedure is not affected by the randomization in DE.

## Results and discussion

### Dataset

For our experiments we collected the NGS-based miRNA expression data from The Cancer Genome Atlas. Our desire of producing a specific panel for the most comprehensive possible list of cancer types would have motivated us to run our method on all the available datasets. However, as all the machine learning approaches, our method is avid of examples to ensure a highly accurate result. Thus, we focused only on the subset of the 10 well-represented and common cancer types suggested in [24].

For each considered type, The Cancer Genome Atlas provides the expression levels (after removal of batch effects) of 1046 miRNAs belonging to a variable number of samples unevenly partitioned between patients (see Table 1 for details) and controls. For our convenience, we further normalized the expression values converting the reads per million count into a *log*_2_ scale. In order to eliminate possible overfitting effects due to the unbalanced distribution between the two classes we had to group together controls regardless the tissue type obtaining a total of 412 samples. This could potentially cause inconsistencies when the controls of the tissue under consideration have an expression profile consistently different from the rest of the controls. In particular, it may happen that the differential expression between the tumor samples and the control samples depends on the expression level of samples of other tissue types. This could potentially cause the introduction in the panel of spurious miRNAs (false positives) not really predictive of the cancer on the tissue under consideration. Aimed at quantifying the extent of this phenomenon, we run NOISeqBIO [34] in order to perform the differential analysis of miRNAs between samples of a specific tissue and samples of all the other tissues. Experiments setting p-value < 0.01, fold change > 2 and average RPM > 100 as thresholds, showed that on average only the 7.26% of miRNAs can be considered significantly differentially expressed.

**Table 1:** Type and number of tumor samples for the cancer types involved in our study. Each cancer type is coupled with 412 control samples to form a two-class dataset.

Cancer	Description	Tumor Samples
BRCA	Breast invasive carcinoma	762
KIRC	Kidney renal clear cell carcinoma	255
LGG	Brain lower grade Glioma	526
LIHC	Liver hepatocellular carcinoma	374
LUAD	Lung adenocarcinoma	452
PAAD	Pancreatic adenocarcinoma	179
PRAD	Prostate adenocarcinoma	495
SKCM	Skin cutaneous melanoma	450
STAD	Stomach adenocarcinoma	395
THCA	Thyroid carcinoma	510
**Total**		**4398**

### Evaluation

The main achievement of our work is that of distilling, for each investigated cancer type, the specialized panel of miRNAs shown in Table 2. Performing diagnoses, these panels can be used in conjunction with the corresponding trained SVM classifier to enforce the evidence of the presence/absence of cancer. However, as for all the machine learning-based techniques, our results depend on a series of individual choices whose impact needs to be elucidated. Since exploring even a small portion of the universe of alternatives is impractical, in the following sections we provide for each choice an experimental evaluation of its contribution. In particular we are going to discuss the parameter setting of the machine learning algorithms, compare several feature selection methods, contrast clustering with classification and compare our solution with standard differential expression analysis.

**Table 2:** miRNA panels for the investigated cancer types and their FDR corrected *p*-values.

Cancer	Rank	miRNA	FDR	Cancer	Rank	miRNA	FDR
BRCA	1	hsa-mir-140	3.83e-15	KIRC	1	hsa-mir-542	5.17e-15
	2	hsa-mir-100	4.35e-05		2	hsa-mir-3065	1.64e-14
	3	hsa-mir-375	1.19e-09		3	hsa-mir-361	4.63e-16
	4	hsa-mir-328	1.37e-14		4	hsa-mir-374a	1.58e-09
	5	hsa-mir-744	1.24e-15		5	hsa-mir-500a	1.52e-08
	6	hsa-mir-324	1.19e-09		6	hsa-mir-103-2	1.49e-02
	7	hsa-mir-30e	3.32e-11		7	hsa-mir-18a	9.45e-14
	8	hsa-mir-1307	3.17e-12		8	hsa-mir-203	1.49e-02
	9	hsa-mir-26a-2	1.48e-10		9	hsa-mir-576	1.09e-12
					10	hsa-mir-29c	9.45e-14
LGG	1	hsa-mir-335	9.99e-01	LIHC	1	hsa-mir-10b	1.17e-15
	2	hsa-mir-148b	2.52e-15		2	hsa-mir-92a-2	1.26e-08
	3	hsa-mir-21	9.01e-16		3	hsa-mir-20a	6.58e-11
	4	hsa-mir-155	1.19e-11		4	hsa-mir-181a-1	5.09e-14
	5	hsa-mir-574	5.89e-13		5	hsa-mir-17	1.08e-10
	6	hsa-let-7e	1.44e-13		6	hsa-mir-92a-1	5.29e-16
	7	hsa-mir-455	1.82e-16		7	hsa-mir-455	3.03e-14
	8	hsa-mir-128-1	1.09e-12		8	hsa-mir-34a	7.51e-10
	9	hsa-mir-424	2.24e-14				
LUAD	1	hsa-mir-140	6.47e-11	PAAD	1	hsa-mir-582	1.91e-14
	2	hsa-mir-103-1	2.76e-09		2	hsa-mir-151	1.09e-14
	3	hsa-mir-195	8.12e-12		3	hsa-mir-130b	1.33e-15
	4	hsa-mir-874	2.82e-15		4	hsa-mir-194-2	4.01e-12
	5	hsa-mir-149	3.00e-13		5	hsa-mir-409	1.09e-14
	6	hsa-mir-629	4.79e-16		6	hsa-mir-181a-1	3.67e-14
	7	hsa-mir-141	2.51e-12		7	hsa-mir-598	2.08e-12
	8	hsa-mir-574	1.19e-09		8	hsa-mir-424	1.20e-13
	9	hsa-let-7i	2.86e-08		9	hsa-mir-574	4.63e-16
	10	hsa-mir-625	1.24e-15				
PRAD	1	hsa-mir-27b	6.47e-11	SKCM	1	hsa-mir-27b	4.66e-12
	2	hsa-mir-1287	9.49e-10		2	hsa-mir-30b	5.47e-14
	3	hsa-mir-27a	4.67e-10		3	hsa-mir-425	3.49e-08
	4	hsa-mir-342	3.93e-14		4	hsa-mir-30a	2.85e-10
	5	hsa-mir-146b	1.01e-13		5	hsa-mir-155	7.02e-16
	6	hsa-mir-3065	2.34e-10		6	hsa-let-7g	5.75e-11
	7	hsa-mir-128-2	7.55e-12		7	hsa-mir-576	2.14e-11
	8	hsa-mir-625	9.45e-14		8	hsa-mir-99b	5.75e-11
STAD	1	hsa-mir-134	1.95e-14	THCA	1	hsa-mir-660	3.92e-11
	2	hsa-mir-381	3.52e-14		2	hsa-mir-20a	7.16e-11
	3	hsa-mir-142	1.29e-12		3	hsa-mir-15a	2.90e-09
	4	hsa-mir-337	1.82e-13		4	hsa-mir-93	1.70e-09
	5	hsa-mir-127	1.20e-07		5	hsa-mir-576	6.48e-09
	6	hsa-mir-409	5.64e-08		6	hsa-let-7a-2	1.86e-13
	7	hsa-mir-542	9.79e-10		7	hsa-let-7a-1	1.45e-11
	8	hsa-mir-744	5.34e-10		8	hsa-mir-24-2	2.36e-15
	9	hsa-mir-128-1	2.28e-11		9	hsa-mir-33a	9.99e-13
	10	hsa-mir-199b	3.20e-06				
	11	hsa-mir-379	2.14e-07				

#### Parameter setting

Parameter setting is a thorny but unavoidable task for all machine learning tools. Some parameters can be chosen following best practices from the literature, others require a problem specific experimental evaluation. We could set the parameters of DE and SVM in accordance with [27] while we experimentally investigated the parameter *n* that controls the number of components in the feature selection. This is because *n* is a problem specific parameter that should be of the same order of magnitude of the expected number of miRNAs in a panel. In particular, for DE we set: the initial population *I* = 50, the number of generations *φ* = 50, the mutation factor *F* = 1, and the crossover probability *C*_*r*_ = 0.8. The RBF (Radial Basis Function) kernel used by SVM is controlled by means of two parameters: *γ*, and the trade-off between training error and margin *C*. We set *γ* = 0.5 and *C* = 2.0.

In absence of a hint about a sensible expected number of miRNAs in a panel, we investigated several assignments of *n* for each of which we run 50 instances of KPCA+SVM and PCA+SVM, then we evaluated the corresponding average classification accuracy. As Table 3 shows, underestimating *n* (see column *n* = 5) could cause a relevant miRNA not to appear in a panel reducing accuracy. For higher values, instead, we observed that increasing the number of features the noise overwhelms the signal and thus the accuracy drops. As a result we set *n* = 10. Interestingly, although we could not find a biological justification, this value is consistent with other independent results where miRNA-based panels contain from 7 up to 10 elements (see: [35], [36], [37]).

**Table 3:** Average SVM classification accuracy of kernel and non-kernel based methods varying the number of selected miRNAs in the feature selection step.

Cancer	KPCA				PCA			
	n = 5	n = 10	n = 20	n = 30	n = 5	n = 10	n = 20	n = 30
BRCA	90.87	**91.74**	89.16	56.07	91.18	**92.05**	89.53	60.42
KIRC	91.63	**92.81**	90.87	88.37	91.21	**92.26**	91.33	69.39
LGG	90.87	**92.93**	91.00	89.89	90.75	**92.63**	91.15	90.19
LIHC	91.30	**92.83**	91.75	88.58	91.35	**92.92**	90.16	89.75
LUAD	90.33	**92.92**	92.14	90.34	89.63	**92.76**	90.73	88.30
PAAD	91.77	**93.00**	91.21	75.17	90.34	**92.60**	75.37	75.17
PRAD	90.60	**92.85**	90.58	89.06	90.05	**92.55**	90.05	67.39
SKCM	92.28	**93.00**	91.75	90.33	91.21	**92.92**	91.90	89.63
STAD	90.63	**92.67**	91.11	91.11	91.56	**92.84**	90.29	80.44
THCA	90.67	**92.78**	91.95	87.76	89.94	**92.70**	90.90	78.62
Average	91.10	**92.75**	91.15	84.67	90.72	**92.62**	89.14	78.93

#### Evaluation of feature selection

Assessing the miRNA selection process, we compared the classification accuracy of a SVM pipelined either with the panels obtained using our approach or with other feature selection methods. We used four basic classification measures (namely: Accuracy, F-measure, Matthews correlation coefficient (MCC) and Area under the curve (AUC)). For ease of comparison these quality measures have been consolidated summing them to form a single scalar Aggregated Score (AS). A similar combination of indices has previously been used in [38], even though we used a different pool of measures. The Aggregated Score is bounded in the range [0, 4] and a higher value corresponds to a more accurate result.

As for the other methods, the vastity of the literature on feature selection makes a comprehensive comparison impractical. Thus, we narrowed to the most popular algorithms covering the two main feature selection approaches: component analysis and statistical methods. In addition, we included Random Forests (RFFS) as a representative of feature selection algorithms based on machine learning [39].

We included in the group of component analysis methods PCA and KPCA, either with or without the DE algorithm, so as to enable the evaluation of the individual contribution of each of our choices. Of the same category we included: Kernel Entropy Component Analysis (KECA) [40], and Independent Component Analysis (ICA) [41].

As for statistical approaches we included: Signal-to-Noise Ratio (SNR) [42], Welch’s t-test [43], Wilcoxon ranksum test [44], Joint Mutual Information (JMI) [45], minimum Redundancy Maximum Relevance (mRMR) [46], Mutual Information Feature Selection (MIFS) [47].

In order to ensure homogeneous experimental conditions, we fixed the number *n* of returned features to 10 for all the compared feature selection methods. In addition, we used 10-fold cross-validation so as to prevent biases due to the classification process.

**Fig 2:**
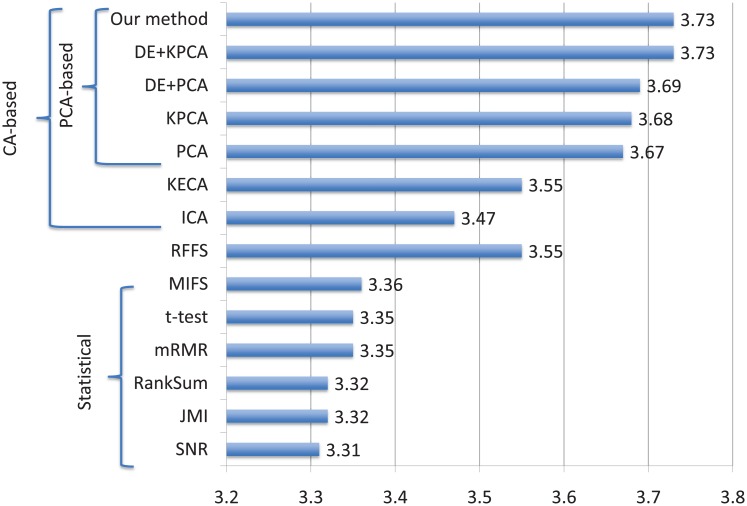
Average aggregated score of the compared algorithms. This score is the mean of all the per cancer type ASs and it is bounded in the range [0, 4]. We narrowed the x-axis in a shorter range so as to highlight the differences among the methods.

Table 4 reports for each pair cancer type/method the aggregated score, Fig 2 summarizes the same data averaging them by method, while details are left to S1 Appendix).

**Table 4:** Aggregated score derived from: Accuracy, F-measure, Matthews correlation coefficient (MCC) and Area under the curve (AUC) for SVM classification after different feature selection methods. AS is bounded in the range of [0, 4].

Algorithm	BRCA	KIRC	LGG	LIHC	LUAD	PAAD	PRAD	SKCM	STAD	THCA
Our method	3.75	3.80	3.85	3.69	3.75	3.74	3.69	3.80	3.61	3.66
DE+KPCA	3.69	3.72	3.77	3.68	3.76	3.76	3.75	3.78	3.71	3.73
DE+PCA	3.70	3.67	3.68	3.74	3.69	3.67	3.69	3.71	3.73	3.69
KPCA	3.67	3.67	3.69	3.67	3.68	3.71	3.68	3.71	3.67	3.68
PCA	3.67	3.66	3.68	3.69	3.68	3.68	3.68	3.68	3.68	3.68
KECA	2.57	3.59	3.65	3.68	3.69	3.65	3.68	3.69	3.68	3.65
ICA	2.79	3.66	3.50	3.53	3.52	3.65	3.53	3.54	3.52	3.47
RFFS	3,58	3,57	3,65	3,54	3,59	3,45	3,49	3,61	3,49	3,58
MIFS	3.10	3.69	3.40	3.35	3.43	3.17	3.29	3.53	3.33	3.38
t-test	3.11	3.35	3.37	3.39	3.39	3.35	3.40	3.49	3.32	3.38
mRMR	3.02	3.69	3.39	3.32	3.37	3.29	3.37	3.50	3.28	3.33
RankSum	3.07	3.27	3.33	3.34	3.35	3.31	3.40	3.53	3.33	3.32
JMI	3.02	3.42	3.39	3.34	3.37	3.27	3.31	3.47	3.32	3.33
SNR	3.07	3.27	3.33	3.34	3.35	3.31	3.31	3.49	3.32	3.32

Tests showed that all the seven component-analysis-based methods consistently achieve better results than statistical methods. In particular, according to Fig 2, the increment in terms of Average Aggregated Score of these methods is never lower than 0.11 (delta between ICA and MIFS). Narrowing to the 5 methods that use the principal component as a feature selection mechanism, the gap with the statistical methods further increases (+0.31 in the worst case). According to the detailed results reported in Table 4, we observed that this gap is independent from the cancer type. Moreover PCA-based methods tend to provide a more stable classification performance. This is particularly evident in the case of the BRCA, where statistical methods show a consistent drop of performance.

Random Forests induce a more stable and accurate classification than statistical methods achieving the same average evaluation of KECA, while they perform worse than the five PCA-based methods with a gap of at least 0.12 in terms of aggregated score.

Although, as shown in Table 4, PCA-based methods have an average performance better than all the other methods, the intrinsic randomness of the component analysis makes them to select substantially different sets of miRNAs at each run. Repeating the training and testing procedure until reaching a maximum (namely finding the set of components leading to the highest possible accuracy) is unfeasible because this would require testing all the possible combinations of miRNAs. The differential evolution overcomes this problem by guaranteeing to select a set of miRNAs that induce the classification accuracy to reach a maximum. This, in turn, limits the randomness of the component analysis. In fact, separate executions of the PCA-based methods in conjunction with the DE algorithm tend to return heavily overlapping sets of miRNAs. In particular, our experiments show that running DE 50 times, the set of miRNAs returned at least once contains only 17 elements in the worst case (see S1 Table for details). Moreover, as Fig 2 shows, DE caused also a generalized further increase of performance (even if with some exceptions).

Comparing our approach with other DE-based algorithms (without the finishing phase), we still observe a modest generalized performance increase over DE+PCA and a substantial equivalence with DE+KPCA. In this case, however, the advantage of our method is that of being deterministic and thus performance does no longer depend on the particular run.

#### Running time

Although the computational cost of compiling panels of biomarkers is not a crucial feature, it is nevertheless important to evaluate whether the underlying algorithms can be applied to large datasets or not using a standard workstation. While a single run of feature selection and classification is very fast, DE-based algorithms, which can involve several thousand invocations of the SVM, can be quite slow. In fact, according to our experimental parameters, each run of DE evolves 50 individuals for 50 generations. Every time the fitness function, and thus a run of SVM, needs to be computed. Moreover, our finishing procedure requires 50 independent runs of DE for a total of 125 thousand invocations of the SVM. Notwithstanding, both: the independence among candidate solutions within the same generation and the independence of different instances of DE, open to the possibility of attaining a consistent performance speedup by parallelizing the computation of the fitness functions.

We measured the running time of all the tested methods on a Intel^™^ Core-I7 workstation endowed with 6Gb of RAM, Microsoft Windows^™^ 64bit and Matlab^™^ R2014a. According to our experiments (see Table 5 and S1 Appendix), all the statistical methods (except t-test) took less than one second to finish, random forests required 8 seconds, component analysis-based methods completed in less than 10 seconds and the t-test needed about one minute. On the basis of the estimated number of calls to the SVM, one would expect that DE-based methods would spend several orders of magnitude more time than the other methods. Table 5, instead, shows that DE+PCA and DE+KPCA accomplish their task in the affordable time of respectively 12 and 14 minutes. This lower time not only depends on parallelism, but also on the fact that SVM is much faster than component analysis (see running time of KPCA and PCA in Table 5 for an estimation) and takes on average only 0.497 seconds. As a result, our choice of using the component matrix derived from the entire dataset instead of computing a new one at every invocation of the fitness function has the effect of mitigating the overall cost of DE-based methods, and, thus, keeping the computation running in an affordable time.

**Table 5:** Running time of the compared methods (Format: hh:mm:ss.cents).

Algorithm	Average time
Our method	03:02:43.64
DE+KPCA	14:48.51
DE+PCA	11:57.29
KPCA	7.71
PCA	5.38
KECA	9.01
ICA	8.14
RFFS	7.86
MIFS	0.94
t-test	1:04.26
mRMR	0.52
RankSum	0.97
JMI	0.56
SNR	0.57

Requiring *θ* = 50 independent executions of a DE-based algorithm as well as the finishing phase, our method would be expected to require a long time to complete (as long as more than 12 hours). Instead, the independence of the instances of DE, as opposed to a certain degree of dependence within an instance of DE, ensures a full exploitation of the intrinsic parallelism of modern CPUs. Leveraging on this independence, a parallel implementation our method took on average only three hours to complete. This running time proves that our method can run on a standard architecture without the need to tradeoff the desired overall running time, and the value of *θ* (and the consequent probability of introducing false negatives).

#### Evaluation of classification vs clustering

We further investigated the relationships among the expression level of the miRNAs belonging to our panels. According to the general experience with biomarkers, one would expect that all the profiles belonging to a given category would be similar to each other, while profiles of different classes would be different. Under this hypothesis, distinguishing between tumor and control samples would reduce to a two-partitions clustering. On the other hand, miRNA profiles are known to be highly variable even among cells of the same type (see [48] for a recent survey), thus non-linear correlation among the expression level of the miRNAs is expected.

For each investigated cancer, we tested these hypotheses by bi-partitioning the samples according to their type, then measuring the homogeneity and separation of the profiles induced by the corresponding panel. As a definition for homogeneity and separation we used that of [49] endowed with the cosine similarity. According to this definition, a high homogeneity and low separation (note we use similarity and not distance) correspond to a good clustering.

Results reported in Table 6 confirmed the hypothesis that the relationship among the expression level of the miRNAs within a class is in general non-linear. In fact, with the only exception of SKCM, the homogeneity is always slightly higher than the separation indicating that the average intra-cluster distance is comparable with the extra-cluster distance. Consequently, these results can be interpreted as an empirical justification of our choice of using a feature selection method able to identify non-linear correlations as well as that of addressing the identification of putative miRNA biomarkers as a classification problem instead of a clustering one.

**Table 6:** Homogeneity and separation of the tumor and control samples.

Cell type	Homogeneity		Separation
	Tumor	Control	
BRCA	0.461	0.476	0.361
KIRC	0.597	0.548	0.449
LGG	0.777	0.928	0.801
LIHC	0.522	0.579	0.397
LUAD	0.899	0.886	0.869
PAAD	0.581	0.428	0.343
PRAD	0.814	0.545	0.623
SKCM	0.680	0.747	0.288
STAD	0.551	0.506	0.467
THCA	0.863	0.889	0.872
Average	0.674	0.6532	0,547

#### Comparison with differential expression analysis

Differential expression is the most commonly employed feature to discriminate two populations of samples. The assumption at the basis of this tool is that differences of the phenotype depend on the inversion of the expression level of each of the tested set of miRNAs. Although a certain degree of interdependence among expression levels has already been proved, until now it limits to direct or inverse pairwise proportionality. In this section, we report on a comparison of our method with a panel selection strategy based only on differentially expressed miRNAs. Experiments showed that narrowing only on this strategy can be limiting. In fact, in order to recover a classification quality of the same order of magnitude of our method, it might be necessary to involve tens of miRNAs in the evaluation of differential expression.

**Fig 3:**
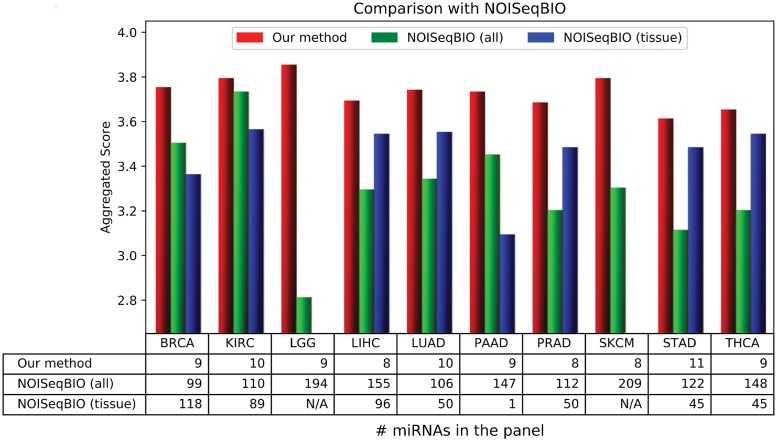
Comparison with panels derived from differential analysis.

Fig 3 shows the aggregated score achieved with our method and with NOISeqBIO [34] either using all the available samples or the tissue specific ones as controls. We set p-value < 0.01, fold change > 2 and average RPM > 100 as conservative thresholds to consider a miRNA as differentially expressed. The table in Fig 3 reports the number of differentially expressed miRNAs (for the reader convenience the size of our panels is reported as well).

According to the results shown in Fig 3, panels derived with our method are always more accurate than those obtained using NOISeqBIO to discriminate between tumor and control samples even using a small fraction of miRNAs. This confirms the limits of differential expression as the only criterion to compile a panel.

Narrowing only to the results obtained with NOISeqBIO, we observed that the larger variability due to the mix of control samples caused an increment of about two times of the number of miRNAs. Although a similar phenomenon could have happened also running our DE algorithm, we expect the final miRNA selection procedure to mitigate it. Focusing on the quality assessment, instead, the direct comparison of NOISeqBIO using all the samples or only the tissue specific ones revealed that the blowup of aggregated score due to the mix is as negligible as 0.14 on average (measured only on the 8 cancer types for which both methods provided a non-empty list of differentially expressed miRNAs).

### Biological significance

Gene set enrichment analysis (GSEA) [50] is a common tool to investigate a particular set of genes with the purpose of elucidating whether they can potentially have a role in a biological process. According to this idea, in this section we briefly report the outcome of our investigation about the biological functions associated with our panels. In particular, we used KEGG pathway enrichment analysis to uncover which pathways the selected miRNAs interact with, and Gene Ontology (GO) analysis in order to uncover the biological processes and molecular functions associated with our panels. In addition, we used the Kaplan-Meier (KM) survival analysis to test whether some of the miRNAs in our panels are suitable to predict the survival probability of the patient.

Both KEGG pathway enrichment analysis and Gene Ontology analysis rely on lists of genes and cannot be directly applied to miRNAs. In order to overcome this limitation, several studies have suggested that miRNA functions can be inferred from their corresponding mRNA targets [51]. Identifying miRNA-target interactions is a thorny task [52], thus several tools for target prediction have been proposed. In our case, we used miRSystem ver. 20160502 [53] that, among the others, has the advantage of integrating 7 established prediction tools.

#### Pathway analysis

In order to perform the KEGG pathway analysis we used Enrichr [54]. This tool computes the overlap between known KEGG pathways and Protein-Protein-Interaction (PPI) networks for the input set of genes. Subsequently each pathway is scored with a *p*-value so that the lower *p*-values the higher the probability of the pathway to be enriched with the set of genes. We report in Table 7 the most significant pathways for each investigated cancer type (See S2 Table for further details).

**Table 7:** Most common KEGG pathways associated with target genes of our cancer panels.

KEGG Pathway	BRCA	KIRC	LGG	LIHC	LUAD	PAAD	PRAD	SKCM	STAD	THCA
hsa04144: Endocytosis	✓			✓						✓
hsa04360: Axon guidance	✓			✓			✓		✓	
hsa05200: Pathways in cancer	✓	✓	✓	✓	✓	✓	✓	✓	✓	✓
hsa05205: Proteoglycans in cancer		✓	✓	✓	✓	✓	✓		✓	✓
hsa04151: PI3K-Akt signaling pathway		✓	✓		✓			✓		
hsa04010: MAPK signaling pathway			✓		✓		✓	✓		✓
hsa04550: Signaling pathways regulating pluripotency of stem cells			✓			✓			✓	

According to Table 7 we found that for all the investigated cancer types, miRNAs belonging to our panels enrich the pathway *hsa05200 Pathways in cancer*, while the pathway *hsa05205: Proteoglycans in cancer* is connected to 8 out of 10 cancer types. Other commonly appearing pathways are: *hsa04151: PI3K-Akt signaling pathway* and *hsa04010: MAPK signaling pathway* which regulate cellular proliferation, growth, and survival. In particular, *hsa04151: PI3K-Akt signaling pathway* is activated in response to many types of stimuli or toxic insults.

#### Gene Ontology analysis

Following a protocol similar to that we used for pathway analysis, we queried Enrichr [54] to derive the GO terms associated with our miRNA panels. Results reported in Table 8 show an intense activity in the nucleus where *GO:0005654 nucleoplasm* and *GO:0000785 chromatin* are the most stressed cellular components (see S3 Table for further details). This activity is more likely due to the DNA damage response [55]. Another highly represented category is *GO:0005911 cell-cell junction* which is involved in the connection between two or more cells.

**Table 8:** Most significant Gene Ontology terms associated with the miRNA targeted genes for Cellular Component obtained through enrichment analysis via Enrichr [54].

GO Cellular component	BRCA	KIRC	LGG	LIHC	LUAD	PAAD	PRAD	SKCM	STAD	THCA
GO:0005829 Cytosol	✓		✓	✓	✓	✓		✓		✓
GO:0044456 Synapse part				✓	✓	✓	✓		✓	✓
GO:0005654 Nucleoplasm	✓		✓	✓				✓	✓	✓
GO:0005911 Cell-cell junction	✓	✓					✓	✓		✓
GO:0045202 Synapse				✓	✓		✓			
GO:0000785 Chromatin	✓		✓					✓		

Morphogenesis, either at cellular level (*GO:0032989 cellular component morphogenesis*) or organ/tissue level (*GO:0009887 organ morphogenesis*, *GO:0048729 tissue morphogenesis*) is the most activated biological process (see Table 9 and S4 Table) and all the investigated cancer types cope with it. This is not surprising since morphogenesis is the process in which cells or anatomical structures are generated and organized. Notably in the case of Lung adenocarcinoma the morphogenesis process is more focused on the epithelium (*GO:0002009 morphogenesis of an epithelium*).

**Table 9:** Most significant Gene Ontology terms associated with the miRNA targeted genes for Biological Process obtained through enrichment analysis via Enrichr [54].

GO Biological process	BRCA	KIRC	LGG	LIHC	LUAD	PAAD	PRAD	SKCM	STAD	THCA
GO:0007411 Axon guidance	✓	✓		✓	✓		✓	✓	✓	✓
GO:0097485 Neuron projection guidance	✓	✓		✓	✓		✓	✓	✓	✓
GO:0045664 Regulation of neuron differentiation	✓			✓		✓	✓	✓		✓
GO:0032989 Cellular component morphogenesis					✓			✓	✓	✓
GO:0048598 Embryonic morphogenesis			✓		✓			✓	✓	
GO:0048729 Tissue morphogenesis			✓		✓	✓				

#### Kaplan-Meier survival analysis

Although it is a secondary feature, it is desirable for a biomarker to be able to estimate disease severity. Narrowing only to survival analysis, to be predictive the measure of the biomarker should be proportional (or inversely correlated) to survival time. In our case, however, the non-linear relationship among the expression values of the miRNAs, makes the use of a panel as a whole not feasible. Thus, we tested individually every miRNA to check whether there exists in each panel at least one element whose expression value can directly be correlated with survival time.

We used the median value to divide the population of patients in two balanced groups either with high or low expression level. Then we used the time to death from the TGCA clinical data to derive the Kaplan-Meier (KM) survival curves. Finally, we performed the log-rank test to check whether the two groups have different survival probability or not.

**Fig 4:**
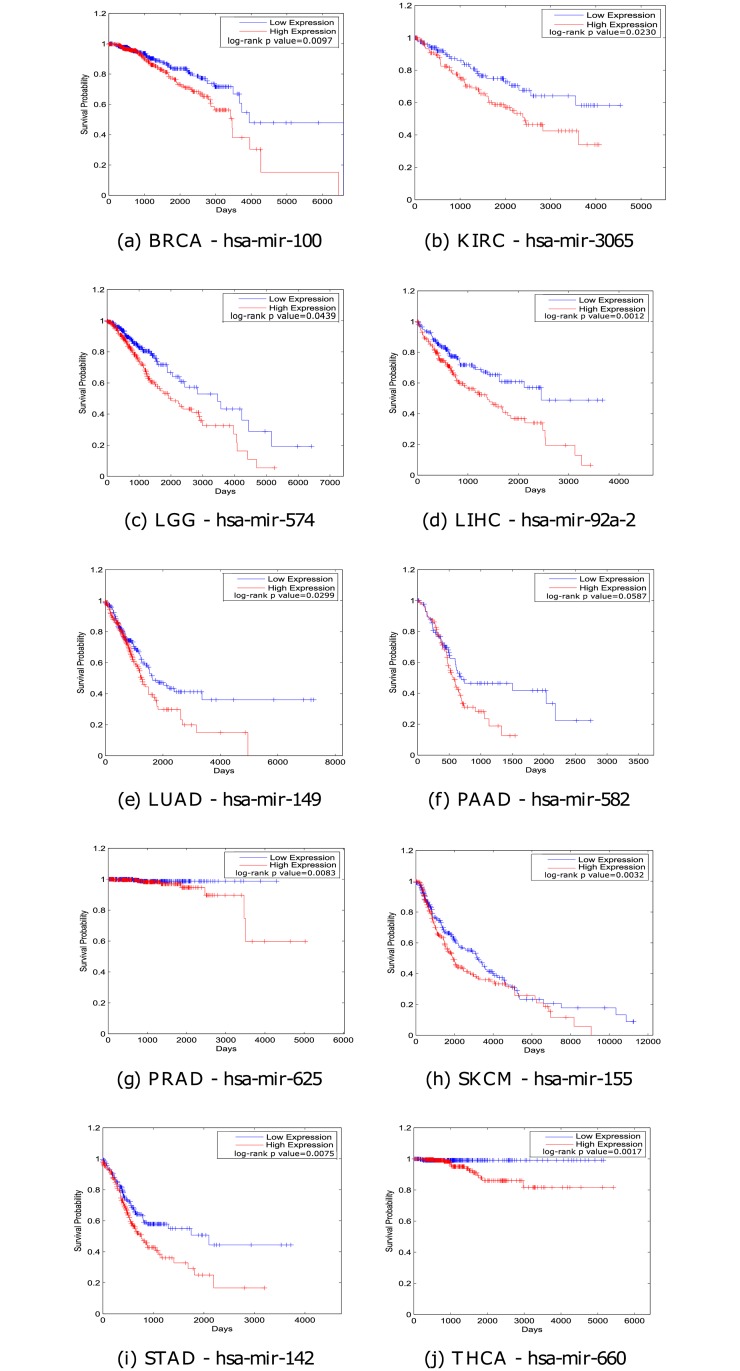
Kaplan-Meier survival plots of *10* best miRNAs based on lowest log-rank *p*-values for the respectively cancer types, (a) BRCA, (b) KIRC, (c) LGG, (d) LIHC, (e) LUAD, (f) PAAD, (g) PRAD, (h) SKCM, (i) STAD and (j) THCA.

Fig 4 reports for each investigated cancer type the KM plot of the most promising miRNA (namely the miRNA with lowest *p*-value) while the KM plots of the other miRNAs are reported in S1 Appendix. Except in the case of Pancreatic adenocarcinoma (PAAD) where the trend of the two populations is similar in the first 18 months, Fig 4 shows that the log-rank test always returns a *p*-value lower than 0.05 supporting the hypothesis that the survival probability of the two populations is statistically different (one with higher death risk and one with lower risk). This fact suggests that the expression value is suitable to be used to predict disease severity.

## Conclusion

In this paper, we presented a general-purpose pipeline to predict candidate biomarkers applied to 10 datasets of miRNA profiles of common cancers. Our approach consists of a sensible mix of existing tools for data mining based on the observation that the problem of finding biomarkers can be mapped into a two-class classification task. In particular, given the profiles of a cohort of control and tumor samples, find the subset of miRNAs that induces the highest classification accuracy. We solved this problem by means of: a differential evolution for the selection of candidates, component analysis to preserve the relationships among miRNAs, and SVM for sample classification.

Using real miRNAomes from The Cancer Genome Atlas we produced for each investigated cancer type a small list of putative biomarkers. Experiments highlighted that these panels have a very little overlap, suggesting that they can be used for cancer type classification purposes as well as they are predictive of the onset of the pathology. Comparisons proved that our method is more accurate than any other tested feature selection method as well as it provides an advantage over standard differential analysis done using NOISeqBIO. Yet, biological significance tests showed that most of the putative biomarker miRNAs have been reported to play a role in key oncogenesis pathways. Finally, using the Kaplan-Meier survival analysis we identified miRNAs which expression value can be correlated with cancer severity.

Summarizing: our pipeline has shown to have the potential to find sensible miRNA panels for cancer diagnosis and classification. The possibility of future releases of new miRNAomes could open to reuse our algorithm so as to further increase accuracy. Moreover, our pipeline has a value per se. In fact, we think that it can successfully be applied to other prediction tasks where the problem can be reformulated as a classification task.

## Supporting information

S1 Table Extended panel of miRNAs. List of miRNAs returned from at least one run of the DE algorithm. The list is sorted according to decreasing number of times it has been reported. The value of the *k*-th row refers to the classification accuracy obtained using the subset of the first *k* miRNAs.
(PDF)

S2 Table KEGG pathways. Most significant KEGG pathways associated with miRNA targeted genes.
(PDF)

S3 Table Cellular Component. Most significant Gene Ontology terms (Cellular Component) associated with miRNA targeted genes.
(PDF)

S4 Table Biological Process. Most significant Gene Ontology terms (Biological Process) associated with miRNA targeted genes.
(PDF)

S5 Table Molecular Function. Most significant Gene Ontology terms (Molecular Function) associated with miRNA targeted genes.
(PDF)

S1 Appendix Additional results. Detailed results of the experiments.
(PDF)
