# Supplementary material for: Genome-wide analysis of NGS data to compile cancer-specific panels of miRNA biomarkers
Source: PLoS One. 2018 Jul 26;13(7):e0200353. doi: 10.1371/journal.pone.0200353 (PMC6061989; doi:10.1371/journal.pone.0200353)
Supplement: S1 Table — List of miRNAs returned from at least one run of the DE algorithm. The list is sorted according to decreasing number of times it has been reported. The value of the k-th row refers to the classification accuracy obtained using the subset of the first k miRNAs. (PDF) [file pone.0200353.s001.pdf]

Rank	BRCA		KIRC		LGG		LIHC		LUAD	
1	hsa-mir-140	41.05	hsa-mir-542	56.99	hsa-mir-335	44.03	hsa-mir-10b	52.42	hsa-mir-140	47.69
2	hsa-mir-100	74.70	hsa-mir-3065	70.02	hsa-mir-148b	73.98	hsa-mir-92a-2	85.11	hsa-mir-103-1	90.39
3	hsa-mir-375	80.15	hsa-mir-361	74.81	hsa-mir-21	94.99	hsa-mir-20a	88.55	hsa-mir-195	93.75
4	hsa-mir-328	79.05	hsa-mir-374a	84.40	hsa-mir-155	96.38	hsa-mir-181a-1	91.09	hsa-mir-874	94.21
5	hsa-mir-744	88.50	hsa-mir-500a	92.50	hsa-mir-574	96.70	hsa-mir-17	91.09	hsa-mir-149	95.49
6	hsa-mir-324	93.87	hsa-mir-103-2	93.40	hsa-let-7e	97.97	hsa-mir-92a-1	91.35	hsa-mir-629	94.91
7	hsa-mir-30e	93.36	hsa-mir-18a	93.40	hsa-mir-455	97.87	hsa-mir-455	93.39	hsa-mir-141	95.02
8	hsa-mir-1307	95.85	hsa-mir-203	93.25	hsa-mir-128-1	97.76	**hsa-mir-34a**	**94.79***	hsa-mir-574	95.72
9	**hsa-mir-26a-2**	**96.93***	hsa-mir-576	96.15	**hsa-mir-424**	**98.97***	hsa-mir-589	93.39	hsa-let-7i	95.72
10	hsa-mir-582	96.34	**hsa-mir-29c**	**97.30***	hsa-mir-16-1	96.91	hsa-mir-29a	91.35	**hsa-mir-625**	**96.18***
11	hsa-mir-28	95.59	hsa-mir-625	95.80	hsa-mir-142	95.73	hsa-mir-16-1	92.62	hsa-mir-10b	95.02
12			hsa-mir-744	93.15			hsa-mir-374a	93.00	hsa-let-7d	93.98
13			hsa-mir-502	93.70			hsa-mir-199a-2	90.58	hsa-mir-9-1	93.86
14			hsa-mir-769	91.75			hsa-mir-181c	90.46	hsa-mir-542	91.66
15			hsa-mir-150	89.20					hsa-mir-181d	89.70
16			hsa-mir-339	85.45						

Rank	PAAD		PRAD		SKCM		STAD		THCA	
1	hsa-mir-582	69.71	hsa-mir-27b	47.18	hsa-mir-27b	47.80	hsa-mir-134	50.56	hsa-mir-660	46.63
2	hsa-mir-151	69.38	hsa-mir-1287	62.96	hsa-mir-30b	86.89	hsa-mir-381	63.69	hsa-mir-20a	83.71
3	hsa-mir-130b	76.64	hsa-mir-27a	79.39	hsa-mir-425	87.70	hsa-mir-142	73.48	hsa-mir-15a	83.95
4	hsa-mir-194-2	76.13	hsa-mir-342	82.59	hsa-mir-30a	90.60	hsa-mir-337	75.59	hsa-mir-93	87.64
5	hsa-mir-409	80.20	hsa-mir-146b	90.18	hsa-mir-155	90.25	hsa-mir-127	81.79	hsa-mir-576	85.80
6	hsa-mir-181a-1	87.98	hsa-mir-3065	90.41	hsa-let-7g	90.72	hsa-mir-409	80.05	hsa-let-7a-2	88.50
7	hsa-mir-598	94.09	hsa-mir-128-2	91.18	hsa-mir-576	93.85	hsa-mir-542	85.88	hsa-let-7a-1	91.87
8	hsa-mir-424	94.25	**hsa-mir-625**	**94.27***	**hsa-mir-99b**	**96.98***	hsa-mir-744	86.12	hsa-mir-24-2	92.06
9	**hsa-mir-574**	**96.44***	hsa-mir-374a	93.17	hsa-mir-152	96.64	hsa-mir-128-1	87.36	**hsa-mir-33a**	**93.28***
10	hsa-mir-145	95.60	hsa-mir-203	93.72	hsa-mir-99a	96.87	hsa-mir-199b	88.97	hsa-mir-335	92.74
11	hsa-mir-203	94.75			hsa-mir-23b	96.29	**hsa-mir-379**	**92.44***	hsa-mir-26a-2	91.22
12	hsa-mir-452	93.22			hsa-mir-199b	94.90	hsa-mir-99b	91.33	hsa-mir-598	91.65
13	hsa-mir-365-1	90.35			hsa-mir-181a-2	94.90	hsa-mir-152	91.33		
14	hsa-mir-181d	88.32					hsa-mir-136	90.94		
15	hsa-mir-194-1	86.12					hsa-mir-199a-2	90.34		
16							hsa-mir-652	87.73		
17							hsa-mir-222	88.23		

Table S1: List of miRNAs returned from at least one run of the DE algorithm. The list is sorted according to decreasing number of times it has been reported. The value of the k-esim row refears to the classification accuracy obtained using the subset of the first k miRNAs.
