# Supplementary material for: Genome-wide analysis of NGS data to compile cancer-specific panels of miRNA biomarkers
Source: PLoS One. 2018 Jul 26;13(7):e0200353. doi: 10.1371/journal.pone.0200353 (PMC6061989; doi:10.1371/journal.pone.0200353)
Supplement: S2 Table — Most significant KEGG pathways associated with miRNA targeted genes. (PDF) [file pone.0200353.s002.pdf]

pathway	Count	Genes
hsa04144: Endocytosis	53	WIPF1, WIPF2, CLTC, KIAA1033, CBLB, AP2A1, RAB22A, IGF1R, EEA1, PSD3, RAB8A, GIT1, SH3GL2, SH3GLB1, PDGFRA, VPS37D, TGFBR1, EPN2, DNM3, ACAP2, RAB7A, RAB5B, NEDD4L, ADRB1, ASAP1, VPS26B, ADRB2, CYTH2, PARD6B, SNX1, SNX2, GRK5, ERBB4, PIP5K1B, LDLR, SNX6, SMAD2, TGFB3, SMURF1, GBF1, WWP1, AP2B1, ARPC5, RAB11A, IGF2R, EHD1, RAB10, NEDD4, CAPZA1, IL2RA, CHMP2B, FGFR3, SMAP1
hsa04360: Axon guidance	34	ROBO2, NRP1, SEMA3A, SEMA3F, EFNA4, GNAI2, PPP3CA, PPP3R1, PPP3CB, DPYSL2, CFL2, PAK7, PLXNA2, ABL1, NCK2, PAK6, PLXNA1, SRGAP3, EPHB2, EPHB1, EPHB3, SEMA6B, NTNG1, ARHGEF12, SEMA6A, UNC5A, SEMA6D, LIMK1, UNC5C, UNC5D, EFNA3, RASA1, KRAS, EPHA3
hsa04810: Regulation of actin cytoskeleton	45	NCKAP1, ITGB3, PDGFA, PIK3CD, PIK3R2, FGD1, CRKL, FGF5, GNA13, PPP1CC, FGF7, FGF9, CFL2, PDGFC, PAK7, PIP4K2A, PAK6, PIP4K2B, PIP5K1B, WASF1, MYH10, GIT1, VAV3, PDGFRB, PDGFRA, ARHGEF12, PPP1R12A, ACTN1, LIMK1, ARPC5, GNG12, SSH2, ENAH, ABI2, ITGA10, MYH9, ITGA6, KRAS, ITGA5, FGFR3, CRK, VCL, FGF11, PFN2, ARHGEF6
hsa04120: Ubiquitin mediated proteolysis	35	DET1, UBE3C, CUL5, UBE2D2, CUL3, UBE2D3, CUL2, UBE2D1, NEDD4L, UBE3A, CBLB, RNF7, UBE2Z, RCHY1, UBE2J1, SOCS3, HERC3, HERC2, SOCS1, UBR5, UBE2F, UBE2I, SMURF1, SIAH1, WWP1, UBE2G1, PIAS1, DDB2, UBE2R2, NEDD4, UBA3, UBE2O, BIRC6, UBE2K, CUL4B
hsa05200: Pathways in cancer	67	SLC2A1, PIK3CD, CBLB, LAMC1, GLI3, GLI2, CRKL, IGF1R, FGF5, FGF7, EDNRA, RASSF1, EDNRB, FGF9, CASP3, JAK1, PDGFRB, PDGFRA, ARHGEF12, MITF, TGFBR1, RUNX1, AR, ADCY9, PLCB4, CCNE2, AGTR1, RARB, ITGA6, PLCB1, CRK, LAMA5, RALA, MAX, PTGER2, CUL2, TCF7, PDGFA, PIK3R2, ADCY1, RASGRP2, ADCY6, GNAI2, GNA13, GNG10, ABL1, E2F3, PLCG1, WNT1, RALGDS, RUNX1T1, SMAD2, FZD3, FZD5, TGFB3, FZD7, FZD8, GNG12, VEGFA, KITLG, BMP2, CCDC6, BCL2, KRAS, FGFR3, FGF11, BCL2L1

Table S2: Top *five* KEGG pathways associated with miRNA targeted genes in BRCA.

pathway	Count	Genes
hsa05200: Pathways in cancer	114	FGF1, IGF1R, FGF5, FGF7, EDNRA, EDNRB, AKT3, AKT1, EP300, PRKACB, PDGFRB, PDGFRA, MAP2K1, WNT5B, PRKCB, TPM3, WNT5A, PRKCA, RUNX1, ADCY9, COL4A2, CCNE2, COL4A1, COL4A4, COL4A3, COL4A6, COL4A5, EPAS1, PDGFB, TGFA, PIK3R3, PIK3R1, PLD1, STK4, HIF1A, FOXO1, ABL1, RALGDS, FZD1, SMAD2, CREBBP, JUN, TGFB2, FZD5, FZD4, TGFB3, FZD8, IGF1, GNG12, BMP2, CDK6, GNB2, CDK2, GNAS, GNB4, CYCS, FGF13, FGF12, FGFR2, RET, ITGB1, PTEN, LAMC2, LAMC1, GLI3, IKBKB, CASP3, ITGAV, RAC1, APPL1, ARHGEF12, DCC, ITGA2, MMP2, NCOA4, ARNT, FOS, WNT16, RHOA, TGFBR1, TRAF4, PIK3CA, TRAF3, TRAF5, KIT, RARB, ITGA6, PLCB1, CRK, MET, BIRC2, PPARD, RALA, LAMA2, PTGER2, GNAI3, XIAP, ADCY2, ADCY1, CBL, CDC42
hsa04510: Focal adhesion	73	ITGB1, TNC, PTEN, LAMC2, LAMC1, ARHGAP5, MYLK, ACTG1, IGF1R, PPP1CB, CCND2, AKT3, AKT1, ITGB8, ITGAV, RAC1, VAV3, PDGFRB, PDGFRA, MAP2K1, PPP1R12A, PRKCB, PDPK1, ACTN1, ITGA2, PRKCA, ACTN4, RHOA, COL2A1, COL4A2, PIK3CA, COL4A1, COL4A4, COL4A3, RAPGEF1, COL4A6, COL4A5, COL6A3, ITGA6, CRK, MET, BIRC2, VCL, ITGA9, LAMA2, SHC1, SRC, PXN, PDGFB, XIAP, PIK3R3, PIK3R1, THBS1, CDC42, MAPK9, PAK1, MAPK8, RAP1A, RELN, PDGFD, PDGFC, PAK7, JUN, CAV2, CAV1, PARVA, IGF1, VEGFA, COL1A1, MAPK10, COL1A2, ITGA11, COL9A1
hsa05205: Proteoglycans in cancer	67	ITGB1, ACTG1, IGF1R, PPP1CB, CASP3, AKT3, AKT1, IL12B, ITGAV, RAC1, PRKACB, MAP2K1, ARHGEF12, PPP1R12A, WNT5B, PRKCB, PDPK1, MMP2, ITGA2, WNT5A, GAB1, RRAS2, FRS2, PRKCA, ANK3, WNT16, ANK1, HSPG2, RHOA, TIAM1, PIK3CA, COL21A1, MET, HBEGF, CAMK2D, SRC, PXN, ITPR1, ITPR2, PIK3R3, PIK3R1, CBL, HIF1A, THBS1, CDC42, PAK1, ERBB3, ERBB4, CAMK2G, FZD1, SMAD2, TGFB2, FZD5, FZD4, CAV2, CAV1, RDX, FZD8, WNT7A, MSN, IGF1, ESR1, VEGFA, TFAP4, RPS6KB1, FAS, KRAS
hsa04068: FoxO signaling pathway	50	IRS1, SETD7, PTEN, PRKAG2, IRS2, GRM1, IGF1R, IKBKB, CCND2, AKT3, AKT1, EP300, RAG1, IL10, PRKAB2, GABARAPL1, MAP2K1, G6PC, PDPK1, SIRT1, TGFBR1, RBL2, PIK3CA, SGK3, SGK1, FOXG1, PIK3R3, FOXO4, PIK3R1, FOXO3, NLK, STK4, FOXO1, MAPK9, MAPK8, BCL2L11, SMAD2, TGFB2, CREBBP, HOMER1, TGFB3, GADD45A, PLK2, IGF1, MAPK10, G6PC2, CCNG2, CDK2, ATM, KRAS
hsa04151: PI3K-Akt signaling pathway	91	CHRM2, YWHAE, ITGB1, ATF2, CRTC2, IRS1, YWHAB, TNC, PTEN, LAMC2, LAMC1, FGF1, IGF1R, IKBKB, FGF5, GHR, TCL1A, FGF7, CCND2, YWHAQ, PPP2R5E, MYB, CREB3L2, AKT3, AKT1, ITGB8, ITGAV, RAC1, YWHAG, PDGFRB, PDGFRA, MAP2K1, G6PC, PDPK1, ITGA2, PRKCA, TSC1, RBL2, CREB3, COL2A1, CREB1, COL4A2, CCNE2, PIK3CA, COL4A1, COL4A4, COL4A3, KIT, COL4A6, COL4A5, COL6A3, SGK3, ITGA6, SGK1, EIF4E2, MET, CREB5, ITGA9, LAMA2, PDGFB, PIK3R3, PIK3R1, FOXO3, THBS1, PPP2CA, GNG2, BCL2L11, RELN, PDGFD, PDGFC, EIF4E, MCL1, IGF1, GNG12, VEGFA, COL1A1, G6PC2, COL1A2, CDK6, PPP2R2C, RPS6KB1, GNB2, ITGA11, CDK2, COL9A1, GNB4, PKN2, KRAS, FGF13, FGF12, FGFR2

Table S2: Top *five* KEGG pathways associated with miRNA targeted genes in KIRC.

pathway	Count	Genes
hsa05200: Pathways in cancer	131	RB1, SPI1, FGF1, FGF2, ETS1, CRKL, IGF1R, FGF7, EDNRB, CCND1, RASSF5, AKT3, EP300, PRKACA, PDGFRA, MAP2K1, RALBP1, WNT5B, TPM3, DAPK1, WNT5A, MITF, BCR, ADCY9, COL4A2, MSH2, COL4A1, CCNE1, AGTR1, COL4A6, COL4A5, RAF1, TP53, CSF1R, MAX, EPAS1, CUL2, PDGFB, TGFA, PIK3R3, PIK3R1, HIF1A, RASGRP1, FOXO1, HSP90B1, DVL1, DVL3, FADD, WNT1, SMAD2, WNT10B, TGFB1, SMAD3, FZD5, JUP, FZD4, WNT3A, PTCH1, FZD6, IGF1, GNG12, IL6, CDK6, GNAQ, CDK2, BCL2, GNAS, GRB2, FGF13, FGF12, FGF11, FGFR2, BCL2L1, FGFR1, RET, CDKN1A, CDKN1B, FLT3, PTEN, SLC2A1, FASLG, LAMC1, IKBKB, SUFU, CASP3, RAC1, NKX3-1, ARHGEF12, CHUK, ITGA2, NCOA4, ARNT, FOS, AXIN2, RHOA, TGFBR1, TGFBR2, PIK3CA, KIT, RARB, PLCB1
hsa05205: Proteoglycans in cancer	75	CDKN1A, ITGB3, FASLG, FGF2, IGF1R, PPP1CB, PPP1CC, CCND1, CASP3, AKT3, KDR, TIMP3, RAC1, PRKACA, MAP2K1, ARHGEF12, PPP1R12A, WNT5B, ITGA2, WNT5A, FRS2, ANK2, HSPG2, RHOA, DCN, VAV2, TIAM1, MRAS, PIK3CA, COL21A1, ITGA5, RAF1, PPP1R12B, TP53, TLR4, MET, ROCK1, CAMK2A, ITPR1, PIK3R3, PIK3R1, IQGAP1, CBL, HIF1A, THBS1, NRAS, PAK1, ERBB3, ERBB4, WNT1, CAMK2G, EIF4B, SMAD2, WNT10B, TGFB1, FZD5, LUM, FZD4, WNT3A, PTCH1, FZD6, RDX, STAT3, WNT7A, IGF1, MAPK14, ESR1, VEGFA, TFAP4, RPS6KB1, PDCD4, FAS, GRB2, KRAS, FGFR1
hsa04151: PI3K-Akt signaling pathway	102	CSF1, IRS1, FGF1, FGF2, IGF1R, CCND3, FGF7, CCND2, CCND1, PPP2R1B, PPP2R1A, MYB, CREB3L2, AKT3, KDR, IL6R, PDGFRA, MAP2K1, TSC1, COL4A2, COL4A1, CCNE1, COL4A6, COL4A5, RAF1, TP53, CSF1R, PDGFB, PIK3R3, PIK3R1, FOXO3, HSP90B1, BCL2L11, INSR, IGF1, GNG12, COL1A1, IL3, IL6, COL1A2, CDK6, ITGA10, ITGA11, CDK2, BCL2, GRB2, FGF13, FGF12, FGF11, FGFR2, FGFR1, BCL2L1, YWHAE, CDKN1A, CDKN1B, FLT1, YWHAB, ITGB3, PTEN, FASLG, LAMC1, IKBKB, GHR, YWHAQ, ITGB8, RAC1, JAK2, YWHAH, CHUK, ITGA2, PPP2R5C, OSMR, NGF, YWHAZ, COL2A1, PIK3CA, KIT, COL6A3, SGK3, ITGA5, SGK1, TLR4, MET, CREB5, ITGA9, PRKAA1, FIGF, TNXB, LAMA4, THBS1, RELA, NRAS, GNG2, RELN, RXRA, GNG5, EIF4B, VEGFA, RPS6KB1, PKN2
hsa04010: MAPK signaling pathway	83	PTPRR, ZAK, FASLG, FGF1, FGF2, DUSP16, CRKL, IKBKB, PPP3CA, RPS6KA3, PPP3CB, FGF7, RPS6KA5, CASP3, AKT3, MAP3K8, RAC1, PRKACA, MAP3K4, DUSP4, DUSP5, PDGFRA, MAP2K4, DAXX, MEF2C, MAP2K1, CHUK, DUSP1, FOS, NGF, DUSP9, TGFBR1, CDC25B, TGFBR2, CACNB1, CACNB2, MRAS, CACNB4, IL1B, RASA1, RAPGEF2, RAF1, TP53, MAX, SRF, PDGFB, CACNA1D, NLK, CACNA1E, RASGRP1, RELA, MAPK9, NRAS, PAK1, MKNK1, NTF3, GNA12, PAK2, MAP4K3, CACNG4, MAP4K4, MAP3K2, MAP3K3, NTRK2, TGFB1, MAP3K1, GADD45A, BDNF, NFATC3, MAPK14, GNG12, GADD45G, MAPK10, NF1, FAS, GRB2, KRAS, FGF13, MAP3K14, FGF12, FGF11, FGFR2, FGFR1
hsa04550: Signaling pathways regulating pluripotency of stem cells	57	BMPR2, FGF2, IGF1R, SOX2, ZIC3, AKT3, SMARCAD1, JARID2, JAK2, ACVR1, MAP2K1, WNT5B, WNT5A, LIFR, AXIN2, ISL1, DUSP9, POU5F1, PIK3CA, HAND1, RAF1, PIK3R3, PIK3R1, ACVR1B, NRAS, ACVR1C, DVL1, DVL3, OTX1, WNT1, SMAD2, WNT10B, SMAD1, ZFHX3, SMAD3, FZD5, PCGF5, FZD4, WNT3A, PCGF3, FZD6, STAT3, WNT7A, INHBB, IGF1, MAPK14, KLF4, SMAD5, ACVR2B, ACVR2A, MEIS1, APC, GRB2, KRAS, TCF3, FGFR2, FGFR1

Table S2: Top *five* KEGG pathways associated with miRNA targeted genes in LGG.

pathway	Count	Genes
hsa05200: Pathways in cancer	92	RB1, RET, CDKN1A, CDKN1B, PTEN, LAMC1, ETS1, FGF4, IGF1R, FGF7, CASP8, CCND1, MYC, AKT3, EP300, PRKACB, NKX3-1, JAK1, ARHGEF11, PDGFRA, MAP2K1, ARHGEF12, CHUK, PRKCB, MMP2, F2R, WNT5A, ARNT, MITF, AXIN2, PGF, RUNX1, TGFBR2, PAX8, PIK3CA, CCNE2, COL4A1, COL4A3, AGTR1, RARB, PLCB1, CRK, MET, PPARD, CSF1R, ROCK2, EPAS1, LEF1, PTGER3, TCF7, LAMA3, TGFA, XIAP, PIK3R3, PIK3R1, HIF1A, ADCY6, GNAI2, MAPK9, E2F1, DVL3, E2F2, MAPK1, E2F3, PLCG1, VHL, WNT1, RALGDS, FGF23, EGLN1, SMAD2, EGLN3, TCF7L1, FZD5, FZD4, FZD7, FZD6, STAT3, IGF1, VEGFA, NFKBIA, KITLG, BMP2, CDK6, BCL2, GNAS, FAS, CYCS, GNB5, FGF13, FGF12, F2RL3
hsa04144: Endocytosis	67	RET, FLT1, ZFYVE9, WIPF1, WIPF2, ARPC1A, WASL, RAB22A, IGF1R, EEA1, KIF5C, KIF5A, PSD3, KDR, LDLRAP1, PSD, SH3GLB1, PDGFRA, F2R, ZFYVE20, ARAP2, ARFGAP3, ARFGAP2, RNF41, TGFBR2, DNM2, DNM3, ACAP2, RABEP1, RAB35, CHMP7, MET, CSF1R, RAB5B, TSG101, SRC, AGAP2, NEDD4L, VPS26A, AGAP3, SNX4, PARD6B, RAB11FIP1, ERBB3, GRK6, AP2S1, PIP5K1A, LDLR, RAB11FIP4, CYTH1, RAB11FIP5, SMAD2, GIT2, HSPA8, RAB4A, IQSEC2, IQSEC1, CAV1, GBF1, AP2B1, RAB10, ITCH, DAB2, NEDD4, CAPZA1, SMAP1, SPG20
hsa05205: Proteoglycans in cancer	55	CDKN1A, ACTG1, IGF1R, PPP1CC, CCND1, MYC, AKT3, KDR, TIMP3, PRKACB, MAP2K1, ARHGEF12, PPP1R12A, PRKCB, MMP2, WNT5A, GAB1, FRS2, ANK2, ANK3, ANK1, TIAM1, PIK3CA, PPP1R12B, MET, DDX5, CAMK2D, ROCK2, SRC, SDC2, PIK3R3, PIK3R1, HIF1A, HOXD10, PAK1, RRAS, ERBB3, MAPK1, PLCG1, WNT1, CAMK2G, SMAD2, FZD5, FZD4, FZD7, CAV1, FZD6, STAT3, IGF1, MAPK14, ESR1, VEGFA, RPS6KB1, SDC1, FAS
hsa04360: Axon guidance	41	SEMA5A, ROCK2, NTN4, GNAI2, EFNB2, EFNB1, PPP3CA, ABLIM1, PAK1, PPP3R1, EFNB3, ABLIM3, DPYSL5, DPYSL2, CFL2, PAK7, NCK2, PLXNA1, MAPK1, PLXNC1, SRGAP3, SRGAP2, PAK2, EPHB4, EPHA5, NTNG1, EPHA4, EPHA7, ARHGEF12, SEMA6A, UNC5A, EPHA8, SEMA4B, SEMA4C, LIMK1, SEMA4F, SEMA4G, UNC5D, NFATC4, MET, EPHA2
hsa04919: Thyroid hormone signaling pathway	37	NOTCH2, NOTCH3, NOTCH1, THRA, SRC, PIK3R3, ATP1A2, PIK3R1, HIF1A, MED12L, ACTG1, MED17, MED13, PLN, CCND1, SIN3A, MYC, AKT3, EP300, MAPK1, PLCG1, SLC16A2, PRKACB, NCOA1, MED1, MAP2K1, PRKCB, NCOA3, ESR1, MED13L, RCAN1, KAT2B, NCOR1, PIK3CA, FXYD2, PLCB1, PFKP

Table S2: Top *five* KEGG pathways associated with miRNA targeted genes in LIHC.

pathway	Count	Genes
hsa05200: Pathways in cancer	131	RB1, FZD10, FGF2, CRKL, IGF1R, FGF5, FGF7, EDNRA, FGF9, CCND1, RASSF5, AKT3, EP300, PRKACB, PDGFRA, MAP2K1, PRKCB, DAPK1, HGF, WNT5A, PRKCA, MITF, RUNX1, BCR, ADCY9, COL4A2, CCNE2, COL4A1, CCNE1, COL4A6, COL4A5, RAF1, TP53, CTBP2, MAX, CUL2, PDGFB, PDGFA, PIK3R3, PIK3R1, RASGRP1, FOXO1, DVL1, ABL1, DVL3, WNT1, STAT5A, SMAD2, STAT5B, FZD3, JUN, TGFB2, SMAD3, FZD5, FZD4, WNT3A, PTCH1, FZD9, FZD8, IGF1, GNG12, NFKB1, BMP2, IL6, CXCL12, CDK6, GNAQ, BCL2, GNB4, GRB2, FGF13, FGF11, FGFR2, BCL2L1, FGFR1, RET, CDKN1A, FLT3, PTEN, SLC2A1, FASLG, LAMC1, GLI3, IKBKB, CASP3, ITGAV, APPL1, APC2, CHUK, ITGA2, MMP2, ARNT, FOS, AXIN2, WNT16, TGFBR1, PLCB4, RARB, ITGA6, CRK
hsa05205: Proteoglycans in cancer	80	CDKN1A, ITGB3, HSPB2, FASLG, FZD10, ELK1, FGF2, IGF1R, PPP1CC, CCND1, CASP3, AKT3, KDR, ITGAV, PRKACB, MAP2K1, PPP1R12A, PRKCB, HGF, MMP2, ITGA2, WNT5A, GAB1, FRS2, PRKCA, ANK2, WNT16, ANK1, HSPG2, VAV2, TIAM1, RAF1, PPP1R12B, TP53, TLR4, PPP1R12C, CD44, HBEGF, DDX5, SRC, SDC2, ITPR1, ITPR2, PIK3R3, PIK3R1, IQGAP1, CBL, THBS1, EGFR, CDC42, NRAS, PAK1, ERBB4, GPC3, WNT1, CAMK2G, EIF4B, SMAD2, FZD3, TGFB2, FZD5, FZD4, WNT3A, PTCH1, RDX, FZD9, IGF2, FZD8, WNT7A, IGF1, ESR1, VEGFA, PPP1CA, TFAP4, RPS6KB1, PDCD4, CTNNB1, FAS, GRB2, FGFR1
hsa04151: PI3K-Akt signaling pathway	111	CSF3, CRTC2, IRS1, PPP2R2A, FGF2, IGF1R, FGF5, CCND3, FGF7, CCND2, CCND1, FGF9, PPP2R1B, PPP2R1A, TNN, CREB3L1, MYB, AKT3, KDR, IFNAR2, PDGFRA, MAP2K1, G6PC, HGF, PRKCA, TSC1, COL4A2, CCNE2, COL4A1, CCNE1, COL4A6, MTCP1, COL4A5, RAF1, TP53, EPHA2, IFNAR1, PDGFB, PDGFA, PIK3R3, PIK3R1, EFNA4, BCL2L11, EIF4EBP1, NGFR, INSR, IGF1, GNG12, NFKB1, COL1A1, IL3, IL6, COL1A2, CDK6, RHEB, ITGA10, ITGA11, GNB4, BCL2, GRB2, FGF13, FGF11, FGFR2, FGFR1, BCL2L1, YWHAE, CDKN1A, FLT1, ITGB3, PTEN, FASLG, BRCA1, LAMC1, IKBKB, GHR, GYS1, YWHAQ, PPP2R5E, ITGB8, ITGAV, YWHAG, YWHAH, CHUK, ITGA2, PPP2R5C, OSMR, NGF, YWHAZ, CREB1, ITGA6, SGK1, TLR4, CREB5, FIGF, TNXB, THBS1, EGFR, PPP2CA, NRAS, GNG2
hsa04010: MAPK signaling pathway	86	PTPRR, ZAK, FASLG, ELK1, FGF2, DUSP16, CRKL, IKBKB, FGF5, PPP3CA, RPS6KA3, PPP3CB, FGF7, FGF9, CASP3, AKT3, PRKACB, MAP3K7, MAP3K4, DUSP4, PDGFRA, MAP2K4, MAP2K1, DUSP3, CHUK, PRKCB, DUSP1, PRKCA, FOS, NGF, DUSP9, DUSP6, TGFBR1, CDC25B, CACNB1, IL1A, CACNB2, PPM1A, PPM1B, RAPGEF2, RAF1, TP53, CRK, MAX, SRF, PDGFB, PDGFA, CACNA1D, CACNA1C, NLK, CACNA1E, RASGRP1, EGFR, STK3, RAP1B, CDC42, CACNA1I, MAPK9, PPP3R1, NRAS, PAK1, RAP1A, MKNK1, GNA12, MAP2K7, MAP4K3, CACNG4, MAP4K4, MAP3K3, NTRK2, JUN, TGFB2, MAP3K1, BDNF, NFATC3, GNG12, NFKB1, MAPK10, PPP5C, NF1, FAS, GRB2, FGF13, FGF11, FGFR2, FGFR1
hsa04390: Hippo signaling pathway	62	YWHAE, BMPR2, WWC1, PPP2R2A, FZD10, LIMD1, AMOT, BBC3, SOX2, PPP1CC, CCND3, CCND2, CCND1, YWHAQ, PPP2R1B, PPP2R1A, BTRC, TEAD1, YWHAG, TEAD2, TEAD3, YWHAH, APC2, WNT5A, CSNK1E, AXIN2, WNT16, YWHAZ, TGFBR1, LATS2, FRMD6, YAP1, NKD1, STK3, SAV1, PPP2CA, PARD6B, DVL1, CTNNA1, DVL3, WNT1, SMAD2, TGFB2, FZD3, SMAD3, FZD5, FZD4, WNT3A, FZD9, FZD8, WNT7A, GDF6, PPP1CA, SMAD7, BMP2, DLG2, DLG3, PPP2R2C, DLG4, PPP2R2B, CTNNB1, BMPR1A

Table S2: Top *five* KEGG pathways associated with miRNA targeted genes in LUAD.

pathway	Count	Genes
hsa05200: Pathways in cancer	95	RB1, RET, CDKN1A, WNT2B, FLT3, PTEN, SLC2A1, LAMC1, FGF2, CRKL, IGF1R, IKBKB, FGF7, CCND1, RASSF5, AKT3, EP300, PRKACA, PRKACB, APPL1, PDGFRA, MAP2K1, RALBP1, ARHGEF12, CHUK, WNT5B, TPM3, ITGA2, NCOA4, WNT5A, ARNT, MITF, AXIN2, TGFBR1, TGFBR2, BCR, CCNE1, COL4A4, COL4A3, KIT, RARB, PPARG, PLCB1, RAF1, CRK, MET, FIGF, ROCK1, EPAS1, PTGER2, CUL2, GNAI3, TGFA, PIK3R3, ADCY2, ADCY1, PIK3R1, HIF1A, FOXO1, MAPK9, MAPK8, RXRA, DVL1, GNA12, E2F1, E2F2, MAPK1, E2F3, PLCG1, WNT1, RUNX1T1, WNT10A, FZD3, EGLN3, EGLN2, SMAD3, FZD4, WNT3A, PTCH1, FZD6, WNT7A, IGF1, PTK2, VEGFA, MAPK10, IL6, CDK6, APC, GNAQ, CCDC6, BCL2, GRB2, KRAS, FGFR2, FGFR1
hsa05205: Proteoglycans in cancer	60	CDKN1A, WNT2B, TNF, FGF2, IGF1R, CCND1, AKT3, KDR, PRKACA, PRKACB, MAP2K1, ARHGEF12, WNT5B, ITGA2, WNT5A, FRS2, ANK2, ANK1, HSPG2, VAV2, TIAM1, ITGA5, RAF1, PPP1R12B, MET, ROCK1, CAMK2A, ITPR1, TWIST1, PIK3R3, PIK3R1, IQGAP1, HIF1A, HOXD10, PAK1, ERBB3, ERBB4, MAPK1, PLCG1, WNT1, CAMK2G, EIF4B, WNT10A, FZD3, FZD4, WNT3A, PTCH1, FZD6, WNT7A, IGF1, MAPK14, ESR1, PTK2, VEGFA, TFAP4, RPS6KB1, PDCD4, GRB2, KRAS, FGFR1
hsa04310: Wnt signaling pathway	46	WNT2B, CAMK2A, PRICKLE2, PSEN1, NKD1, LRP6, MAPK9, CCND3, PPP3R1, PPP3CB, MAPK8, CCND2, CCND1, WIF1, DVL1, EP300, BTRC, PRKACA, WNT1, MAP3K7, PRKACB, CAMK2G, SKP1, WNT10A, FZD3, SMAD3, WNT5B, FBXW11, FZD4, WNT3A, WNT5A, CTNNBIP1, SIAH1, FZD6, NFATC3, WNT7A, AXIN2, SENP2, NFATC4, MAPK10, VANGL1, FOSL1, VANGL2, APC, TBL1XR1, PLCB1
hsa05166: HTLV-I infection	65	RB1, NRP1, CD40, CDKN1A, WNT2B, SLC2A1, CD3E, TNF, IKBKB, PPP3CB, CCND3, CDC23, CCND2, CCND1, MYB, CHEK1, AKT3, EP300, PRKACA, PRKACB, MYBL1, PDGFRA, MAP2K4, TBP, WNT5B, CHUK, IL15, WNT5A, TGFBR1, TGFBR2, CANX, TBPL1, SRF, CREM, PIK3R3, ADCY2, ADCY1, PIK3R1, PPP3R1, MAPK8, DVL1, TP53INP1, E2F1, E2F2, E2F3, WNT1, MAP3K3, WNT10A, FZD3, RANBP3, MAP3K1, SMAD3, FZD4, WNT3A, FZD6, NFATC3, WNT7A, NFATC4, FOSL1, POLE4, IL6, APC, KRAS, TCF3, MAP3K14
hsa04550: Signaling pathways regulating pluripotency of stem cells	44	WNT2B, BMPR2, DLX5, PIK3R3, PIK3R1, FGF2, IGF1R, AKT3, DVL1, SMARCAD1, MAPK1, OTX1, JARID2, WNT1, ACVR1, SMAD1, WNT10A, FZD3, MAP2K1, ZFHX3, SMAD3, WNT5B, PCGF5, FZD4, WNT3A, WNT5A, FZD6, WNT7A, INHBB, IGF1, AXIN2, MAPK14, SMAD5, ACVR2B, ACVR2A, MEIS1, APC, GRB2, KRAS, TCF3, RAF1, NEUROG1, FGFR2, FGFR1

Table S2: Top *five* KEGG pathways associated with miRNA targeted genes in PAAD.

pathway	Count	Genes
hsa04010: MAPK signaling pathway	53	ZAK, FGF1, ELK1, FGF5, FGF7, RPS6KA5, RPS6KA1, MAP3K8, PRKACA, MAP3K4, DUSP5, PDGFRA, MAP2K4, MEF2C, CACNA2D3, MAPK8IP3, DUSP6, TGFBR1, CACNB2, MAPKAPK3, TRAF6, RASA1, RAPGEF2, MAPT, TP53, MAX, SRF, CACNA1C, NLK, EGFR, STK3, RAP1B, CDC42, PPP3R1, NRAS, MAPK7, MKNK2, FLNC, MAP2K7, PAK2, NTRK2, BDNF, MAPK14, GNG12, NFKB1, TAOK1, NF1, GRB2, KRAS, FGF13, MAP3K14, FGF12, MAP3K12
hsa05200: Pathways in cancer	68	RET, CBLB, LAMC2, FGF1, FGF5, FGF7, EDNRA, RASSF1, EDNRB, RASSF5, EP300, PRKACA, APPL1, PDGFRA, ITGA2, DAPK2, TGFBR1, RUNX1, TRAF6, COL4A3, RARA, COL4A6, RARB, PPARG, MET, TP53, PTGER4, MAX, CUL2, PTGER3, GNAI3, ADCY3, ADCY6, EGFR, FOXO1, CDC42, NRAS, RXRA, TPR, DVL2, E2F3, FADD, RUNX1T1, FZD1, SMAD2, WNT10B, SMAD4, FZD5, FZD4, WNT3A, FZD7, PTCH1, FN1, VEGFC, IGF1, GNG12, NFKB1, VEGFA, KITLG, CDK6, APC, GNAQ, CCDC6, GRB2, KRAS, FGF13, FGF12, BCL2L1
hsa05205: Proteoglycans in cancer	44	CAMK2D, SDC2, CAMK2A, CBLB, ELK1, EGFR, CDC42, PPP1CC, NRAS, ERBB4, TIMP3, FLNC, PRKACA, FZD1, SMAD2, WNT10B, FZD5, FZD4, WNT3A, PDPK1, FZD7, PTCH1, ITGA2, FN1, GAB1, FRS2, ANK2, ANK3, IGF1, MAPK14, ANK1, VAV2, VEGFA, PPP1CA, TIAM1, RPS6KB1, COL21A1, GRB2, KRAS, ITGA5, TP53, PPP1R12C, MET, HBEGF
hsa04360: Axon guidance	34	NRP1, SEMA7A, SEMA3B, LRRC4, SEMA3G, GNAI3, SEMA3E, ROBO1, EFNB2, CDC42, NRAS, PPP3R1, CFL2, PAK6, FYN, SRGAP3, SRGAP2, EPHB2, PAK2, EPHB1, EPHB3, EPHA4, SEMA6A, SEMA6D, LIMK2, SEMA4C, LIMK1, SEMA4F, UNC5D, EFNA3, RASA1, KRAS, MET, EPHA2
hsa04015: Rap1 signaling pathway	44	CSF1, CTNND1, GNAI3, ADCY3, FGF1, ADCY6, EGFR, FYB, RAP1B, FGF5, CDC42, PARD6B, NRAS, FGF7, CNR1, RASSF5, MAGI1, NGFR, PDGFRA, PRKCI, INSR, MAGI3, MAGI2, VEGFC, IGF1, MAPK14, VAV2, VEGFA, GRIN1, TIAM1, KITLG, EFNA3, PRKD3, ADORA2B, GNAQ, RAPGEF2, CALM3, KRAS, TLN2, FGF13, FGF12, MET, PFN2, EPHA2

Table S2: Top *five* KEGG pathways associated with miRNA targeted genes in PRAD.

pathway	Count	Genes
hsa05200: Pathways in cancer	122	RB1, SPI1, CBLB, FGF1, ETS1, CRKL, IGF1R, FGF5, FGF7, EDNRA, EDNRB, FGF9, CCND1, AKT2, RASSF5, PDGFRB, PDGFRA, RALBP1, TPM3, DAPK1, WNT5A, MITF, RUNX1, AR, ADCY9, COL4A2, CCNE2, COL4A1, COL4A4, AGTR1, COL4A6, COL4A5, TP53, CSF1R, CUL2, PDGFB, PDGFA, PIK3R2, PIK3R1, HIF1A, RASGRP1, FOXO1, TPR, ABL1, DVL2, DVL3, PLCG1, FADD, WNT1, RALGDS, SMAD2, FZD3, FZD5, FZD4, WNT3A, FZD7, PTCH1, FN1, FZD8, IGF1, GNG12, IL6, CDK6, BCL2, GNAS, GRB2, FGF13, FGF12, FGFR3, FGF11, BCL2L1, RET, CDKN1A, PIK3CD, FASLG, GLI2, SUFU, CASP3, ITGAV, RAC1, NKX3-1, JAK1, CHUK, ITGA2, FOS, RHOA, TGFBR1, TGFBR2, PLCB4, PIK3CA, RARA, RARB, PPARG, ITGA6, MET, PTGER4, RALB, PTGER2, PTGER3, ADCY3
hsa04010: MAPK signaling pathway	83	ZAK, FASLG, FGF1, DUSP16, CRKL, FGF5, PPP3CA, RPS6KA3, PPP3CB, FGF7, RPS6KA5, FGF9, AKT2, CASP3, RPS6KA2, STMN1, RAC1, MAP3K4, MAP3K5, DUSP4, PDGFRB, DUSP5, PDGFRA, MAP2K4, MEF2C, DUSP3, CHUK, DUSP1, CACNA2D3, RRAS2, FOS, NGF, MAPK8IP3, DUSP9, TGFBR1, TGFBR2, CACNB1, IL1A, CACNB2, MAPKAPK3, RASA1, RAPGEF2, MAPT, TP53, PDGFB, PDGFA, CACNA1D, CACNA1C, NLK, CACNA1E, RASGRP1, RELA, EGFR, RAP1B, CDC42, PPP3R1, NRAS, PAK1, MAPK7, MKNK2, MAP2K7, PAK2, MAP4K3, CACNG4, MAP4K4, MAP3K2, MAP3K3, NTRK2, MAP3K1, BDNF, MAPK14, GNG12, TAOK1, NF1, FAS, GRB2, KRAS, FGF13, MAP3K14, FGF12, FGFR3, FGF11, MAP3K12
hsa04151: PI3K-Akt signaling pathway	99	CHRM1, CSF1, IRS1, TNC, FGF1, IGF1R, FGF5, FGF7, CCND2, CCND1, FGF9, AKT2, MYB, PDGFRB, IFNAR2, PDGFRA, TSC1, PRLR, COL4A2, CCNE2, COL4A1, COL4A4, DDIT4, COL4A6, COL4A5, TP53, EPHA2, CSF1R, PDGFB, PDGFA, PIK3R2, PIK3R1, FOXO3, BCL2L11, NGFR, INSR, FN1, IGF1, GNG12, COL1A1, EFNA3, IL6, COL1A2, G6PC3, CDK6, IL7, BCL2, COL9A3, GRB2, FGF13, FGF12, FGFR3, FGF11, BCL2L1, YWHAE, CDKN1A, YWHAB, ITGB3, PIK3CD, FASLG, GHR, YWHAQ, PPP2R5E, ITGB8, ITGAV, RAC1, JAK2, JAK1, CHUK, PDPK1, ITGA2, OSMR, NGF, YWHAZ, RBL2, CREB1, PIK3CA, SGK3, ITGA6, ITGA5, MET, CREB5, PRKAA2, TNXB, THBS2, THBS1, RELA, EGFR, NRAS, RELN, RXRA, GNG5, VEGFC, VEGFA, KITLG, RPS6KB1, IL2RA, PKN2, KRAS
hsa04015: Rap1 signaling pathway	70	DOCK4, CSF1, ITGB3, CTNND1, PIK3CD, FGF1, CRKL, IGF1R, FGF5, FGF7, FGF9, AKT2, RASSF5, RAC1, PDGFRB, MAGI1, PDGFRA, MAGI3, MAGI2, NGF, RHOA, VAV2, ADCY9, PLCB4, PIK3CA, PRKD3, ADORA2B, RAPGEF2, RAPGEF5, MET, PFN2, EPHA2, RAPGEF4, CSF1R, RALB, PDGFB, ADCY3, PDGFA, PIK3R2, ADCY1, PIK3R1, MLLT4, THBS1, ADCY6, EGFR, GNAI2, RAP1B, CDC42, PARD6B, NRAS, CNR1, PLCG1, RALGDS, NGFR, INSR, VEGFC, IGF1, MAPK14, VEGFA, GNAO1, KITLG, EFNA3, GNAS, CALM3, KRAS, CALM1, FGF13, FGF12, FGFR3, FGF11
hsa04014: Ras signaling pathway	73	CSF1, PIK3CD, FASLG, FGF1, ETS1, IGF1R, FGF5, FGF7, FGF9, AKT2, RASSF5, RAC1, PDGFRB, PDGFRA, RALBP1, KSR1, CHUK, RRAS2, GAB2, NGF, RHOA, PIK3CA, RASA1, RAPGEF5, MET, EPHA2, SHC4, CSF1R, RALB, RAB5C, PDGFB, PLA2G3, PDGFA, PIK3R2, PIK3R1, RASAL2, RASGRP1, MLLT4, RELA, EGFR, RAP1B, CDC42, NRAS, PAK1, GNG5, PAK7, ABL1, PAK6, ABL2, SHOC2, PLCG1, PAK2, RALGDS, NGFR, INSR, VEGFC, IGF1, GNG12, VEGFA, KITLG, EFNA3, NF1, CALM3, GRB2, KRAS, CALM1, RGL1, FGF13, RGL2, FGF12, FGFR3, FGF11, BCL2L1

Table S2: Top *five* KEGG pathways associated with miRNA targeted genes in SKCM.

pathway	Count	Genes
hsa05205: Proteoglycans in cancer	35	ITGB1, ROCK2, PXN, PIK3CD, CBL, HIF1A, ACTG1, IGF1R, ERBB4, GPC1, ITGAV, RAC1, PRKACB, CAMK2G, ARHGEF12, PPP1R12A, FZD4, PRKCB, FZD7, PTCH1, CAV1, FZD6, STAT3, WNT5A, FRS2, ANK2, ANK3, MAPK14, ESR1, PTK2, VEGFA, RPS6KB1, KRAS, MET, HBEGF
hsa04550: Signaling pathways regulating pluripotency of stem cells	27	GSK3B, BMPR2, PIK3CD, ACVR1B, IGF1R, SOX2, DVL2, SMARCAD1, OTX1, JAK2, ACVR1, ZFHX3, PCGF5, FZD4, FZD7, PCGF3, WNT5A, FZD6, STAT3, MAPK14, KLF4, ACVR2B, ACVR2A, ID2, ID1, KRAS, FGFR2
hsa05200: Pathways in cancer	50	RB1, RET, ITGB1, GSK3B, PIK3CD, LAMC2, LAMC1, FGF1, ETS1, IGF1R, FGF7, ITGAV, RAC1, PRKACB, APPL1, ARHGEF12, PRKCB, WNT5A, MITF, TGFBR1, PAX8, COL4A1, ITGA6, CRK, MET, CEBPA, ROCK2, LEF1, TCF7, XIAP, STK4, CBL, HIF1A, GNAI1, DVL2, E2F3, CTNNA2, STAT5B, FZD4, FZD7, PTCH1, FZD6, STAT3, PTK2, VEGFA, NFKBIA, RAD51, GNB4, KRAS, FGFR2
hsa04360: Axon guidance	25	ITGB1, GSK3B, SEMA3C, ROCK2, SEMA3A, NTN4, LRRC4, SEMA3F, EFNA5, GNAI1, RND1, EFNB2, PLXNA2, PLXNA1, RAC1, SRGAP2, NTNG1, EPHA7, ARHGEF12, SEMA6A, UNC5A, SEMA6D, PTK2, KRAS, MET
hsa04520: Adherens junction	17	YES1, CSNK2A1, INSR, LEF1, TCF7, SORBS1, BAIAP2, NLK, TGFBR1, ACTG1, IGF1R, SNAI1, CTNNA2, RAC1, WASF1, MET, PVRL1

Table S2: Top *five* KEGG pathways associated with miRNA targeted genes in STAD.

pathway	Count	Genes
hsa05200: Pathways in cancer	110	RB1, FZD10, FGF1, FGF2, FGF4, CRKL, IGF1R, FGF5, FGF7, EDNRB, CCND1, RASSF5, AKT3, EP300, PRKACA, PRKACB, PDGFRA, MAP2K1, TPM3, F2R, WNT5A, RUNX1, BCR, ADCY9, MSH2, CCNE2, COL4A1, CCNE1, COL4A4, COL4A3, RAF1, EPAS1, CUL2, TCF7, PIK3R3, PIK3R1, HIF1A, FOXO1, DVL1, STAT5A, SMAD2, JUN, SMAD3, FZD4, WNT3A, FZD7, PTCH1, FZD6, IGF1, BMP2, CDK6, BCL2, GNAS, CYCS, GRB2, GNB5, FGFR2, FGFR1, RET, CDKN1A, FLT3, PTEN, LAMC1, IKBKB, CASP8, ITGAV, NKX3-1, JAK1, ARHGEF11, ARHGEF12, CHUK, ITGA2, MMP2, ARNT, FOS, AXIN2, TGFBR1, TGFBR2, PIK3CA, RARB, ITGA6, PLCB1, CRK, PPARD, FIGF, ROCK2, PTGER3, LAMA3, GNAI3, XIAP, CDC42, MAPK9, GNG2, GNA12, E2F1, E2F2, MAPK1, E2F3, VHL, RUNX1T1
hsa04144: Endocytosis	83	RET, ARF1, FLT1, ZFYVE9, WIPF1, WIPF2, CLTC, KIAA1033, AP2A1, WASL, RAB22A, IGF1R, EEA1, KIF5C, KIF5B, KIF5A, PSD3, KDR, LDLRAP1, GIT1, SH3GL2, PSD, PDGFRA, PDCD6IP, F2R, ZFYVE20, ARAP2, ARFGAP3, TGFBR1, EPN1, ARFGAP2, RNF41, TGFBR2, DNM2, ACAP2, ZFYVE16, RABEP1, CHMP4C, RAB5B, TSG101, VPS4A, NEDD4L, VPS26A, ASAP1, ADRB2, AGAP3, CDC42, SNX4, PARD6B, RAB11FIP1, ERBB3, ERBB4, RAB11FIP2, LDLR, RAB11FIP4, RAB11FIP5, SNX6, SMAD2, GIT2, HSPA8, SMAD3, IQSEC2, SMURF2, IQSEC1, SMURF1, VTA1, CAV1, GBF1, WWP1, AP2B1, IGF2R, RAB10, ITCH, DAB2, CAPZA1, CAPZA2, IL2RB, VPS45, RAB5A, SMAP1, FGFR2, HSPA1A, SPG20
hsa05205: Proteoglycans in cancer	65	CDKN1A, FZD10, ELK1, FGF2, IGF1R, CCND1, AKT3, KDR, TIMP3, ITGAV, PRKACA, PRKACB, MAP2K1, ARHGEF12, MMP2, ITGA2, WNT5A, GAB1, FRS2, ANK2, ANK3, HSPG2, VAV2, TIAM1, PIK3CA, RAF1, PPP1R12B, CD44, DDX5, CAMK2D, ROCK2, SDC2, ITPR1, PIK3R3, PIK3R1, IQGAP1, HIF1A, HOXD10, CDC42, ERBB3, ERBB4, MAPK1, CAMK2G, EIF4B, SMAD2, FZD4, WNT3A, FZD7, PTCH1, CAV1, FZD6, STAT3, WNT7A, MSN, IGF1, MAPK14, ESR1, VEGFA, TFAP4, RPS6KB1, PDCD4, SDC1, FAS, GRB2, FGFR1
hsa04010: MAPK signaling pathway	74	PTPRR, ZAK, FGF1, ELK1, FGF2, CRKL, FGF4, IKBKB, FGF5, RPS6KA4, PPP3CA, RPS6KA3, PPP3CB, FGF7, RPS6KA5, RPS6KA2, AKT3, RPS6KA1, MAP3K8, PRKACA, PRKACB, MAP3K7, MAP3K4, MAP3K5, PDGFRA, MAP2K4, DUSP2, MEF2C, MAP2K1, CHUK, FOS, DUSP8, TGFBR1, CDC25B, TGFBR2, CACNB1, PPM1A, PPM1B, RASA1, RAPGEF2, RAF1, CRK, CACNA1C, NLK, CACNA1E, CDC42, MAPK9, PPP3R1, MKNK1, MKNK2, GNA12, MAPK1, PAK2, MAP4K4, MAP3K2, MAP3K3, HSPA8, NTRK2, JUN, MAP3K1, BDNF, NFATC3, MAPK14, TAOK3, TAOK2, NF1, FAS, GRB2, MAP3K14, MAP3K11, FGFR2, HSPA1A, FGFR1, MAP3K12
hsa04310: Wnt signaling pathway	48	CAMK2D, ROCK2, TCF7, CUL1, PRICKLE2, FZD10, NLK, NKD1, LRP6, PPP3CA, MAPK9, CCND3, PPP3R1, PPP3CB, CCND2, CCND1, WIF1, DVL1, EP300, GPC4, TBL1X, BTRC, PRKACA, MAP3K7, PRKACB, CAMK2G, JUN, TCF7L1, SMAD3, FBXW11, FZD4, WNT3A, SERPINF1, FZD7, WNT5A, CTNNBIP1, SIAH1, FZD6, NFATC3, WNT7A, AXIN2, SENP2, VANGL1, FOSL1, TBL1XR1, BAMBI, PLCB1, PPARD

Table S2: Top *five* KEGG pathways associated with miRNA targeted genes in THCA.
