# Supplementary material for: Genome-wide analysis of NGS data to compile cancer-specific panels of miRNA biomarkers
Source: PLoS One. 2018 Jul 26;13(7):e0200353. doi: 10.1371/journal.pone.0200353 (PMC6061989; doi:10.1371/journal.pone.0200353)
Supplement: S3 Table — Most significant Gene Ontology terms (Cellular Component) associated with miRNA targeted genes. (PDF) [file pone.0200353.s003.pdf]

Cellular Component	Count	Genes
GO:0005829 cytosol	326	NCKAP1, TRIO, WIPF1, WIPF2, ITSN1, DIXDC1, CBLB, BACH1, CRKL, RPS6KA3, GJA1, PSMD7, RPS6KA2, DPYSL2, DPYSL3, PIP4K2A, PIP4K2B, ARFIP1, ATP6V1E2, GLUL, TP63, CSNK2A1, ACTN1, AP1B1, ANK2, FNDC3A, ANK1, EML1, SRRM1, PSME3, ULK2, PRKD1, ANKRA2, CLOCK, SEH1L, MTMR3, STXBP1, HSPA4L, EIF5A2, NEDD4L, PIK3R2, VPS26B, NLK, MTMR4, LDHB, PPP3R1, SNX1, RIC8B, HLCS, PDCD10, PIP5K1B, PLCG1, PPARGC1A, CEP76, RALGDS, ATP6V1C2, LYN, YES1, CHKA, STAC, SIAH1, RAB3IP, PLK1, MYO5A, NET1, CCDC88A, PPP5C, STAG1, STAG2, RPS29, NMT1, HNRNPC, RGL1, AARS, ARF4, NRP1, ITK, CLTC, AP2A1, HIF3A, CLINT1, LYST, SMG5, SH3RF1, GLI3, BBC3, EEA1, MECP2, RPTOR, PPP3CA, GYS1, ACTR1A, PPP3CB, AKAP10, XPO1, C1QBP, NEFL, JAK2, JAK1, NDEL1
GO:0005654 nucleoplasm	156	CCNT2, MAML1, UBE2D2, FMR1, UBE2D3, UBE2D1, EHMT1, NR3C1, NR3C2, RPS6KA3, PSMD7, RPS6KA2, PAPOLA, DAG1, KPNA3, JUNB, TP63, MED1, ESCO1, REV1, CDC25A, NRBP1, PIAS1, SRRM1, AR, CCNE2, PSME3, ERF, PGR, ZFPM2, KPNB1, SNRPB, NOTCH2, NOTCH1, CUL2, GATA6, NEDD4L, GATA3, FOXO4, PDS5B, PDS5A, NLK, FOXO3, NEUROD1, PPP3R1, ATXN1, NRBF2, PPARGC1A, PPARGC1B, NKX2-2, SMAD2, ESRRA, SMAD1, PHC2, UBE2I, BRPF1, SMURF1, CBX2, PLK1, SMARCA5, FANCC, NR1D2, ESRRG, NR1D1, SMAD7, NR4A2, STAG1, STAG2, SP1, MAFG, TAF4B, HNRNPC, RAD9B, OGT, RCOR1, RARG, THRB, ELL, PHF20, CHD9, HNF4G, RSF1, HIF3A, YBX1, CTCF, BMI1, PHF6, LMNB1, PPP3CA, PPP3CB, XPO1, MED13, ING3, HEY1, CASP3, ENSA, MYBL2, TEAD1, RBM5, NCOA1
GO:0000785 chromatin	54	SUV39H2, RARG, THRB, SETD3, ELL, AFF4, MECP2, FAM178A, JUNB, ZNF385A, TP63, NCOA1, MED1, HELLS, ESCO1, EED, TCF12, H2AFX, DNMT3A, PAX6, ARID1A, SIRT1, RUNX2, TOX4, NCOR2, AR, MAF, CREB1, WDR82, TAL1, HAND2, PLCB1, SMARCD2, SATB1, BAZ2A, GATA3, PDS5B, PDS5A, HLCS, SMAD2, PHC2, JUND, CBX3, CBX2, NR1D1, KLF4, ASXL1, STAG1, STAG2, NEDD4, SNAI2, HNRNPC, RAN, NFE2L2
GO:0005911 cell-cell junction	60	ITK, OXTR, B4GALT1, SLC2A1, ILK, PTPRK, SLC8A1, CALB2, TMEM47, PNN, GJA1, DAG1, PGM5, SCN1A, DSP, MAGI3, ACTN1, MAGI2, SHROOM3, FRS2, ANK2, FRMD4A, TGFBR1, CD2AP, CLDN11, FRMD6, RAPGEF2, MYH9, ITGA6, ITGA5, PRKD1, ARHGEF2, SSX2IP, VCL, ARHGEF6, CAMK2D, AHNAK, COL13A1, TWF1, LIN7C, MTDH, RAP1B, PARD6B, UBN1, NHS, PLCG1, STX2, CADM3, FZD5, PCDH9, PLEKHA7, SMAD7, TJP1, DLG3, VAPA, ABI2, DLG5, AMOTL2, AMOTL1, SCN2A
GO:0005912 adherens junction	68	NRP1, NCKAP1, OXTR, ITGB3, SNAP23, CLTC, DOCK7, ILK, RND3, PPP1CC, GJA1, CAPN5, DAG1, PGM5, GIT1, JAK1, DSP, PDGFRB, PPP1R12A, ACTN1, SHROOM3, ADAM10, YWHAZ, MTSS1, RHOB, ENAH, CAT, CDH13, ADAM9, MYH9, ITGA6, ITGA5, LCP1, ARHGEF2, SSX2IP, CD44, VCL, RALA, AHNAK, STX16, TWF1, LPP, CNN3, GNA13, EPB41L5, NCSTN, FLRT2, EPB41L2, LMLN, YES1, HSPA5, LIMK1, SPRY4, ARPC5, IGF2R, PLEKHA7, SMAD7, RAB10, TJP1, MARCKS, ACTC1, FAP, ABI2, RPS29, DLG5, KRAS, NF2, FGFR3

Table S3: Most significant Gene Ontology terms (Cellular Component) associated with miRNA targeted genes for BRCA.

Cellular Component	Count	Genes
GO:0005911 cell-cell junction	93	ITGB1, OXTR, B4GALT1, SH3KBP1, CTNND1, AQP4, PTPRK, CLDN1, SLC8A1, MYLK, AMOT, TMEM47, PNN, GJA1, DAG1, AKT1, SCN5A, SPTAN1, NCK1, SCN1A, MAGI1, TRPC4, ACTN1, HEG1, PRKCD, SHROOM2, FRS2, FRMD4A, RPGRIP1L, ACTN4, ANK3, ATP1B1, RHOA, TGFBR1, CD2AP, TIAM1, FRMD6, TRAF4, LCK, CLDN8, KIT, CLDN12, RAPGEF2, MYH9, ITGA6, PKP4, DSG4, STEAP1, DSC2, VCL, DSC3, COL17A1, CAMK2D, TWF1, AKAP6, KCNA5, PIK3R1, ADD3, LIN7C, MLLT4, MTDH, CDC42, DPP4, PARD6B, PAK1, PDZD2, UBN1, NHS, JAM2, KCNJ2, KIRREL, FZD5, PCDH9, FZD4, CGN, MPP5, CDC42BPA, INADL, MPP7, TJP1, MYO1E, AHI1, DLG3, VAPA, APC, DLG5, WNK3, FAT1, PKN2, SCN2A, FGF13, PVRL1, LAT
GO:0070161 anchoring junction	105	SCARB2, NCKAP1, OXTR, CTNND1, ARPC5L, TNC, PPP1CB, GJA1, ALCAM, DAG1, TNS3, TNS1, PDGFRB, MAP2K1, ACTN1, ADAM10, ACTN4, HSPG2, PKP4, DSC2, VCL, DSC3, PXN, TWF1, LPP, EFNB2, DPP4, EPB41L5, FLRT2, TSPAN9, PCBP2, TSPAN4, RSU1, FZD1, ZNF384, CAV1, RDX, MSN, PARVA, ARPC5, AHI1, PPFIBP1, PTPRC, DLG5, GNB2, WNK3, ITGA11, FAT1, FERMT2, ITGB1, YWHAE, NRP1, ARF1, B4GALT1, YWHAB, CLTC, LIMD1, RND3, TRIOBP, RND1, LASP1, YWHAQ, ITGAV, RAC1, SPTAN1, YWHAG, PPP1R12A, DST, MME, ITGA2, ANXA5, SHROOM2, RRAS2, REXO2, RHOA, MTSS1, SENP1, ENAH, MMP14, PPAP2B, NUMB, MYH9, ITGA6, DSG4, ARHGEF7, RALA, STX16, SYNPO2, MLLT4, CDC42, GNA13, CD59, GIT2, SVIL, SPRY4, MPP7, RAB10, TJP1, MYO1E, APC
GO:0005912 adherens junction	101	SCARB2, NCKAP1, OXTR, CTNND1, ARPC5L, TNC, PPP1CB, GJA1, ALCAM, DAG1, TNS3, TNS1, PDGFRB, MAP2K1, ACTN1, ADAM10, ACTN4, HSPG2, DSC2, VCL, PXN, TWF1, LPP, EFNB2, DPP4, EPB41L5, FLRT2, TSPAN9, PCBP2, TSPAN4, RSU1, FZD1, ZNF384, CAV1, RDX, MSN, PARVA, ARPC5, AHI1, PPFIBP1, PTPRC, DLG5, GNB2, WNK3, ITGA11, FAT1, FERMT2, ITGB1, YWHAE, NRP1, ARF1, YWHAB, CLTC, LIMD1, RND3, TRIOBP, RND1, LASP1, YWHAQ, ITGAV, RAC1, SPTAN1, YWHAG, PPP1R12A, DST, MME, ITGA2, ANXA5, SHROOM2, RRAS2, REXO2, RHOA, MTSS1, SENP1, ENAH, MMP14, PPAP2B, NUMB, MYH9, ITGA6, ARHGEF7, RALA, STX16, SYNPO2, MLLT4, CDC42, GNA13, CD59, GIT2, SVIL, SPRY4, MPP7, RAB10, TJP1, MYO1E, APC
GO:0044420 extracellular matrix part	44	COL17A1, ITGB1, LAD1, COL15A1, SPARC, LAMA2, COL11A1, ELN, COL12A1, TNC, SLC1A3, LAMC2, LAMC1, NID1, NID2, ADAMTS10, THSD4, DAG1, HMCN1, DST, HSPG2, RUNX1, VEGFA, COL1A1, ERBB2IP, COL3A1, COL2A1, COL1A2, FRAS1, COL5A1, COL4A2, COL4A1, COL5A3, COL7A1, COL4A4, MFAP2, COL5A2, COL4A3, VWC2, COL4A6, COL4A5, ITGA6, FREM2, FBN1
GO:0005667 transcription factor complex	71	SRA1, AHR, RBPJ, HOXA11, YY1, HOXA10, DACH1, EP300, PKNOX1, PITX2, SOX4, TEAD3, LMO4, NCOA6, TCF12, TFEB, RFX3, ARNT, FOS, POU3F1, POU3F2, RUNX2, NPAS4, RBL2, CREB1, TFDP1, HAND2, MYOD1, SUB1, TBPL1, CLOCK, YAP1, GTF2A2, BEX1, PRKDC, EPAS1, SATB2, GATA6, CREM, HIF1A, HDAC9, TAF5L, ARNTL, NHLH2, CREG1, E2F3, MSX1, TAF9B, E2F7, SMAD2, IVNS1ABP, JUN, ZFHX3, CREBBP, TAF12, NFYA, NFYB, MGA, TAF11, KLF4, SMAD6, SMAD5, NFATC4, SKI, MEIS1, MAFB, CDK2, TAF4B, TAF5, LPIN1, TAF2

Table S3: Most significant Gene Ontology terms (Cellular Component) associated with miRNA targeted genes for KIRC.

Cellular Component	Count	Genes
GO:0005829 cytosol	503	PHAX, BACH1, MYLK, LIPE, PSMD7, NAMPT, AP1S2, AP1S1, AP1S3, PRKACA, MEF2C, PRKAB2, DAXX, DICER1, FNDC3A, GAPVD1, GTPBP1, NISCH, BCR, DYNC1LI2, MTAP, DAAM1, PRKAR1A, MAP1B, PSME3, MAP1A, TXNIP, NUP98, VPS25, CLOCK, MTMR1, MTMR2, MTMR3, HSPA4L, MTMR4, HIF1A, EPB41L1, PRKAR2A, RAD21, MYO6, PPARGC1A, STARD13, JUP, DMPK, NFATC3, MTHFR, NFATC4, CYLD, QARS, GIPC1, FASN, FERMT2, ARF4, ITK, GABRB2, CDKN1A, CDKN1B, MTMR14, CLTC, PRKAG2, HIF3A, ARHGAP5, PFAS, CLINT1, LYST, SLC6A4, BBC3, EEA1, MECP2, IKBKB, TUBA1A, TNKS1BP1, JAK2, IKBKE, UPF2, CNOT6L, ITPK1, BAAT, SERPINB8, DYNLL2, POU5F1, COPZ1, PGM2L1, PELI1, EXOC5, PLCB1, ACSBG2, PAFAH1B1, PELI2, RNF135, SRP72, MFN2, DMD, RICTOR, FARP1, EGLN2, USP9X, STAT3, WWP1, BBOX1
GO:0043235 receptor complex	81	GABRB3, RET, APP, GABRB2, NRP1, NRP2, CD40, FLT1, CSF1, IRS1, ITGB3, GPR63, LOXL4, GRIK2, CD3E, GRM1, IGF1R, GHR, IKBKB, GRM7, LEPR, ITGB8, OLR1, IL6R, IL13RA1, ACVR1, CHUK, SACM1L, ITGA2, OSMR, ADRA2A, GABRG2, TGFBR1, TGFBR2, KCTD8, CD200R1, ADRB3, KCTD12, ITGA5, TLR4, SHANK2, CARD11, ITGA9, NOTCH2, CSF1R, NLGN1, CHRNA5, RNMT, HTRA2, LRP2, ADRB2, LRP8, LRP6, CD79B, SDCBP, GRIN2A, ACVR1C, ERBB3, ERBB4, CHRNE, PLXNA2, SLITRK5, STXBP5, IL12RB1, RFFL, LDLR, CACNG4, NTRK2, GABBR2, SMAD3, PTPRN2, NTRK3, INSR, NETO2, LRP1B, GRIN1, IL6, ITGA10, ITGA11, CNIH2, FGFR1
GO:0005654 nucleoplasm	230	RB1, HNRNPU, PHAX, HNRNPR, RBPJ, RPS6KA3, RPS6KA5, PSMD7, CDC23, LSM11, CHEK1, DAG1, TP63, RNF111, SKP1, TSPYL2, MEF2A, MED1, MEF2C, PRKAB2, ESCO1, DDX11, TINF2, MED8, MED6, PSME3, NUP98, ZFPM2, TP53, ASF1A, ATF3, EPAS1, GATA2, NLK, HIF1A, PCBP1, RAD21, MYO6, PCBP2, PPARGC1A, PPARGC1B, NR2C2AP, PHC2, H3F3A, NFATC3, NR1D2, SMARCA2, HNRNPL, STAG2, HNRNPK, RCOR1, EZH2, CDKN1A, RARG, CDKN1B, SETD8, ELL, PHF20, PRKAG2, HIF3A, PHF6, PHF8, COIL, PPP3CA, PPP3CB, MED13, ING3, HEY2, TEAD1, TEAD3, NCOA1, DUSP4, AEN, KIF23, FOS, MED26, SIRT1, POU5F1, SREBF2, NCOR2, KAT2A, RARB, PPARA, YAP1, ZNF473, RNMT, SATB1, RELA, RXRA, SUZ12, EGLN2, BRF2, NFYB, STAT3, RPA2, GTF2H1, APTX, SKI, RYBP, RFWD2
GO:0005667 transcription factor complex	80	RB1, RARG, RBPJ, ETS1, HOXA10, SOX2, TAF7L, DACH1, EP300, SOX8, PITX2, TP63, SOX4, TEAD3, SOX5, TBP, TCF12, TFEB, ARNT, FOS, POU3F2, POU5F1, RUNX2, NPAS4, KAT2A, TFDP2, TBPL1, TP53, CLOCK, YAP1, EPAS1, SATB2, CREM, HIF1A, HDAC9, NPAS2, RELA, TAF5L, E2F1, E2F2, E2F3, ALX1, E2F5, TAF9B, MTA2, E2F6, E2F7, SMAD2, GTF2A1L, IVNS1ABP, SMAD1, ZFHX3, SMAD3, TFAP2D, JUP, TAF12, FOXF1, NFYB, MGA, HMGA1, PBX3, PBX2, GTF2H1, TBX5, KLF4, SMAD5, POU2F3, USF1, SMAD7, TBX2, NFATC4, SKI, MEIS1, MAFB, CDK2, TAF4B, TCF3, TAF5, LPIN1, TAF4
GO:0000785 chromatin	80	RB1, AHCTF1, RARG, THRB, SETD3, SPI1, ELL, PPP1R10, PSIP1, CHD4, AFF4, MECP2, FAM178A, CCND2, SALL1, CHAF1A, CHEK1, POGZ, DPF2, SMARCAD1, WAPAL, TNKS1BP1, GABPA, TP63, NCOA1, MEF2A, MED1, SMARCC1, DAXX, MEF2C, TBP, H2AFY, ESCO1, EED, USP3, DDX11, TCF12, SIRT1, RUNX2, NCOR2, MAF, MYCN, WDR82, PLCB1, TP53, ASF1A, DNMT1, SMARCD2, CEBPB, UHRF2, SATB1, UHRF1, SRF, HTRA2, BAZ2A, PDS5A, RNF2, SCRT2, RXRA, MTA2, SMAD2, SUZ12, PHC2, SMAD3, UBE2B, MBD1, CBX2, HMGA1, HMGA2, KLF4, APTX, ESR1, SMARCA2, GATAD2B, SMARCA4, STAG2, HNRNPK, POLR3D, TCF3, EZH2

Table S3: Most significant Gene Ontology terms (Cellular Component) associated with miRNA targeted genes for LGG.

Cellular Component	Count	Genes
GO:0005829 cytosol	372	APP, WIPF1, WIPF2, ITSN1, DIXDC1, PPP2R2A, GBA3, RPS6KA3, GOLGA3, PREX1, GJA1, CDC23, OPHN1, DPYSL4, DPYSL5, PSMD5, RPS6KA2, DPYSL2, RPS6KA1, HABP4, SNRPD3, BTRC, PRKACB, SULT2A1, PRKCH, PRKCB, PRKCE, ACTN1, ANK2, FNDC3A, ANK1, WDR77, TIAM1, DYNC1LI2, DAAM1, XRN1, PSME3, AKAP9, MAP1A, PSME1, TXNIP, PRKCQ, ULK1, PRKD1, CLOCK, SEH1L, MTMR3, SAR1B, EPAS1, EIF5A2, NEDD4L, PIK3R3, VPS26A, PIK3R1, NLK, MTMR4, HIF1A, MTMR6, ABR, PPP3R1, LDHA, RIC8B, RHOT1, RACGAP1, PRKAR2A, CEP70, PIP5K1A, RSU1, PLCG1, RALGDS, ATP6V1C1, SPTBN1, SPTBN2, VASP, STARD13, YES1, UPF3A, PARVA, NFATC4, FOSL1, PEX5L, RPS29, CD28, FBXL3, RGL1, CEP57, GABRB2, CDKN1A, CDKN1B, ARPC1A, ARHGAP1, WASL, LYST, EEA1, MECP2, PPP3CA, AKAP13, ACTR1A, XPO5, TNKS1BP1
GO:0044456 synapse part	81	GABRB3, CHRM2, GABRB2, SNAP25, PTEN, SLC40A1, SYNE1, GRM3, RIMS4, PCLO, RIMS3, GRM7, OPHN1, PSD3, DAG1, SYNPO, BSN, DLGAP2, EPHA4, UNC13C, EPHA7, SVOP, SLC30A3, SLC6A17, GRID1, MAGI2, F2R, MINK1, ANK2, ANK3, AXIN2, SYN2, ANK1, YWHAZ, PJA2, DNM2, DNM3, TANC1, LPHN1, LRRC7, IL1RAPL1, SHANK3, STX1A, SHANK2, VAMP2, KCTD16, PFN2, CALCA, LRP4, LRP8, LIN7B, GLRA3, SV2B, CAMK2N1, VTI1A, SPOCK1, DMD, SLC17A7, PDLIM5, GABRE, SRGAP2, GABBR2, NTRK2, SYT4, SYT1, CADM1, BDNF, GABRA4, SEMA4C, AP3D1, CRIPT, SEMA4F, NETO2, SYT9, MIB1, FOSL1, CPEB1, GRIN3A, P2RX4, ABI1, CDK5R1
GO:0005794 Golgi apparatus	145	APP, TMEM167B, UXS1, SYAP1, CASC4, GCC2, CXCL14, IPO5, RAB43, GOLGA3, FGF7, LAPTM4A, GJA1, AKT3, GOLGA1, KDR, PTGFRN, SLC30A7, SH3GLB1, EPHA4, MAP2K1, PRKCE, GLCE, F2R, MTUS1, ANK3, FNDC3A, TMEM130, WDR77, SCAMP2, GOLGA8B, RNF128, AKAP9, PRKD2, ABCG1, MACF1, TMED10, SAR1A, LMAN2L, TNKS, SAR1B, GLIS3, ARL1, ZC3HAV1, GABARAP, APH1A, TGOLN2, VCPIP1, FUT8, STC2, VTI1A, RAB11FIP5, ST3GAL3, ABCA1, TFAP2A, B3GALNT1, YES1, FZD5, RHBDD2, SURF4, HOOK3, ESR1, KLHL20, BMP1, ACBD3, CEP57, CNTNAP2, MCFD2, HIP1, USP32, USP33, PDE3B, RND3, PKD1, BICD2, SYNE1, SART1, AP1G1, B3GALT2, TMED2, TEAD1, SH3D19, MINK1, ASH1L, MANEAL, IL17RD, ARFGAP3, ZDHHC17, CBFA2T3, PJA2, ARFGAP2, DNM2, GORASP2, IMPAD1, CSDE1, TCP1, ST6GALNAC3, MAPRE1, ST6GALNAC6, CANT1
GO:0045202 synapse	56	GABRB3, APP, GABRB2, ITSN1, CTNND1, PPP1R9A, SCAMP5, PCLO, CALB1, GRM7, BSN, SNAP29, EPHA4, EPHA7, MAGI2, F2R, MYRIP, ANK3, SYN2, CACNB4, LPHN1, RAPGEF2, MYH9, MGLL, VAMP2, HDAC4, CAMK2D, NRN1, LRP4, ATP1A2, CPLX2, ZNRF1, EFNB1, EGFLAM, SPOCK1, APBB2, DMD, SLC17A7, WASF1, LGI1, NTRK2, UBE2I, DTNA, SYT1, CADM2, SEMA4B, GRIN3A, RPS6KB1, RIMBP2, CNTN2, CBLN4, CPEB3, PVRL1, SPG20, CDK5R1, SNTB2
GO:0005654 nucleoplasm	167	RB1, CCNT2, RORC, UBE2D1, JMJD1C, PPP2R2A, ADARB1, ETS1, RPS6KA3, RPS6KA5, TATDN2, CDC23, CCND1, PSMD5, RPS6KA2, MYC, SCMH1, RPS6KA1, PAPOLA, DAG1, EP300, KPNA4, SNRPD3, KPNA2, KPNA3, PRKACB, KPNA1, MAP2K3, TLE4, MED1, DYRK1A, CDC40, CDC25A, NRBP1, MED7, MORF4L1, CCNE2, TBL1XR1, PSME3, PSME1, ZFPM2, SQSTM1, ASF1A, NOTCH2, NOTCH3, NOTCH1, EPAS1, GATA6, CUL1, NEDD4L, GATA3, PDS5A, NLK, HIF1A, NEUROD1, NPAT, PPP3R1, ATXN1, UBC, NRBF2, NKX2-2, SMAD2, ESRRA, SMAD1, UBE2I, HMGA2, ESRRG, SMAD5, ESR1, SMAD7, NR4A2, HNRNPL, HNRNPK, NR4A3, MYF5, CDKN1A, RARG, CDKN1B, THRA, CHD9, HNF4G, ADAR, PHF6, MED17, COIL, PTBP1, PPP3CA, SART1, CASP7, MED13, SIN3A, HNF4A, XPO5, TEAD1, TEAD3, NCOA1, PPP1R12A, TGIF2, NCOA6, MSL2

Table S3: Most significant Gene Ontology terms (Cellular Component) associated with miRNA targeted genes for LIHC.

Cellular Component	Count	Genes
GO:0044456 synapse part	113	CHRM3, RIMS1, SYDE1, RIMS4, RIMS3, DAG1, PPFIA3, ANKS1B, EPHA4, UNC13C, EPHA7, UNC13A, MAGI2, ADAM10, ANK2, SYPL2, ANK1, SYTL2, KCTD8, SYPL1, KCTD12, UTRN, STX1A, PFN2, SHC4, ZC4H2, ITPR1, FBXO45, CACNA1C, LIN7C, SYNGR1, KIF3A, VTI1A, DRD2, GRIA3, NGFR, SYT4, SYT3, FZD3, TOR1A, SYT1, CADM1, BDNF, HOMER2, PTCH1, NETO2, SYT7, SYT6, FOSL1, CPEB1, DLG2, GRIN3A, DLG4, GIPC1, STRN, CNIH2, RAB5A, CPEB4, GABRB3, SNAP25, PTEN, ICA1, GRIK2, GRM1, GPHN, SYNE1, TRIM9, PCLO, GRM7, LRRTM3, PSD3, ERC2, SLC18A2, MINK1, AXIN2, SYN2, YWHAZ, DNM3, PLCB4, LPHN1, ALS2, LRRC7, DNAJC5, IL1RAPL1, SHANK3, NLGN1, NLGN2, NRXN1, LRP4, ADD1, GLRA2, CAMK2N1, CHRNE, SLC17A6, SPOCK1, DMD, PDLIM5, GABRE, SNCB, GABBR2
GO:0045202 synapse	79	GABRB3, APP, CHRM3, SH3KBP1, KCNC3, FMR1, ITSN1, SNAP23, KCNC4, PPP1R9A, GPHN, SCAMP5, PCLO, GRM7, NRCAM, DLGAP4, EPHA4, EPHA7, UNC13A, MAGI2, NRG1, MYRIP, SYN2, ENAH, OLFM1, LPHN1, VAMP7, SLC9A6, PRKAR1A, RAPGEF2, COL4A5, VAMP1, UTRN, MGLL, HDAC4, USP14, NLGN1, NRN1, NLGN2, CTBP2, LRP4, CPLX2, MFF, CPLX4, LRP6, ZNRF1, EFNB1, ZNRF2, DVL1, SPOCK1, APBB2, DMD, STXBP5, WASF1, MYH10, FARP1, GABRP, NTRK2, UBE2I, DTNA, SYT1, CADPS, SAMD4A, NMNAT2, GRIN1, GRIN3A, DLG3, RPS6KB1, DLG4, RIMBP2, CTNNB1, IGSF9B, CBLN4, CPEB3, FGFR2, PVRL1, BCL2L1, CDK5R1, SNTB2
GO:0005829 cytosol	499	NCKAP1, PI4K2B, TRIO, WIPF1, ITSN1, BACH1, SMC2, MYLK, LIPE, PSMD7, DPYSL2, DPYSL3, NAMPT, PSMD3, AP1S2, AP1S1, PIP4K2B, AP1S3, PRKACB, PRKAB2, ACOT7, CSNK2A1, PRKCB, PRKCE, PRKCA, DICER1, FNDC3A, EML1, NISCH, BCR, DYNC1LI2, PRKAR1A, PSME3, MAP1A, NUP98, PRKD1, CLOCK, TPH2, SEH1L, MTMR3, HSPA4L, VPS26B, MTMR4, OAZ2, PPP3R1, RIC8B, EPB41L1, PRKAR2A, PIP5K1A, PPARGC1A, SERF2, NGFR, STARD13, JUN, CHKA, RAB3IP, NFATC3, UPF3B, MTHFR, FOSL1, CYLD, PPP5C, EIF5, QARS, GIPC1, FASN, NMT1, HNRNPD, NMT2, FERMT2, ITK, SMG1, CDKN1A, CLTC, HIF3A, SMG7, ARHGAP5, ARHGAP6, LYST, SMG5, GLI3, SLC6A4, BBC3, EEA1, MECP2, IKBKB, TUBA1A, IKBKE, APPL1, UPF2, LYPLA1, CNOT6L, DUSP3, ITPK1, DYNLL1, DYNLL2, PGM2L1, PLCB4, EXOC5, CEP63
GO:1903293 phosphatase complex	23	PPP1R15B, PPP1R12A, SMEK1, ITPR1, PPP2R2A, PPP2R3A, PPP2R5C, NKD1, STRN3, PPP1CA, PPP2CA, TOX4, PPP1CC, PPP3CA, PPP3R1, PPP3CB, PPP2R2C, PPP2R1A, WDR82, PPP2R2B, PPP2R5E, SHOC2, STRN
GO:0008287 protein serine/threonine phosphatase complex	23	PPP1R15B, PPP1R12A, SMEK1, ITPR1, PPP2R2A, PPP2R3A, PPP2R5C, NKD1, STRN3, PPP1CA, TOX4, PPP2CA, PPP3CA, PPP1CC, PPP3R1, PPP3CB, PPP2R2C, PPP2R1A, WDR82, PPP2R2B, PPP2R5E, SHOC2, STRN

Table S3: Most significant Gene Ontology terms (Cellular Component) associated with miRNA targeted genes for LUAD.

Cellular Component	Count	Genes
GO:0005829 cytosol	402	NCKAP1, WIPF2, PHAX, SMC3, MYLK, LIPE, GJA1, PSMD7, DPYSL2, AP1S2, AP1S3, PRKACA, PRKACB, PRKAB2, PRKCE, DICER1, NISCH, BCR, DYNC1LI2, NGFRAP1, PSME3, MAP1A, PSME4, TXNIP, HPRT1, CLOCK, MTMR1, MTMR3, HSPA4L, MTMR4, HIF1A, PRKCZ, PPP3R1, EPB41L1, PRKAR2A, PLCG1, PPARGC1A, STARD13, NFATC3, MTHFR, LNPEP, NFATC4, FOSL1, CYLD, FASN, HNRNPD, FERMT2, ARF4, GABRB2, CDKN1A, CLTC, ARHGAP1, ARHGAP5, LYST, SLC6A4, MECP2, IKBKB, TUBA1A, NEFL, APPL1, CNOT6L, SPHK2, PGAM1, ITPK1, BAAT, DYNLL2, PGM2L1, ITPKB, PELI1, PPARG, EXOC5, PLCB1, ACSBG2, RAPGEF4, PAFAH1B1, PELI2, SRP72, MFN2, DMD, RICTOR, EGLN3, EGLN2, USP9X, WWP1, TARBP2, RFWD2, ETNK1, UBAP1, CENPO, IRS1, UBE2D2, UBE2D1, IRS2, MYLK3, NUDT10, FNTA, GRB10, KPNA4, MAP3K7, KPNA3
GO:0044456 synapse part	89	CHRM2, GABRB2, SNAP25, LRRK2, LRRC4, PTEN, ICA1, GRM1, GRM3, EPS8, SYNPR, TRIM9, SYDE1, GRM5, PCLO, RIMS3, GRM7, LRRTM3, LRRTM1, PSD3, LRRTM2, DAG1, NPTN, ERC2, DLGAP2, PPFIA2, NSF, EPHA4, EPHA7, UNC13A, GRID1, MINK1, ANK2, AXIN2, SYN2, ANK1, YWHAZ, SYTL2, DNM2, DNM3, KCTD8, TANC1, LPHN1, SYPL1, SNPH, LRRC7, DNAJC5, IL1RAPL1, SHANK3, STX1A, SHANK2, PFN2, NLGN1, CHRNA5, ITPR1, LRP4, LRP8, CHRNE, VTI1A, SPOCK1, DMD, PDLIM5, GABRE, SNCA, GABBR2, GABRP, NTRK2, SYT4, GABRA1, SYT3, FZD3, HOMER1, SYT1, CADM1, BDNF, PTCH1, NETO2, MIB1, SYT6, GRIN1, FOSL1, CPEB1, PPFIBP1, DLG2, AAK1, CNIH2, RAB5A, CDK5R1, CPEB4
GO:0030426 growth cone	35	GPM6A, NRP1, SNAP25, NRSN1, TSHZ3, AATK, PSEN1, IQGAP1, PPP1R9A, PAK1, LRRTM1, DPYSL2, DVL1, TIMP2, NEFL, ERC2, MYH10, SNCA, EPHA4, NTRK2, PCDH9, USP9X, AMFR, PTCH1, MYO5A, TSC1, DICER1, LPHN1, STIM1, APC, RUFY3, SHANK2, PAFAH1B1, CDK5R1, MAP3K12
GO:0030424 axon	47	APP, CNTNAP2, NRP1, NRP2, SEMA3A, LRRK2, PSEN1, IQGAP1, PTPRK, GRM3, SYDE1, PAK1, GRM7, CA2, LRRTM1, DPYSL2, DVL1, SACS, NEFL, MYH10, SNCA, EPHA4, FZD3, UNC13A, DTNA, HOMER1, DST, CADM1, IRX3, DAB2IP, DICER1, BACE1, GAP43, LPHN1, NOV, NFIB, CANX, TMEM57, NF1, CNTN4, SCN2A, RAB5A, PVRL1, TNFRSF21, HCN1, CDK5R1, MAP3K12
GO:0030427 site of polarized growth	35	GPM6A, SNAP25, NRP1, NRSN1, TSHZ3, AATK, PSEN1, IQGAP1, PPP1R9A, PAK1, LRRTM1, DPYSL2, DVL1, TIMP2, NEFL, ERC2, MYH10, SNCA, EPHA4, NTRK2, PCDH9, USP9X, AMFR, PTCH1, MYO5A, TSC1, DICER1, LPHN1, STIM1, APC, RUFY3, SHANK2, PAFAH1B1, CDK5R1, MAP3K12

Table S3: Most significant Gene Ontology terms (Cellular Component) associated with miRNA targeted genes for PAAD.

Cellular Component	Count	Genes
GO:0044456 synapse part	67	GABRB3, SNAP25, ARF1, CHRM1, LRRC4, SYDE1, RIMS4, PCLO, RIMS3, OPHN1, LRRTM3, PSD3, LRRTM2, DLGAP1, DLGAP3, BSN, ERC2, DLGAP2, PPFIA4, EPHA4, UNC13C, GRID1, MAGI2, MINK1, CASK, ANK2, ANK3, ANK1, AMPH, SHANK3, STX1A, PFN2, HCN4, SHC4, BRSK1, NLGN2, NRXN1, CACNA1C, CABP1, LIN7C, LRP8, GLRA2, SV2A, CAMK2N1, FYN, PDLIM5, SRGAP2, GRIA4, GABRP, NGFR, NTRK2, SYT4, GABRA1, SYT2, NLGN4X, SYT1, CADM1, BDNF, PTCH1, SEMA4C, SEMA4F, CCK, GRIN1, GRIN3A, GHRH, CDK5R1, CPEB4
GO:0030425 dendrite	43	CNTNAP2, BMPR2, CHRM1, MAX, HTT, CABP1, LRP8, TXN2, RELN, OPA1, CAMK2N1, IGF2BP1, STX3, EPHB2, BSN, SOX6, TP63, ZNF385A, GRIA4, EPHA4, NLGN4X, CADM1, GRID1, PLK2, MAGI2, KCNJ14, RAB27A, CCK, ANK3, MAPK8IP3, ZWINT, GRIN1, GRIN3A, CCNG1, RARA, NF1, AQP11, ATXN10, FGF13, CPEB3, CDK5R1, CPEB4, BMPR1A
GO:0097060 synaptic membrane	41	SHC4, GABRB3, SNAP25, ARF1, NLGN2, CHRM1, NRXN1, LRRC4, CABP1, LIN7C, SYDE1, RIMS4, GLRA2, LRRTM3, CAMK2N1, PSD3, LRRTM2, DLGAP1, DLGAP3, PDLIM5, SRGAP2, ERC2, DLGAP2, GRIA4, GABRP, NTRK2, EPHA4, GABRA1, NLGN4X, SYT1, GRID1, SEMA4C, MINK1, CASK, SEMA4F, ANK2, ANK3, ANK1, GRIN1, GRIN3A, SHANK3
GO:0045202 synapse	43	GABRB3, CAMK2D, NLGN2, CHRM1, KCNC3, CAMK2A, CTNND1, LRRC4, ATP1A2, CPLX3, SCAMP5, PCLO, SV2A, LRRTM2, PRKACA, BSN, MYH10, PPFIA4, GRIA4, GABRP, EPHA4, NTRK2, DTNA, NLGN4X, SYT1, CADM2, MAGI2, SAMD4A, NMNAT2, ANK3, GRIN1, ENAH, OLFM1, GRIN3A, DLG3, RPS6KB1, RAPGEF2, ITGA5, TLN2, AGRN, CPEB3, BCL2L1, CDK5R1
GO:0005911 cell-cell junction	52	CTNND1, AQP4, SLC8A1, AMOT, CDH5, CGNL1, SCN1A, MAGI1, CD53, MAGI3, HEG1, MAGI2, PRKCD, KIAA1462, CASK, FRS2, ANK2, FRMD4A, RPGRIP1L, ANK3, TGFBR1, TIAM1, RAPGEF2, CLDN18, ITGA5, TLN2, ARHGEF6, CAMK2D, WAS, LIN7C, MTDH, CDC42, RAP1B, PARD6B, SV2A, NHS, CDH24, STX3, FZD5, CADM1, FZD4, CDC42BPB, NFASC, VANGL2, DLG3, VAPA, APC, PKN2, GRB2, AMOTL2, AMOTL1, FGF13

Table S3: Most significant Gene Ontology terms (Cellular Component) associated with miRNA targeted genes for PRAD.

Cellular Component	Count	Genes
GO:0005911 cell-cell junction	92	ITK, PTPRU, CTNND1, AQP4, PTPRK, CLDN1, SLC8A1, MYLK, CALB2, TMEM47, PNN, CDH5, PANX2, GJA1, CCND1, DAG1, SCN5A, CGNL1, NCK1, SCN1A, MAGI1, DSP, ACTR3, MAGI3, ACTN1, HEG1, MAGI2, PRKCD, FRS2, ANK2, FRMD4A, RPGRIP1L, ANK3, ASH1L, RHOA, TGFBR1, CD2AP, CLDN11, FRMD6, CLDN12, RAPGEF2, MYH9, ITGA6, ITGA5, SCN4B, SSX2IP, ARHGEF6, CAMK2D, AHNAK, COL13A1, TWF1, AKAP6, PIK3R1, ADD3, LIN7C, MLLT4, MTDH, CDC42, RAP1B, GJC1, PARD6B, PAK1, SV2A, UBN1, NHS, CDH24, PLCG1, STX3, STX2, FZD5, CADM1, PCDH9, KCNJ11, FZD4, CDC42BPB, MPP5, INADL, PLEKHA7, MYO1E, AHI1, NFASC, VANGL2, VAPA, APC, DLG5, WNK3, PKN2, GRB2, AMOTL2, AMOTL1, SCN2A, FGF13
GO:0005654 nucleoplasm	229	RB1, MAML1, JMJD1C, CCNC, RPS6KA3, RPS6KA5, PSMD7, LSM11, RPS6KA2, CDC27, DAG1, JUNB, RNF111, MEF2A, MEF2C, PRKAB2, ESCO1, PRKCD, REV1, MED8, MED6, AR, PSME3, NUP98, TP53, ATF3, GATA6, NEDD4L, GATA3, GATA2, NLK, HIF1A, PPP3R1, NRBF2, PPARGC1A, PPARGC1B, NKX2-2, NGFR, ZNF143, PHC2, H3F3A, SMARCA5, NR1D2, NR2F6, STAG1, STAG2, HNRNPF, HNRNPD, RAD17, HNRNPC, CALM1, RCOR1, EZH2, CDKN1A, RARG, ELL, PHF20, HNF4G, RSF1, HIF3A, CTCF, BMI1, PHF6, PHF8, COIL, ING5, PPP3CA, PPP3CB, MED14, XPO1, MED13, TEAD1, TEAD3, NCOA1, DUSP4, DUSP3, AEN, FOS, SIRT1, NCOR2, KAT2B, KAT2A, NR5A2, RNF169, PSMA1, RARA, RARB, PPARG, PPARA, RNMT, SATB1, RELA, RBM15B, RXRA, UBN1, TBL1X, SUZ12, EGLN2, BRF2, NFYB
GO:0000785 chromatin	81	RB1, SUV39H2, AHCTF1, RARG, THRB, SETD3, SPI1, ELL, PSIP1, CHD4, AFF4, MECP2, FAM178A, CCND2, SALL1, CHAF1A, ALKBH1, RUVBL2, POGZ, DPF2, SMARCAD1, WAPAL, JUNB, GABPA, ZNF385A, NCOA1, MEF2A, SMARCC1, MEF2C, ESCO1, H2AFZ, EED, ATRX, TCF12, DNMT3A, ARID1A, SIRT1, RUNX2, TOX4, NCOR2, AR, MAF, CREB1, MYCN, WDR82, RARA, TP53, SMARCD2, CEBPB, UHRF2, SATB1, BAZ2A, GATA3, PDS5B, PDS5A, SCRT2, RXRA, HLCS, SMAD2, SUZ12, PHC2, CBX6, UBE2B, CBX3, CBX2, CBX1, HMGA1, HMGA2, SMARCA1, GATAD2B, SMARCA4, ASXL1, STAG1, STAG2, POLR3D, NEDD4, ID2, SNAI2, HNRNPC, NFE2L2, EZH2
GO:0005829 cytosol	487	NCKAP1, PI4K2B, WIPF1, WIPF2, ITSN1, ABCA12, BACH1, MYLK, GJA1, PSMD7, DPYSL2, AKT2, STMN1, NAMPT, PIP4K2A, AP1S1, PIP4K2B, MEF2C, PRKAB2, PRKCD, DICER1, FNDC3A, EML1, DEPDC1B, NGFRAP1, PRKAR1A, MAP1B, PSME3, NUP98, CLOCK, SEH1L, MTMR3, NEDD4L, VPS26B, MTMR4, HIF1A, OAZ2, PPP3R1, HSPH1, HLCS, EPB41L1, PIP5K1B, RSU1, PLCG1, PPARGC1A, CEP76, LYN, NGFR, STARD13, CHKA, EIF5, QARS, GIPC1, HNRNPD, NMT2, PLCH1, HNRNPC, ARF4, ITK, GABRB2, ARF1, CDKN1A, CLTC, HIF3A, ARHGAP5, CLINT1, LYST, BBC3, EEA1, MECP2, NEFL, JAK2, IKBKE, JAK1, UPF2, LYPLA1, DUSP3, ITPK1, PGM2L1, PLCB4, PSMA1, PELI1, GAS2, PPARG, EXOC5, RAPGEF4, PAFAH1B1, AHNAK, URM1, PELI2, HTT, SNX33, TANK, RNF135, DMD, RICTOR, FARP1, EGLN2, CSNK1A1, USP9X
GO:0030425 dendrite	69	PCSK2, CNTNAP2, BMPR2, CHRM1, GRIK2, PTPRK, TXN2, TRIM9, OPA1, CHL1, DPYSL2, SACS, EPHB2, BSN, SOX6, ZNF385A, RAB8A, KPNA1, EPHA4, ARHGEF15, MAGI2, DICER1, ANK3, MAPK8IP3, ADCY9, PLCB4, SCN8A, IL1RAPL1, RARA, BECN1, GRIA2, NLGN1, STAU1, KCNA1, SEMA3A, HTT, SLC1A4, CABP1, CPLX2, DDN, STRN3, BEGAIN, PAK1, RELN, CAMK2N1, IGF2BP1, STX3, GRIA4, FARP1, FZD3, UBE2I, NLGN4X, CADM1, PLK2, RAB27A, ZWINT, PEX5L, GRIN3A, CCNG1, NF1, GNAS, AQP11, FAT3, ATXN10, FGF13, CPEB3, CDK5R1, CPEB4, BMPR1A

Table S3: Most significant Gene Ontology terms (Cellular Component) associated with miRNA targeted genes for SKCM.

Cellular Component	Count	Genes
GO:0044456 synapse part	56	GABRB3, GABRB2, LRRC4, ILK, GRM1, EPS8, SYDE1, RIMS4, PCLO, PSD3, GRM8, PPFIA1, DAG1, DLGAP2, ANKS1B, CADPS2, UNC13C, EPHA7, MAGI2, MINK1, ANK2, ANK3, SORCS3, YWHAZ, DNM3, KCTD8, LPHN1, ALS2, LRRC7, AMPH, KCTD12, SHANK3, SHANK2, VAMP2, MTMR2, NRXN1, FBXO45, CACNA1C, LIN7C, SLC17A6, SPOCK1, SRGAP2, SNCA, SYT1, CADM1, BDNF, HOMER2, PTCH1, PCDH8, TRPV1, PPFIBP1, DLG2, LRFN1, ABI1, CPEB4, PICALM
GO:0005654 nucleoplasm	112	RB1, CCNT2, HNRNPR, RORB, RBPJ, NR3C1, ETS1, ETS2, NR3C2, RPS6KA3, PSMD3, PAPOLA, DAG1, BANF1, KPNA4, KPNA3, PRKACB, JUNB, RNF111, KPNA1, MEF2C, DYRK1A, SRRM1, DDIT3, ZFPM2, KPNB1, SET, NRF1, PDS5B, PDS5A, NLK, GTF2E1, PRPF8, HIF1A, CDKN2AIP, PHF21A, NEUROD1, CCNB1, ATXN1, TP53BP1, PPARGC1A, STAT5B, PHC2, XBP1, CBX1, ESRRG, NUP153, ESR1, STAG1, STK24, SP1, ID1, HNRNPD, TAF4B, HNRNPC, SETD8, CHD9, CHD6, PSIP1, CHD4, CTCF, HMGB1, PHF8, PTBP1, MED14, NIPBL, MED13, ING3, SIN3A, WAPAL, TEAD1, NCOA2, PPP1R12A, DUSP3, NCOA6, MSL2, SMC1A, YWHAZ, SIRT1, RAD23B, MSL1, SFPQ, NR5A2, PAX8, HNRNPH1, HDAC5, RRN3, RNMT, SATB1, LEF1, HDAC9, RNF2, RBBP4, ERBB4, UBN1, USP1, E2F3, E2F5, CAMK2G, RBM39
GO:0097060 synaptic membrane	34	GABRB3, GABRB2, MTMR2, NRXN1, LRRC4, FBXO45, LIN7C, SYDE1, RIMS4, PSD3, GRM8, DAG1, SRGAP2, DLGAP2, ANKS1B, CADPS2, EPHA7, SYT1, HOMER2, MINK1, PCDH8, ANK2, TRPV1, ANK3, KCTD8, DLG2, LPHN1, LRRC7, LRFN1, ABI1, KCTD12, SHANK3, SHANK2, PICALM
GO:0005730 nucleolus	147	HNRNPR, CELSR1, RBPJ, NR3C1, FGF1, ETS1, CDC14A, SOX2, PAPOLG, CWC22, PSMD3, OSBP, PAPOLA, SMARCAD1, SOX9, JUNB, RNF111, SOX4, GTF2I, KPNA1, MAGI1, MBNL1, ZBTB33, FOXP1, SRRM1, HIC2, CIRH1A, DMTF1, HOXB7, DGKI, KPNB1, CGGBP1, KHDRBS1, ANKRD12, PXN, PDS5B, PDS5A, HIF1A, GTF2E1, CDKN2AIP, FXR1, LARP1, ATXN1, ATXN7, ZMAT5, UBR5, HIVEP2, RAB11FIP2, N4BP1, HEXIM1, PHC2, ACBD5, ZRANB2, HOMER2, BCL11A, MICAL3, NUP153, CLK2, PPP5C, UBLCP1, NASP, STK24, SP1, CNOT1, SP4, ID2, ID1, HNRNPD, TAF4B, FBXL3, HBP1, INTS6, HNRNPC, BAP1, FGFR2, PHF2, ZCCHC11, RBM25, SETD8, ILK, PSIP1, CTCF, AFF4, PHF8, SENP6, PTBP1, SPRED1, ING3, SIN3A, WAPAL, UBXN7, RBM7, YAF2, TEAD1, RRP15, NDEL1, NCOA6, PRPF40A, DDX52, SMC1A
GO:0045121 membrane raft	30	RET, ITGB1, APP, GSK3B, STX12, BMPR2, AKAP6, ATP1A2, GNAI1, IGF1R, LRP6, GPC1, DAG1, MAL2, JAK2, ABCA1, CAV1, INSR, PTCH1, ATP2B4, ANK2, SORBS1, ATP1B1, SULF1, CLIP3, ACE2, MYO1C, DLC1, KRAS, TEK

Table S3: Most significant Gene Ontology terms (Cellular Component) associated with miRNA targeted genes for STAD.

Cellular Component	Count	Genes
GO:0005829 cytosol	481	NCKAP1, TRIO, WIPF1, WIPF2, BACH1, MYLK, LIPE, PREX1, GJA1, PSMD7, DPYSL5, DPYSL2, NAMPT, AP1S2, AP1S3, PRKACA, PRKACB, SULT2A1, MEF2C, PRKAB2, ACOT7, DICER1, FNDC3A, NISCH, BCR, DYNC1LI2, MAP1B, PSME3, MAP1A, TXNIP, PRKD1, CLOCK, SKAP2, SEH1L, MTMR3, SAR1B, HSPA4L, NEDD4L, VPS26A, MTMR4, HIF1A, OAZ2, PPP3R1, RHOT1, HLCS, EPB41L1, PRKAR2A, CEP70, PPARGC1A, JUN, NFATC3, MTHFR, UPF3A, FOSL1, CYLD, EIF5, FASN, FERMT2, CEP57, ITK, GABRB2, ARF1, SMG1, CDKN1A, QPRT, CLTC, ARHGAP1, ARHGAP5, LYST, SLC6A4, EEA1, MECP2, IKBKB, TUBA1A, TNKS1BP1, LDLRAP1, JAK1, LYPLA1, CNOT6L, TPI1, PGM2L1, ITPKB, DPYD, TCP1, PELI1, RAPGEF1, EXOC5, CEP63, PLCB1, PAFAH1B2, RAPGEF4, PAFAH1B1, RNASEL, AHNAK, PELI2, SNX33, FGD1, FGD4, SRP72, CHN2
GO:0005794 Golgi apparatus	182	USP6NL, APP, ATP8A1, GOLT1B, UXS1, SYAP1, GOLIM4, CASC4, GCC2, CXCL14, IPO5, FGF7, LAPTM4A, GJA1, GOLGA4, GOLGA5, HERC1, AKT3, GOLGA1, KDR, FAM3C, PTGFRN, PSKH1, SLC30A7, EPHA4, GABARAPL1, MAP2K1, SLC30A8, FBXW7, SACM1L, F2R, CSNK1D, ANK3, FNDC3A, SYTL2, SCAMP2, GOLGA8B, RRAGA, RNF128, AKAP9, RAF1, RTN3, MACF1, LMAN2L, SAR1B, ARL3, GLIS3, ARL1, UMOD, PKMYT1, FUT4, TGOLN2, VCPIP1, FUT8, ATXN2, VTI1A, EVI5, RAB11FIP5, ABCA1, TFAP2A, YES1, ATP8B1, PTCH1, ST8SIA3, RAB27A, MYO5A, RAB39B, HOOK3, ESR1, KLHL20, EXT2, FASN, CALU, CPE, CSPG5, GRB2, GALNTL6, FGFR2, PICALM, CEP57, CNTNAP2, B4GALT1, USP32, USP33, LRRK2, GDI2, PDE3B, AP4E1, LITAF, RND3, PKD1, BICD2, SYNE1, SART1, AP1G1, B3GALT2, KIF1C, DNM1L, TEAD1, PRKG1
GO:0005911 cell-cell junction	83	APP, ITK, B4GALT1, PTPRM, AQP4, CD3E, CLDN2, PKD2, CLDN1, MYLK, AMOT, PNN, PANX2, GJA1, CCND1, POF1B, BAI1, BVES, NCK1, YWHAH, SCN1A, MAGI1, ITGA4, MAGI3, HEG1, MAGI2, KIAA1462, SHROOM3, FRS2, LMO7, ANK2, FRMD4A, RPGRIP1L, ANK3, ASH1L, ATP1B1, TGFBR1, CD2AP, TIAM1, FRMD6, CLDN12, MYADM, RAPGEF2, CLDN18, ITGA6, PKP4, PRKD1, DSG4, SCN4B, SSX2IP, ARHGEF6, CAMK2D, AHNAK, TWF1, PIK3R1, IQGAP1, LIN7B, MTDH, CDC42, PARD6B, NHS, KCNJ2, JAM3, VASP, CADM1, PCDH9, FZD4, MPP5, CDC42BPA, PLXDC1, SMAD7, MYO1E, AHI1, NFASC, WNK3, ZYX, FAT2, PKN2, GRB2, AMOTL1, SCN2A, PVRL2, PVRL1
GO:0005654 nucleoplasm	206	RB1, HNRNPU, PPP2R2A, ADARB1, RPS6KA3, RPS6KA5, PSMD7, CDC23, LSM11, RPS6KA2, CHEK1, RPS6KA1, CDC27, SNRPD3, PRKACB, RNF111, TSPYL2, MED1, MEF2C, PRKAB2, ESCO1, DDX11, CDC40, MED7, PSME3, ZFPM2, ASF1A, EPAS1, NEDD4L, NLK, HIF1A, PPP3R1, PPARGC1A, NKX2-2, STAT5A, ZNF143, JUN, NFATC3, CDKN1A, RARG, ELL, PHF20, HNF4G, BMI1, PHF6, MED17, COIL, PPP3CA, PPP3CB, MED13, HEY2, TEAD1, TEAD3, PAX4, KIF23, FOS, MED26, NCOR2, KAT2B, RAD51C, RNF169, RARB, PPARA, PPARD, YAP1, RNMT, PRIM1, HIRA, RBM15B, TBL1X, SUZ12, GIT2, EGLN3, EGLN2, STAT3, GTF2H1, SKI, PSMC6, RYBP, NR6A1, XPOT, RFWD2, WT1, CAMK4, ZNF217, HDGF, CENPO, CCNT2, FMR1, RORC, UBE2D1, NR3C1, CDC14B, NR3C2, CCND1, PAPOLA, EP300, KPNA4, KPNA2, KPNA3
GO:0044456 synapse part	92	CHRM2, GABRB2, SNAP25, ARF1, LRRK2, LRRC4, PTEN, SLC40A1, GRM1, SYNE1, TRIM9, SYDE1, RIMS3, GRM7, OPHN1, PSD3, ERC2, DLGAP2, PPFIA3, EPHA4, EPHA7, UNC13A, SLC30A3, MAGI2, F2R, MINK1, ANK2, ANK3, AXIN2, SYTL4, YWHAZ, PJA2, SYTL2, DNM2, KCTD8, ADCYAP1, TANC1, LPHN1, SYPL1, LRRC7, IL1RAPL1, KCTD12, UTRN, SHANK3, STX1A, SHANK2, PFN2, NLGN1, CHRNA5, NRXN1, ITPR1, CACNA1C, LIN7B, SV2B, CAMK2N1, KIF3A, CHRNE, VTI1A, SLC17A6, SLC17A7, PDLIM5, GABRE, SRGAP2, GRIA3, GABBR2, GABRP, NTRK2, SYT4, SYT3, HOMER1, CADM1, BDNF, GABRA4, PTCH1, AP3D1, CRIPT, NETO2, MIB1, GRIN1, FOSL1, DLG2, GRIN3A, COPS5, P2RX4, DLG4, SYT11, ABI1, GOPC, CNIH2, RAB5A, CDK5R1, PICALM

Table S3: Most significant Gene Ontology terms (Cellular Component) associated with miRNA targeted genes for THCA.
