# Supplementary material for: Genome-wide analysis of NGS data to compile cancer-specific panels of miRNA biomarkers
Source: PLoS One. 2018 Jul 26;13(7):e0200353. doi: 10.1371/journal.pone.0200353 (PMC6061989; doi:10.1371/journal.pone.0200353)
Supplement: S4 Table — Most significant Gene Ontology terms (Biological Process) associated with miRNA targeted genes. (PDF) [file pone.0200353.s004.pdf]

Biological Process	Count	Genes
GO:0045664 regulation of neuron differentiation	95	ROBO2, NRP1, BMPR2, EPO, DIXDC1, ILK, VLDLR, GLI3, GLI2, EDNRB, DPYSL2, CFL2, SIX3, DPYSL3, NEFL, NEFM, SOX9, EPHB2, RGS6, EPHB3, NDEL1, NCOA1, MED1, TCF12, MAGI2, OMG, COBL, SOX11, TBR1, PAX6, PROX1, ADRA2B, CIT, ISL2, CTDSP1, UBE2V2, RARB, RAPGEF2, ULK2, PRKD1, EPHA3, PAFAH1B1, RTN4R, KANK1, NUMBL, NLGN1, NOTCH1, SEMA3A, FOXG1, SHOX2, LRP4, GATA3, SEMA3F, ASCL1, ADCY6, NEUROD1, RAP1B, NEUROD2, CNR1, SPOCK1, ABL2, DMD, PACSIN1, NKX2-2, TFAP2A, LYN, SPAG9, SMAD1, NTRK2, ZFHX3, JAG1, SNAPIN, TMEM30A, NEGR1, BCL11B, BDNF, BCL11A, NTRK3, LIMK1, SSH2, RAB11A, MIB1, VEGFA, PTPRD, CCDC88A, BMP2, MEIS1, TLX3, NEDD4, BCL2, CNTN1, CNTN4, CPEB3, FOXA2, CDK5R1
GO:0097485 neuron projection guidance	83	ROBO2, NRP1, NRP2, TRIO, ITGB3, AP2A1, LAMC1, GLI3, GLI2, RPS6KA3, ZIC2, CHL1, RPS6KA2, DPYSL2, DPYSL3, NCK2, DAG1, HOXA1, EPHB2, EPHB1, SH3GL2, EPHB3, ARHGEF12, FOXD1, SEMA6A, UNC5A, CSNK2A1, SEMA6D, UNC5C, PAX6, ANK2, TBR1, UNC5D, RGMB, ANK1, RHOB, ENAH, CACNB2, ISL2, CREB1, SCN8A, DCX, MYH9, ITGA5, SEMA3A, MYCBP2, NRXN3, CACNA1C, GATA3, SEMA3F, EFNA4, FLRT2, FLRT3, PLXNA2, ST8SIA4, ABL1, PLXNA1, ABL2, MYH11, NCAM1, PLCG1, MYH10, LGI1, EGR2, FZD3, MYO10, BCL11B, BDNF, LIMK1, SIAH1, AP2B1, VAX1, POU4F2, VEGFA, EFNA3, DLG3, NFIB, ITGA10, CNTN1, KRAS, COL9A3, CNTN4, CDK5R1
GO:0007411 axon guidance	83	ROBO2, NRP1, NRP2, TRIO, ITGB3, AP2A1, LAMC1, GLI3, GLI2, RPS6KA3, ZIC2, CHL1, RPS6KA2, DPYSL2, DPYSL3, NCK2, DAG1, HOXA1, EPHB2, EPHB1, SH3GL2, EPHB3, ARHGEF12, FOXD1, SEMA6A, UNC5A, CSNK2A1, SEMA6D, UNC5C, PAX6, ANK2, TBR1, UNC5D, RGMB, ANK1, RHOB, ENAH, CACNB2, ISL2, CREB1, SCN8A, DCX, MYH9, ITGA5, SEMA3A, MYCBP2, NRXN3, CACNA1C, GATA3, SEMA3F, EFNA4, FLRT2, FLRT3, ST8SIA4, PLXNA2, ABL1, PLXNA1, ABL2, MYH11, NCAM1, PLCG1, MYH10, LGI1, EGR2, FZD3, BCL11B, MYO10, BDNF, LIMK1, SIAH1, AP2B1, VAX1, POU4F2, VEGFA, EFNA3, DLG3, NFIB, ITGA10, CNTN1, KRAS, COL9A3, CNTN4, CDK5R1
GO:0051962 positive regulation of nervous system development	74	ROBO2, NRP1, OXTR, BMPR2, EPO, DIXDC1, ILK, OTP, VLDLR, GLI3, GLI2, MECP2, GRM5, DPYSL3, NEFL, EPHB2, EPHB1, RGS6, EPHB3, NDEL1, NCOA1, SEMA6A, MAGI2, TCF12, COBL, SOX11, PAX6, PROX1, ADRA2B, PAX2, UBE2V2, RAPGEF2, RARB, PRKD1, EPHA3, PAFAH1B1, NUMBL, NLGN1, NOTCH1, PLAG1, SEMA3A, FOXG1, SHOX2, NRXN3, THBS2, ASCL1, NEUROD1, RAP1B, NEUROD2, CNR1, ABL2, DMD, NKX2-2, PACSIN1, SPEN, LYN, SPAG9, NTRK2, SMAD1, TMEM30A, NEGR1, BDNF, BCL11A, NTRK3, LIMK1, RAB11A, VEGFA, PTPRD, BMP2, CLCF1, BCL2, CNTN1, CPEB3, FOXA2
GO:0010720 positive regulation of cell development	80	ROBO2, NRP1, BMPR2, B4GALT1, EPO, DIXDC1, ILK, OTP, VLDLR, GLI3, HOXA11, GLI2, GRM5, C1QBP, DPYSL3, NEFL, EPHB2, RGS6, NDEL1, NCOA1, SEMA6A, TCF12, MAGI2, COBL, SOX11, PAX6, PROX1, ADRA2B, PAX2, UBE2V2, RARB, RAPGEF2, PDE5A, PRKD1, EPHA3, PAFAH1B1, NUMBL, NLGN1, NOTCH1, PLAG1, SEMA3A, FOXG1, SHOX2, ADRB1, ASCL1, NEUROD1, RAP1B, EPB41L5, NEUROD2, CNR1, GCNT2, ABL2, DMD, PACSIN1, BCL9L, NKX2-2, SMAD2, SPEN, LYN, SPAG9, SMAD1, NTRK2, TMEM30A, NEGR1, TGFB3, BDNF, BCL11A, NTRK3, LIMK1, RAB11A, VEGFA, NET1, PTPRD, BMP2, CLCF1, SNAI1, CNTN1, BCL2, CPEB3, FOXA2

Table S4: Most significant Gene Ontology terms (Biological Process) associated with miRNA targeted genes for BRCA.

Biological Process	Count	Genes
GO:1901699 cellular response to nitrogen compound	129	COL16A1, IRS1, IRS2, FGF1, SLC8A1, IGF1R, FGF5, FGF7, NAMPT, GRB10, SNIP1, AKT1, PRKACB, MAP3K5, PDGFRA, SMARCC1, PRKAB2, MEF2C, MAP2K1, PRKCI, IGFBP5, MTUS1, ACSL6, TSC1, FRS2, DICER1, GABRG2, SFRP1, ADCY9, COL4A1, RRAGB, COL4A6, ATP6V1A, PRKDC, SHC1, STXBP4, PXN, STC1, PIK3R3, ITPR2, FOXO4, UBR1, PIK3R1, FOXO1, SOCS2, RAP1A, SOCS1, PDGFD, PDGFC, ABL1, ATP6V1H, DRD1, PPARGC1A, ATP6V1C1, ATP6V1C2, EGR1, EGR2, EGR3, UBE2B, MYO5A, GNG12, COL1A1, COL1A2, CPS1, GNB2, COL5A2, CDK2, PDE3A, GNAS, GNB4, CPEB3, CPEB2, FGFR2, CPEB4, EIF4G1, GABRB3, GABRB2, YWHAB, PRKAG2, IDE, RYR3, GHR, PDE4B, DNMT3B, AP3S1, YWHAG, APPL1, KLF10, SREBF1, PDPK1, PDE4D, MMP2, DNMT3A, MMP3, GAB1, OPRM1, PIK3CA, RAPGEF1, RAPGEF2, CRK
GO:0097485 neuron projection guidance	116	SEMA5A, PITPNA, ACTG1, RPS6KA3, RPS6KA5, ALCAM, DPYSL2, DAG1, HOXA1, EPHB3, EPHA4, SEMA6C, MAP2K1, EPHA7, SEMA6A, EPHA8, SEMA6D, WNT5A, UNC5D, ANK3, ISL1, ANK1, CACNB1, CACNB2, MYL6, CACNB3, COL4A2, COL4A1, COL4A4, COL4A3, COL4A5, DLX5, GATA3, EFNB2, EFNB3, FLRT2, FLRT3, PLXNA2, ST8SIA4, ABL1, PLXNA1, SRGAP2, SCN3B, SPTBN1, EGR2, TGFB2, BCL11B, BDNF, ST8SIA2, SIAH1, PTPRC, DLG3, COL5A1, COL5A3, COL5A2, COL9A1, CNTN4, ITGB1, ROBO2, NRP1, NRP2, CNTNAP1, YWHAB, WASL, LAMC1, GLI3, RND1, ROBO1, BOC, ITGAV, RAC1, NEO1, SPTAN1, NCK1, ARHGEF12, DCC, TRPC4, ITGA2, NOG, PAX6, ETV1, ETV4, RHOA, ENAH, CREB1, COL2A1, DCX, NUMB, COL6A3, MYH9, MET, ITGA9, LAMA2, SRC, NRXN1, SEMA3A, CRMP1, CDC42, PAK1, RELN
GO:0007411 axon guidance	116	SEMA5A, PITPNA, ACTG1, RPS6KA3, RPS6KA5, ALCAM, DPYSL2, DAG1, HOXA1, EPHB3, EPHA4, SEMA6C, MAP2K1, EPHA7, SEMA6A, EPHA8, SEMA6D, WNT5A, UNC5D, ANK3, ISL1, ANK1, CACNB1, CACNB2, MYL6, CACNB3, COL4A2, COL4A1, COL4A4, COL4A3, COL4A5, DLX5, GATA3, EFNB2, EFNB3, FLRT2, FLRT3, ST8SIA4, PLXNA2, ABL1, PLXNA1, SRGAP2, SCN3B, SPTBN1, EGR2, TGFB2, BCL11B, BDNF, ST8SIA2, SIAH1, PTPRC, DLG3, COL5A1, COL5A3, COL5A2, COL9A1, CNTN4, ITGB1, ROBO2, NRP1, CNTNAP1, NRP2, YWHAB, LAMC1, WASL, GLI3, RND1, ROBO1, BOC, ITGAV, RAC1, NEO1, SPTAN1, NCK1, ARHGEF12, DCC, TRPC4, ITGA2, NOG, PAX6, ETV1, ETV4, RHOA, ENAH, CREB1, COL2A1, DCX, NUMB, COL6A3, MYH9, MET, ITGA9, LAMA2, SRC, NRXN1, SEMA3A, CRMP1, CDC42, PAK1, RELN
GO:0071417 cellular response to organonitrogen compound	122	COL16A1, IRS1, IRS2, FGF1, SLC8A1, IGF1R, FGF5, FGF7, NAMPT, GRB10, AKT1, PRKACB, PDGFRA, SMARCC1, MEF2C, PRKCI, PRKAB2, MAP2K1, IGFBP5, MTUS1, ACSL6, TSC1, FRS2, GABRG2, SFRP1, ADCY9, COL4A1, RRAGB, COL4A6, ATP6V1A, PRKDC, STXBP4, SHC1, PXN, ITPR2, STC1, PIK3R3, FOXO4, UBR1, PIK3R1, FOXO1, SOCS2, RAP1A, SOCS1, PDGFD, PDGFC, ABL1, ATP6V1H, DRD1, ATP6V1C1, ATP6V1C2, EGR1, EGR2, EGR3, UBE2B, MYO5A, GNG12, COL1A1, COL1A2, CPS1, GNB2, COL5A2, GNB4, PDE3A, GNAS, CPEB3, CPEB2, FGFR2, CPEB4, EIF4G1, GABRB3, GABRB2, YWHAB, PRKAG2, IDE, RYR3, GHR, PDE4B, DNMT3B, AP3S1, YWHAG, APPL1, KLF10, SREBF1, PDPK1, MMP2, PDE4D, MMP3, DNMT3A, GAB1, OPRM1, PIK3CA, RAPGEF1, RAPGEF2, CRK, VAMP2, CEBPB, CAB39, AKAP7, AKAP6, ADCY2, ADCY1, HDAC9, PAK1, GNG2, SH3BP4, MSX1, EIF4E, PTPN1, ATP2B4, KLF4, BAIAP2, KLF3, RAB10, COL3A1, RAD51, RPS6KB1, WT1, KRAS, LPIN1, PTPN2, HCN1
GO:0010720 positive regulation of cell development	107	SEMA5A, STMN2, HOXA11, PREX1, PALM, EPHA4, MEF2C, PRKCI, SEMA6A, DSCAM, WNT5A, ACSL6, RFX3, ISLR2, SOX11, DICER1, ADCYAP1, TIAM1, MAP1B, IL6ST, UTRN, EPHA3, NUMBL, FOXG1, ADRB1, HIF1A, NEUROD1, EPB41L5, RAP1A, BCL9L, NKX2-2, SMAD2, TGFB2, XRCC4, TGFB3, BDNF, BCL11A, LIF, COL1A1, NET1, PTPRD, BMP2, CAPRIN1, ID2, PDE3A, HOXD3, CPEB3, ITGB1, RET, ROBO2, NRP1, BMPR2, B4GALT1, RND2, TRIOBP, GLI3, ROBO1, DNMT3B, RAC1, NDEL1, NCOA1, FGA, EDN1, CAMK1D, DCC, TCF12, FGG, PAX6, OPRM1, CBFA2T2, RHOA, ETV5, IFNG, KIT, IL1RAPL1, RAPGEF1, NUMB, RARB, RAPGEF2, ARHGEF7, SHANK3, PAFAH1B1, NLGN1, RGS14, PLAG1, SEMA3A, SEZ6, AKAP6, ASCL1, LRRC16A, CDC42, MAPK9, RELN, CNR1, NTF3, BNIP2, HES1, DMD, SPEN, NTRK2

Table S4: Most significant Gene Ontology terms (Biological Process) associated with miRNA targeted genes for KIRC.

Biological Process	Count	Genes
GO:0048729 tissue morphogenesis	117	SEMA5A, PLXND1, CSF1, IHH, RBPJ, FGF2, IGF1R, MYLK, SOX2, FGF7, SALL1, KDR, DAG1, SOX8, BTRC, PITX2, TP63, MEF2A, ACVR1, MED1, MEF2C, CASR, OSR1, CTNNBIP1, TPM1, WNT5A, SOX11, FRS2, DICER1, ISL1, MYCN, COL4A1, ZFPM2, VCL, FREM2, NOTCH2, CSF1R, PDGFB, LIN7C, HIF1A, ARHGAP12, SOCS3, RDH10, DVL1, NPHP3, DVL3, WNT1, SMAD2, JAG2, SMAD3, JAG1, TGFB1, JUP, FZD5, FOXF1, WNT3A, PTCH1, NFATC3, IGF1, ESR1, SMAD7, NFATC4, SMARCA4, IL6, VANGL2, BCL2, GRB2, FGFR2, FGFR1, NRP1, RARG, FLT1, B4GALT1, NRARP, CA2, ZIC3, SUFU, HEY2, RSPO2, RSPO3, TEAD1, NKX3-1, IL10, EDN1, CHUK, NOG, MTSS1, PAX2, SERPINB5, TGFBR2, TGFBR3, ALDH1A3, HAND1, MET, YAP1, LUZP1, SEMA3A, SRF, DLL1, LRP6
GO:0002009 morphogenesis of an epithelium	103	SEMA5A, PLXND1, CSF1, IHH, RBPJ, FGF2, IGF1R, SOX2, FGF7, SALL1, KDR, DAG1, SOX8, PITX2, BTRC, TP63, MEF2A, ACVR1, MED1, CASR, MEF2C, OSR1, CTNNBIP1, WNT5A, FRS2, SOX11, DICER1, MYCN, COL4A1, FREM2, VCL, NOTCH2, CSF1R, PDGFB, LIN7C, HIF1A, ARHGAP12, SOCS3, RDH10, DVL1, DVL3, NPHP3, WNT1, JAG2, SMAD3, JAG1, TGFB1, JUP, FZD5, FOXF1, WNT3A, PTCH1, NFATC3, IGF1, ESR1, NFATC4, IL6, VANGL2, BCL2, GRB2, FGFR2, FGFR1, NRP1, RARG, B4GALT1, FLT1, NRARP, ZIC3, CA2, SUFU, RSPO2, RSPO3, TEAD1, NKX3-1, IL10, EDN1, CHUK, NOG, PAX2, SERPINB5, MTSS1, TGFBR2, ALDH1A3, HAND1, MET, YAP1, LUZP1, SRF, SEMA3A, DLL1, LRP6, DLL4, PAK1, NRAS, SLC12A2, EYA1, MPP5, TBX5, MIB1, VEGFA
GO:0048598 embryonic morphogenesis	124	GFI1, IHH, FGF2, AMOT, SOX2, HOXA10, SALL1, HOXA3, HOXA1, PITX2, TP63, MEF2A, ACVR1, MED1, PDGFRA, MEF2C, MBNL1, OSR1, WNT5A, SOX11, FRS2, DICER1, DKK1, HSPG2, RUNX2, BCR, MYCN, TP53, HOXB6, NOTCH2, DLX2, CUL3, CHRD, GATA2, HIF1A, NEUROD1, SOCS3, RDH10, MYO6, DVL1, NPHP3, DVL3, ALX1, OTX2, OTX1, WNT1, SMAD2, JAG2, SMAD3, JUP, FZD5, FOXF1, WNT3A, PTCH1, FZD6, PBX2, SMARCA4, VANGL2, MAFB, SP1, GNAQ, SP3, GNAS, GRB2, SP8, FGFR2, FGFR1, RARG, FLT1, FOXE1, CHD7, SOBP, HOXC11, CYP26B1, LMBR1, SIX4, ZIC3, SUFU, ZIC1, RSPO2, TMED2, RSPO3, RAC1, TEAD1, IL10, NCOA1, EDN1, NOG, TGFBR1, PAX2, TGFBR2, ALDH1A3, COL2A1, MMP16, PPAP2B, HAND1, GAS1, RARB, MDFI, YAP1
GO:1901699 cellular response to nitrogen compound	128	IRS1, IRS2, FGF1, FGF2, SLC8A1, IGF1R, IPO5, FGF7, CHEK1, NAMPT, GRB10, PDK4, SNIP1, PRKACA, MEF2A, PDGFRA, SMARCC1, PRKAB2, ATP6V1G1, CASR, MEF2C, MAP2K1, IGFBP5, ADIPOQ, MTUS1, ACSL6, TSC1, FRS2, DICER1, GABRG2, RRAGA, ADCY9, COL4A1, PRKAR1A, COL4A6, PHIP, RAF1, DNMT1, MAX, STC1, PIK3R3, PIK3R1, FOXO1, HSP90B1, SOCS1, PRKAR2A, EIF4EBP2, DRD1, WNT1, PPARGC1A, WDTC1, ATP6V1F, WNT10B, EGR3, JUP, UBE2B, INSR, DENND4C, MYO5A, INHBB, GNG12, SSH1, COL1A1, DIAPH1, CPEB1, COL1A2, COL5A2, CDK2, PDE3A, GNAS, GRB2, CPEB3, CPEB2, FGFR2, FGFR1, CPEB4, BCL2L1, GABRB3, GABRB2, CIITA, YWHAB, PDE3B, PRKAG2, SLC6A4, PPP1R9B, GHR, EEF2K, PDE4B, GNRHR, AP3S1, JAK2, PDE4D, STRADB, OPRM1, PIK3CA, ATP6V1B2, RAPGEF2, PRKAA1, CEBPB, CAB39
GO:0071417 cellular response to organonitrogen compound	122	IRS1, IRS2, FGF1, FGF2, SLC8A1, IGF1R, IPO5, FGF7, CHEK1, NAMPT, GRB10, PDK4, PRKACA, MEF2A, PDGFRA, SMARCC1, MEF2C, PRKAB2, ATP6V1G1, CASR, MAP2K1, IGFBP5, ADIPOQ, MTUS1, ACSL6, TSC1, FRS2, GABRG2, RRAGA, ADCY9, COL4A1, PRKAR1A, COL4A6, PHIP, RAF1, DNMT1, MAX, STC1, PIK3R3, PIK3R1, FOXO1, HSP90B1, SOCS1, PRKAR2A, EIF4EBP2, DRD1, WNT1, WDTC1, ATP6V1F, WNT10B, EGR3, JUP, UBE2B, INSR, DENND4C, MYO5A, INHBB, GNG12, SSH1, COL1A1, DIAPH1, CPEB1, COL1A2, COL5A2, PDE3A, GNAS, GRB2, CPEB3, CPEB2, FGFR2, CPEB4, BCL2L1, FGFR1, GABRB3, GABRB2, YWHAB, PDE3B, PRKAG2, PPP1R9B, SLC6A4, GHR, EEF2K, PDE4B, GNRHR, AP3S1, JAK2, STRADB, PDE4D, OPRM1, PIK3CA, ATP6V1B2, RAPGEF2, CEBPB, PRKAA1, CAB39, AKAP7, ADCY3, AKAP6, ADCY2, HDAC9

Table S4: Most significant Gene Ontology terms (Biological Process) associated with miRNA targeted genes for LGG.

Biological Process	Count	Genes
GO:0007411 axon guidance	105	SEMA5A, PITPNA, ACTG1, RPS6KA4, RPS6KA3, RPS6KA5, ALCAM, OPHN1, DPYSL4, KIF5C, DPYSL5, RPS6KA2, DPYSL2, KIF5A, RPS6KA1, DAG1, HOXA1, PLXNC1, EPHA5, EPHA4, MAP2K1, EPHA7, SEMA6A, UNC5A, EPHA8, WNT5A, ANK2, UNC5D, ANK3, RUNX3, ANK1, CACNB1, CACNB3, CACNB4, COL4A1, SCN8A, COL4A3, PRKCQ, AGAP2, GATA3, EFNB2, EFNB1, SDCBP, EFNB3, FLRT2, RRAS, FLRT3, ST8SIA4, PLXNA1, ABL2, PLCG1, SRGAP2, SCN3B, SPTBN1, SPTBN2, VASP, HSPA8, EGR2, BCL11B, BDNF, ST8SIA2, KIAA1598, LHX2, ITGA10, CNTN2, CNTNAP1, NRP2, USP33, PTPRO, PTPRM, LAMC1, WASL, CHL1, NCK2, ARHGEF11, ARHGEF12, TRPC1, ETV1, RGMB, RHOC, RGMA, DCX, MYH9, GAS1, MET, SRC, ROCK2, NTN4, ABLIM1, PAK1, ABLIM3, AP2S1, MAPK1, APBB2, PAK2, LGI1, LIMK1, SEMA4F, AP2B1, VEGFA
GO:0097485 neuron projection guidance	105	SEMA5A, PITPNA, ACTG1, RPS6KA4, RPS6KA3, RPS6KA5, ALCAM, OPHN1, DPYSL4, KIF5C, DPYSL5, RPS6KA2, DPYSL2, KIF5A, RPS6KA1, DAG1, HOXA1, PLXNC1, EPHA5, EPHA4, MAP2K1, EPHA7, SEMA6A, UNC5A, EPHA8, WNT5A, ANK2, UNC5D, ANK3, RUNX3, ANK1, CACNB1, CACNB3, CACNB4, COL4A1, SCN8A, COL4A3, PRKCQ, AGAP2, GATA3, EFNB2, EFNB1, SDCBP, EFNB3, FLRT2, RRAS, FLRT3, ST8SIA4, PLXNA1, ABL2, PLCG1, SRGAP2, SCN3B, SPTBN1, SPTBN2, VASP, HSPA8, EGR2, BCL11B, BDNF, ST8SIA2, KIAA1598, LHX2, ITGA10, CNTN2, NRP2, CNTNAP1, USP33, PTPRO, PTPRM, WASL, LAMC1, CHL1, NCK2, ARHGEF11, ARHGEF12, TRPC1, ETV1, RHOC, RGMB, RGMA, DCX, GAS1, MYH9, MET, SRC, ROCK2, NTN4, ABLIM1, PAK1, ABLIM3, AP2S1, MAPK1, APBB2, PAK2, LGI1, LIMK1, SEMA4F, AP2B1, VEGFA
GO:0007399 nervous system development	81	SEMA5A, CHRM2, APP, NRSN1, GMFB, MYT1L, GDA, VLDLR, RAPGEFL1, DPYSL4, OPHN1, DPYSL5, POFUT1, DPYSL2, SMPD1, ENC1, TRIM3, EP300, PCDHAC2, PCDHAC1, CSGALNACT1, MYLIP, SMARCC1, SEMA6A, DSCAM, TMOD2, DYRK1A, PCDHA11, PCDHA10, SCN8A, DCX, RAPGEF5, ARHGEF7, NBL1, NAIP, HDAC4, NOTCH2, NUMBL, NRN1, KIAA2022, NR2C2, CPLX2, NEUROD2, PURA, EFNB3, PCDHA1, ST8SIA4, PCDHA5, SPOCK1, PCDHA4, PCDHA3, PCDHA2, SCN3B, MARK4, CAMK2G, PCDHA8, MEF2D, PCDHA7, PCDHA6, LGI1, MBD5, JAG1, BDNF, SEMA4B, ST8SIA2, STAT3, LIMK1, CRIM1, CABLES1, SEMA4F, INHBA, SEMA4G, ARHGAP26, VEGFA, NELL1, CNTN3, FGF13, SCN2A, NEUROG1, FGF12, SCN2B
GO:0045664 regulation of neuron differentiation	93	RET, SEMA5A, FOXA1, SNAP25, APP, BMPR2, PTPRO, PTEN, DIXDC1, VLDLR, PREX1, STK11, SYNGAP1, SALL1, DPYSL2, CFL2, TIMP2, TRIM67, NDEL1, NCOA1, MED1, EPHA4, ZHX2, TGIF2, DSCAM, TCF12, MAGI2, WNT5A, SOX11, POU3F2, CIT, TIAM1, RAP2A, IL1RAPL1, RARB, RAPGEF2, KCTD11, ULK1, PRKD1, MAPT, NBL1, SHANK3, PAFAH1B1, NOTCH3, NUMBL, NOTCH1, SDC2, FOXG1, LRP4, RNF6, GATA3, DLL1, LRP8, ADCY6, NEUROD1, NEUROD2, CNR1, BNIP2, SPOCK1, ABL2, DMD, PDLIM5, NKX2-2, TFAP2A, SMAD1, NTRK2, JAG1, TMEM30A, EYA1, BCL11B, BDNF, SS18L1, LIMK1, SEMA4F, SSH2, MIB1, VEGFA, PTPRD, BMP2, CAPRIN1, NEDD4, FKBP1B, CAPRIN2, BCL2, ID4, CNTN2, VIM, CPEB3, FGF13, NEUROG1, NEUROG2, SPG20, CDK5R1
GO:0022604 regulation of cell morphogenesis	92	SEMA5A, FOXA1, RET, BMPR2, TGFB1I1, PTPRO, PTEN, DIXDC1, PREX1, STK11, SYNGAP1, ZMYM4, CFL2, KDR, TBC1D10C, EVI5L, SH3D19, NDEL1, EPHA4, DSCAM, WNT5A, ARHGEF18, AXIN2, POU3F2, CIT, TIAM1, TBC1D1, RAP2A, TBC1D20, PAX8, IL1RAPL1, MYH9, RAPGEF2, STRAP, ULK1, MAPT, ARHGEF7, ALDOA, SHANK3, PAFAH1B1, CSF1R, NUMBL, RABGAP1, NOTCH1, PALM2, SDC2, LEF1, LRP4, RNF6, LRP8, CAMSAP1, FGD1, PPP2CA, FGD4, MAPK9, FGD5, FGD6, GCNT2, PDLIM5, TBC1D17, WASF3, TBC1D15, ATG5, SMAD2, SMAD1, NTRK2, ZRANB1, LIMK1, LIF, SEMA4F, PARVA, SSH2, TBX5, VEGFA, SMAD7, FMNL3, PTPRD, DAB2, BMP2, FMNL2, BAMBI, NEDD4, CAPRIN1, DLC1, TAOK2, CAPRIN2, CNTN2, BRWD1, FGF13, CDC42SE2, SPG20, CDK5R1

Table S4: Most significant Gene Ontology terms (Biological Process) associated with miRNA targeted genes for LIHC.

Biological Process	Count	Genes
GO:0048729 tissue morphogenesis	130	FOXA1, NCKAP1, PLXND1, IHH, NR3C1, FGF2, IGF1R, MYLK, HOXA11, SOX2, EDNRA, FGF7, SALL1, SOX17, KDR, DAG1, SOX9, BTRC, PITX2, TP63, MED1, CASR, CTNNBIP1, WNT5A, SOX11, FRS2, DICER1, ISL1, SFRP2, MYCN, COL4A1, HOXB4, ZFPM2, VCL, FREM2, EPHA2, NOTCH2, SHOX2, PDGFB, LIN7C, ARHGAP12, EPB41L5, PPP3R1, RDH10, DVL1, NPHP3, DVL3, WNT1, SMAD2, TFAP2A, STAT5A, TFAP2C, SMAD3, JAG1, FZD5, WNT3A, PTCH1, NFATC3, IGF1, ESR1, SMAD7, IL6, BMP2, CXCL12, VANGL2, BCL2, GRB2, FGFR2, FGFR1, NRP1, RYR2, RARG, CLIC4, FLT1, B4GALT1, GLI3, HEY1, CA2, ZIC3, HEY2, RSPO2, RSPO3, TEAD1, TEAD2, PHACTR4, IL10, EDN1, CHUK, NCOA3, NRG1, PROX1, MTSS1, PAX2, TGFBR3, HAND2, HAND1, CD44, YAP1, FOXC1, LUZP1
GO:0002009 morphogenesis of an epithelium	112	FOXA1, PLXND1, IHH, NR3C1, FGF2, IGF1R, HOXA11, SOX2, EDNRA, FGF7, SALL1, SOX17, KDR, DAG1, SOX9, PITX2, BTRC, TP63, MED1, CASR, CTNNBIP1, WNT5A, FRS2, SOX11, DICER1, SFRP2, MYCN, COL4A1, HOXB4, FREM2, VCL, EPHA2, NOTCH2, PDGFB, LIN7C, ARHGAP12, EPB41L5, PPP3R1, RDH10, DVL1, DVL3, NPHP3, WNT1, TFAP2A, STAT5A, TFAP2C, SMAD3, JAG1, FZD5, WNT3A, PTCH1, NFATC3, IGF1, ESR1, IL6, BMP2, CXCL12, VANGL2, BCL2, GRB2, FGFR2, FGFR1, NRP1, RYR2, RARG, CLIC4, B4GALT1, FLT1, GLI3, ZIC3, CA2, RSPO2, RSPO3, TEAD1, TEAD2, PHACTR4, IL10, EDN1, CHUK, NCOA3, PAX2, MTSS1, HAND2, HAND1, CD44, YAP1, LUZP1, SRC, SRF, SEMA3A, DLL1, EGFR, LRP6, DLL4, GNA13, PAK1, NRAS, GPC3, SLC12A2, EYA1
GO:0007411 axon guidance	126	TRIO, PLXND1, PITPNA, RPS6KA3, KIF5C, KIF5B, DPYSL2, KIF5A, DPYSL3, DAG1, NRCAM, NRTN, HOXA1, EPHB1, SH3GL2, MNX1, EPHB3, EPHA4, SEMA6C, MAP2K1, EPHA7, SEMA6A, UNC5A, CSNK2A1, SEMA6D, WNT5A, UNC5C, ANK2, TBR1, ISL1, ANK1, CACNB1, CACNB2, COL4A2, LMX1A, COL4A1, SCN8A, COL4A5, RAF1, SPTBN4, MYCBP2, CACNA1D, CACNA1C, GATA3, EFNA4, EFNB2, CACNA1I, EFNB1, SDCBP, FLRT2, DVL1, ST8SIA4, PLXNA2, ABL1, ABL2, MYH10, SPTBN1, SPTBN2, NGFR, EGR2, FZD3, TGFB2, BCL11B, WNT3A, BDNF, ST8SIA2, SIAH2, SIAH1, EXT1, CXCL12, DLG3, COL5A1, DLG4, ITGA10, COL5A2, CNTN1, GRB2, FGFR1, NRP1, CNTNAP1, NRP2, NCAN, ITGB3, PTPRO, PTPRM, AP2A1, LAMC1, WASL, GLI3, ITGAV, CAP1, ITGA2, ETV1, RGMB, SPTB, VAV2, ENAH, CREB1, DCX, NUMB
GO:0097485 neuron projection guidance	126	TRIO, PLXND1, PITPNA, RPS6KA3, KIF5C, KIF5B, DPYSL2, KIF5A, DPYSL3, DAG1, NRCAM, NRTN, HOXA1, EPHB1, SH3GL2, MNX1, EPHB3, EPHA4, SEMA6C, MAP2K1, EPHA7, SEMA6A, UNC5A, CSNK2A1, SEMA6D, WNT5A, UNC5C, ANK2, TBR1, ISL1, ANK1, CACNB1, CACNB2, COL4A2, LMX1A, COL4A1, SCN8A, COL4A5, RAF1, SPTBN4, MYCBP2, CACNA1D, CACNA1C, GATA3, EFNA4, EFNB2, CACNA1I, EFNB1, SDCBP, FLRT2, DVL1, PLXNA2, ST8SIA4, ABL1, ABL2, MYH10, SPTBN1, SPTBN2, NGFR, EGR2, FZD3, TGFB2, BCL11B, BDNF, WNT3A, ST8SIA2, SIAH2, SIAH1, EXT1, CXCL12, DLG3, COL5A1, DLG4, ITGA10, COL5A2, CNTN1, GRB2, FGFR1, NRP1, NRP2, CNTNAP1, NCAN, ITGB3, PTPRO, PTPRM, AP2A1, WASL, LAMC1, GLI3, ITGAV, CAP1, ITGA2, ETV1, RGMB, SPTB, VAV2, ENAH, CREB1, DCX, NUMB
GO:0032989 cellular component morphogenesis	140	RB1, APP, PID1, ATP8A2, IHH, LOXL3, IGF1R, SALL1, SOX17, TNN, NRCAM, SOX9, EPHB1, EPHB3, EPHA4, MED1, HGF, SLC11A2, WNT5A, FNDC3B, DICER1, ISL1, BIN3, SLC9A6, TBC1D20, ADAM15, TYRO3, UTRN, EPHA1, SIDT2, EPHA2, GPM6A, SPTBN4, NUMBL, CNP, CUL3, SDC2, ARL3, MYCBP2, PLA2G3, STC1, FBXO45, GATA3, EPB41L5, ATXN2, PPP3R1, KIF3A, BTBD3, DVL1, MAP7, NPHP3, NPTX1, DRD2, NTNG1, TGFB2, FZD5, CADM1, BCL11B, FZD4, FZD8, HMGA2, DCLK1, SSH1, BMP2, DLG4, BCL2, FAT3, FGFR2, FERMT2, BCL2L1, NRP1, CLIC4, CNTNAP1, ITGB3, PTPRO, PTEN, LAMC1, TFCP2L1, HEY1, HEY2, BAI1, ITGAV, CAP1, DST, STRADB, HEG1, MINK1, SHROOM2, SHROOM3, NRG1, PROX1, NGF, WNT16, PAX2, SHROOM4, TGFBR1, TGFBR3, CREB1, ALS2, CDH11

Table S4: Most significant Gene Ontology terms (Biological Process) associated with miRNA targeted genes for LUAD.

Biological Process	Count	Genes
GO:0048598 embryonic morphogenesis	105	NCKAP1, IHH, FGF2, AMOT, HOXA10, GJA1, SALL1, HOXA3, SOX9, HOXA7, HOXA5, TP63, CDON, HOXA4, ACVR1, MED1, PDGFRA, OSR2, MBNL1, WNT5A, SOX11, FRS2, DICER1, HSPG2, RUNX2, BCR, HOXB3, NOTCH2, NOTCH1, INSIG2, DLX5, INSIG1, CUL3, TSHZ1, FOXG1, SHOX2, TWIST1, PSEN1, HIF1A, NEUROD1, RDH10, DVL1, OTX1, WNT1, FZD3, SMAD3, FOXF2, WNT3A, PTCH1, FZD6, SMARCA4, VANGL2, MAFB, SP1, GNAQ, GRB2, FGFR2, FGFR1, RYR2, RARG, CHD7, C2ORF49, SOBP, AFF3, HOXC11, CYP26B1, HHEX, SIX4, ZIC1, RSPO2, TMED2, RSPO3, TEAD1, NKX3-2, NCOA1, EDN1, TGFBR1, PAX2, TGFBR2, ALDH1A3, PPAP2B, RARB, YAP1, LUZP1, SATB2, SRF, LRP4, HOXD11, DLL1, HOXD10, RNF2, LRP6, DLL4, FRZB, GNA12, SLITRK6, LRIG1, PRRX1, EYA1, WNT7A
GO:0048729 tissue morphogenesis	98	NCKAP1, CSF1, IHH, NR3C1, FGF2, IGF1R, MYLK, GJA1, FGF7, SALL1, KDR, DAG1, SOX9, BTRC, HOXA5, TP63, ACVR1, MED1, CASR, CTNNBIP1, WNT5A, SOX11, FRS2, DICER1, NPNT, ZFPM2, VCL, NOTCH2, NOTCH1, SHOX2, TWIST1, PSEN1, HIF1A, ARHGAP12, PPP3R1, RDH10, DVL1, WNT1, WNT10A, SMAD3, JAG1, WNT3A, PTCH1, NFATC3, IGF1, ESR1, SMAD7, NFATC4, SMARCA4, IL6, VANGL2, BCL2, GRB2, FGFR2, FGFR1, NRP1, RYR2, RARG, BMP10, B4GALT1, NRARP, HHEX, CA2, HEY2, RSPO2, RSPO3, TEAD1, EDN1, CHUK, CYP7B1, MTSS1, PAX2, TGFBR2, TGFBR3, ALDH1A3, MET, YAP1, FOXC1, LUZP1, SEMA3A, SRF, HOXD11, DLL1, TTN, LRP6, DLL4, PAK1, RXRA, FRZB, SLC12A2, EYA1, KLHL3, POU4F1, MIB1, VEGFA, FKBP1A, KRAS, LGR4
GO:0045664 regulation of neuron differentiation	103	APP, DIXDC1, SALL1, DPYSL2, ARHGDIA, CFL2, SOX9, CDON, SOX5, EPHB3, MED1, EPHA4, WNT5A, OMG, SOX11, DICER1, POU3F2, TIAM1, LMX1A, RUFY3, ULK2, NOTCH1, FOXG1, SHOX2, PSEN1, RTN4, NEUROD1, DVL1, ABL2, SMAD1, ZFHX3, JAG1, BCL11B, WNT3A, BDNF, BCL11A, PTK2, BMP6, GFAP, PTPRD, CCDC88A, IL6, CAPRIN1, CAPRIN2, BCL2, FAT3, CNTN4, TCF3, CPEB3, FGFR1, RET, ROBO2, SNAP25, NRP1, BMPR2, LRRK2, PTEN, AATK, SLC6A4, ROBO1, PTPRG, HEY2, NPTN, TIMP2, NEFL, YWHAH, NDEL1, NCOA1, TGIF2, CAMK1D, ETV5, CIT, IL1RAPL1, RARB, SHANK3, PAFAH1B1, NLGN1, SEMA3A, LRP4, TTL, DLL1, LRP8, RELN, CNR1, BNIP2, SPOCK1, DMD, PDLIM5, SPAG9, NTRK2, EYA1, NEGR1, IRX3, DAB2IP, WNT7A, MIB1, VEGFA, GRIN1, MEIS1, FKBP1B
GO:0009887 organ morphogenesis	103	NCKAP1, CSF1, ZFAND5, IHH, FGF2, TNF, CRKL, TGM1, HOXA3, EP300, SOX9, HOXA7, HOXA5, SOX4, EPHB4, TP63, HOXA4, PDGFRA, OSR2, ARID5B, SOX11, HSPG2, RUNX2, EREG, EDAR, COL8A2, HOXB3, NOTCH2, TSHZ3, SHOX2, TWIST1, PSEN1, EFNB2, NEUROD1, SGPL1, BCL2L11, HOXC8, WNT10A, SMAD3, FOXF2, BCL11B, PTCH1, INSR, MYO5A, PTK2, SMARCA4, VANGL2, COL5A1, SP1, NF1, PAM, FGFR2, FGFR1, NRP1, RARG, EDA, THRB, ONECUT2, FHL1, CHD7, PTEN, HOXC11, SLC6A4, CYP26B1, SIX4, CA2, NKX3-2, CHUK, ITGA2, PAX3, AXIN2, TGFBR1, PAX2, TGFBR2, TGFBR3, NCOR2, ALDH1A3, RARB, SHANK3, PAFAH1B1, FOXC1, TUFT1, SATB2, SRF, GBA, LRP4, HOXD11, HOXD10, TTN, LRP6, MFN2, MAPK1, PLEKHA1, PRRX1, EYA1, CDX2, WNT7A, ACVR2B, VEGFA, FKBP1A
GO:0033674 positive regulation of kinase activity	112	CSF1, ZAK, IRS1, NRK, FGF2, TNF, CRKL, CCND3, CCND2, CCND1, PIM1, MAP3K9, PRKACA, MAP3K7, PRKACB, MAP3K4, MAP3K5, TSPYL2, PDGFRA, CARD10, MAP2K4, EPHA4, MAP2K1, FBXW7, ACSL1, DDX11, WNT5A, FRS2, PRLR, PDIA6, EREG, CDC25B, TOM1L1, ADAM9, RAF1, TGFA, PSEN1, IQGAP1, ADRB2, PRKCZ, C5, IRAK2, PRKAR2A, UBC, SSR1, PLCG1, GADD45B, GADD45A, FZD4, WNT3A, INSR, EDEM1, PTK2, GADD45G, CCDC88A, VANGL2, TPP1, MAP3K14, MAP3K12, FGFR1, CDKN1A, CD40, FLT3, LRRK2, SRPR, HTR2A, GRM1, GHR, GRM5, EDN1, STRADB, ZFP91, ADRA2A, TGFBR1, TGFBR2, VAV2, ZEB2, MDFIC, KIT, PDE5A, MAPRE3, CRK, MET, PRKAA1, CAB39, ADCY2, ADCY1, LRP8, PAK1, RELN, ERBB3, ERBB4, MAPK1, CSK, ZNF622, PAK2, SASH1, SNCA, SPAG9, MAP3K3

Table S4: Most significant Gene Ontology terms (Biological Process) associated with miRNA targeted genes for PAAD.

Biological Process	Count	Genes
GO:0007411 axon guidance	84	NRP1, NRP2, PLXND1, YWHAB, USP33, PTPRO, ROBO1, RPS6KA5, ALCAM, OPHN1, RPS6KA1, HOXA1, EPHB2, EPHB1, NEO1, SH3GL2, AP2M1, EPHB3, EPHA4, SEMA6A, ITGA2, SEMA6D, ANK2, TBR1, UNC5D, ANK3, ISL1, MAPK8IP3, ANK1, VAV2, ENAH, CACNB2, CREB1, COL2A1, LMX1A, COL4A3, DCX, NUMB, ITGA5, MET, SEMA7A, NRXN1, SEMA3B, SEMA3E, CACNA1C, GATA3, EGFR, CDC42, EFNB2, SDCBP, NRAS, RELN, FLRT2, FLRT3, ST8SIA4, ABL2, OTX2, NCAM1, FYN, SRGAP2, SCN3B, MYH10, PAK2, CLASP2, NGFR, SMAD4, WNT3A, BDNF, SIAH2, LIMK1, SIAH1, SEMA4F, VEGFA, KLF7, NFASC, EFNA3, DLG3, NFIB, PTPRA, KRAS, GRB2, LHX4, AGRN, CDK5R1
GO:0097485 neuron projection guidance	84	NRP1, NRP2, PLXND1, YWHAB, USP33, PTPRO, ROBO1, RPS6KA5, ALCAM, OPHN1, RPS6KA1, HOXA1, EPHB2, EPHB1, NEO1, SH3GL2, AP2M1, EPHB3, EPHA4, SEMA6A, ITGA2, SEMA6D, ANK2, TBR1, UNC5D, ANK3, ISL1, MAPK8IP3, ANK1, VAV2, ENAH, CACNB2, CREB1, COL2A1, LMX1A, COL4A3, DCX, NUMB, ITGA5, MET, SEMA7A, NRXN1, SEMA3B, SEMA3E, CACNA1C, GATA3, EGFR, CDC42, EFNB2, SDCBP, NRAS, RELN, FLRT2, FLRT3, ST8SIA4, ABL2, OTX2, NCAM1, FYN, SRGAP2, SCN3B, MYH10, PAK2, CLASP2, NGFR, SMAD4, BDNF, WNT3A, SIAH2, LIMK1, SIAH1, SEMA4F, VEGFA, KLF7, NFASC, EFNA3, DLG3, NFIB, PTPRA, KRAS, GRB2, LHX4, AGRN, CDK5R1
GO:0045664 regulation of neuron differentiation	89	RET, FOXA1, SNAP25, NRP1, BMPR2, PTPRO, DIXDC1, ROBO1, SOX2, EDNRB, CFL2, HEY2, EPHB2, RGS6, CDON, SOX5, EPHB3, NDEL1, NCOA1, MEF2A, EPHA4, MEF2C, PRKCI, DSCAM, MAGI2, SOX11, TBR1, POU3F2, ISL1, MAPK8IP3, NME1, CIT, TIAM1, RAP2A, ZEB1, IFNG, LMX1A, GORASP1, RARA, RARB, RAPGEF2, MAPT, SHANK3, ARF6, SEMA7A, NOTCH1, SDC2, GATA3, GATA2, TTL, LRP8, ADCY6, RAP1B, NEUROD2, RELN, CNR1, INPP5J, ABL2, FYN, PDLIM5, NKX2-2, SPAG9, SMAD1, NGFR, NTRK2, ZFHX3, JAG1, EYA1, NEGR1, WNT3A, BDNF, BCL11A, SS18L1, NTRK3, LIMK1, FN1, SEMA4F, GDF6, PHOX2B, VEGFA, GRIN1, MEIS1, TLX3, NEDD4, ID2, ID4, CPEB3, FGF13, CDK5R1
GO:0007389 pattern specification process	80	FOXA1, NRP1, BTG2, PGAP1, BMPR2, PLXND1, BMI1, HOXC11, HOXA10, LFNG, EDNRA, CYP26B1, ZIC1, HEY2, HOXA3, EP300, HOXA1, RNF111, HOXA5, TP63, CDON, MEF2A, ACVR1, MEF2C, TCF15, FST, TBR1, FRS2, RPGRIP1L, ISL1, DUSP6, TGFBR1, GREM1, ZEB2, SFRP1, ZEB1, HOXB3, AIDA, HOXB8, TP53, MDFI, HOXB5, NOTCH2, NOTCH1, SATB2, SRF, LRP5, STC1, HTT, SEMA3E, DLL4, PPP3R1, RELN, ARL13B, ERBB4, DVL2, OTX2, HOXC6, SMAD2, SMAD1, SMAD4, FZD5, EYA1, WNT3A, PTCH1, PBX2, NR2F2, HIPK1, TBX5, SMAD5, ACVR2B, ACVR2A, VEGFA, VANGL2, APC, SNAI1, SP8, BCOR, SSBP3, BMPR1A
GO:0009887 organ morphogenesis	82	NRP1, SETD2, EDA, THRB, ONECUT2, CSF1, CHD7, TCF21, HOXC11, SLC8A1, LFNG, CYP26B1, SERP1, SIX4, ZMIZ1, BSG, HOXA3, TNFSF11, EP300, EPHB2, EPHB1, HOXA5, TP63, DGCR2, EDARADD, PDGFRA, SEMA6A, TCF15, ITGA2, FST, ARID5B, SOX11, MAPK8IP3, TGFBR1, EDAR, ZEB1, COL2A1, MMP16, TRAF6, PAX9, HOXB3, RARB, HOXB8, SHANK3, STK40, MDFI, HOXB5, NOTCH2, SATB2, SRF, COL11A2, LRP5, PRKX, EFNB2, BCL2L11, HLX, DVL2, BAK1, SMAD2, IRX5, EYA1, PTCH1, INSR, LIF, VEGFC, HIPK1, ACVR2B, VEGFA, COL1A1, FRAS1, VANGL2, COL1A2, SP1, DLC1, ID2, SP3, NF1, ID3, LHX4, NF2, BCOR, BMPR1A

Table S4: Most significant Gene Ontology terms (Biological Process) associated with miRNA targeted genes for PRAD.

Biological Process	Count	Genes
GO:0097485 neuron projection guidance	126	SEMA5A, PLXND1, RPS6KA3, RPS6KA5, ALCAM, OPHN1, RPS6KA2, DPYSL2, DAG1, NRTN, HOXA1, EPHB2, EPHB1, SH3GL2, EPHA4, SEMA6A, UNC5A, SEMA6D, WNT5A, UNC5C, ANK2, TBR1, UNC5D, ANK3, ISL1, MAPK8IP3, ANK1, CACNB1, CACNB2, COL4A2, LMX1A, COL4A1, COL4A4, SCN8A, COL4A5, SPTBN4, SEMA7A, CACNA1D, CACNA1C, GATA3, EFNB2, SDCBP, FLRT2, FLRT3, PLXNA2, ST8SIA4, ABL1, PLXNA1, ABL2, MYH11, OTX2, PLCG1, SRGAP2, SCN3B, MYH10, NGFR, FZD3, BCL11B, BDNF, WNT3A, ST8SIA2, SIAH1, EFNA3, COL5A2, GRB2, COL9A3, LHX4, CNTN4, NRP1, NRP2, YWHAB, ITGB3, PTPRO, AP2A1, GLI2, ZIC2, CHL1, BOC, NCK2, ITGAV, RAC1, NEO1, NCK1, CAP1, FOXD1, ITGA2, ETV1, RHOA, RHOB, VAV2, ENAH, CREB1, DCX, MYH9, B3GNT1, ITGA5, MET, NRXN1, SEMA3A, SEMA3B
GO:0007411 axon guidance	126	SEMA5A, PLXND1, RPS6KA3, RPS6KA5, ALCAM, OPHN1, RPS6KA2, DPYSL2, DAG1, NRTN, HOXA1, EPHB2, EPHB1, SH3GL2, EPHA4, SEMA6A, UNC5A, SEMA6D, WNT5A, UNC5C, ANK2, TBR1, UNC5D, ANK3, ISL1, MAPK8IP3, ANK1, CACNB1, CACNB2, COL4A2, LMX1A, COL4A1, COL4A4, SCN8A, COL4A5, SPTBN4, SEMA7A, CACNA1D, CACNA1C, GATA3, EFNB2, SDCBP, FLRT2, FLRT3, ST8SIA4, PLXNA2, ABL1, PLXNA1, ABL2, MYH11, OTX2, PLCG1, SRGAP2, SCN3B, MYH10, NGFR, FZD3, BCL11B, WNT3A, BDNF, ST8SIA2, SIAH1, EFNA3, COL5A2, GRB2, COL9A3, LHX4, CNTN4, NRP1, NRP2, YWHAB, ITGB3, PTPRO, AP2A1, GLI2, ZIC2, CHL1, BOC, NCK2, ITGAV, RAC1, NEO1, NCK1, CAP1, FOXD1, ITGA2, ETV1, RHOA, RHOB, VAV2, ENAH, CREB1, DCX, MYH9, B3GNT1, ITGA5, MET, NRXN1, SEMA3A, SEMA3B
GO:0045664 regulation of neuron differentiation	128	SEMA5A, GFI1, DIXDC1, EDNRB, SALL1, DPYSL2, CFL2, SOX9, EPHB2, RGS6, MEF2A, EPHA4, MEF2C, DSCAM, MAGI2, WNT5A, OMG, ACSL6, SOX11, TBR1, DICER1, POU3F2, ISL1, MAPK8IP3, LMX1A, RUFY3, MAP1B, UBE2V2, ULK2, UTRN, EPHA3, RTN4R, NUMBL, SEMA7A, NOTCH1, SDC2, FOXG1, GATA3, GATA2, NEUROD1, RAP1B, INPP5J, ABL2, NKX2-2, TFAP2A, LYN, SMAD1, NGFR, ZFHX3, SNAPIN, BCL11B, WNT3A, BDNF, BCL11A, SS18L1, FN1, SSH2, GDF6, SSH1, PTPRD, IL6, ID2, CAPRIN2, BCL2, ZNF536, FAT3, CNTN4, CPEB3, FGF13, RET, SNAP25, NRP1, BMPR2, PTPRO, RND2, GLI2, NEFL, NEFM, TRIM67, NDEL1, NCOA1, ACTR3, TCF12, NGF, ADRA2B, RHOA, CIT, RAP2A, IFNG, GORASP1, IL1RAPL1, RARA, RARB, RAPGEF2, MAPT, SHANK3, PAFAH1B1, NLGN1, SEMA3A, TTL
GO:0032989 cellular component morphogenesis	134	RB1, PID1, LOXL3, HOXA13, ANTXR1, PTPDC1, IGF1R, GJA1, SALL1, STMN1, SOX9, EPHB2, SOX6, EPHB1, MEF2A, EPHA4, MEF2C, DSCAM, SLC11A2, WNT5A, FNDC3B, ANK3, DICER1, ISL1, BIN3, TBC1D20, ADAM15, LPPR4, UTRN, EPHA2, RTN4R, SPTBN4, BRSK1, NUMBL, NOTCH1, CUL3, SDC2, ARL3, PLA2G3, STC1, SLC1A3, FBXO45, GATA3, HIF1A, EPB41L5, PPP3R1, SPAST, MAP2, KIF3A, BTBD3, MAP7, NPHP3, SRGAP2, NTNG1, FZD5, CADM1, BCL11B, FZD4, FZD7, FN1, FZD8, HMGA2, SMARCA4, SSH1, NR4A2, AHI1, BCL6, BCL2, SNAI1, SNAI2, FAT3, CNTN4, FGFR3, PICALM, BCL2L1, NRP1, ITGB3, DOCK7, PTPRO, GLI2, TCTN3, SIX2, NEFL, ITGAV, RAC1, ATP7A, RAB8A, DACT1, NDEL1, CAP1, ACTR3, SS18, DST, HEG1, NGF, RHOA, TGFBR1, GREM1, CREB1, RP2
GO:0048598 embryonic morphogenesis	125	NCKAP1, GFI1, HOXA13, HOXA11, HOXA10, GJA1, SALL1, FGF9, HOXA1, SOX9, EPHB2, PITX1, HOXA5, MEF2A, ACVR1, PDGFRA, MEF2C, MBNL1, WNT5A, SOX11, FRS2, RPGRIP1L, DICER1, FOXP4, RUNX2, SFRP1, MYCN, HOXB4, APLNR, HOXB3, HOXB8, TP53, HOXB5, NOTCH1, INSIG2, CUL3, DLX6, FOXG1, CHRD, GATA3, GATA2, HIF1A, NEUROD1, EPB41L5, SOCS3, RDH10, DVL2, NPHP3, DVL3, OTX2, WNT1, SMAD2, JAG2, TFAP2A, FZD3, FZD5, WNT3A, PTCH1, PBX2, SMARCA4, AHI1, VANGL2, MAFB, SP1, ID2, SP3, GNAS, GRB2, SP8, BMPR1A, RYR2, RARG, FOXE1, CHD7, SOBP, AFF3, HOXC11, GLI2, CYP26B1, LMBR1, SIX4, ZIC3, SUFU, SIX2, TMEM107, RSPO2, TMED2, RSPO3, RAC1, TEAD1, DACT1, IL10, NCOA1, EDN1, TGFBR1, TGFBR2, MMP16, PPAP2B, HAND1, RARB

Table S4: Most significant Gene Ontology terms (Biological Process) associated with miRNA targeted genes for SKCM.

Biological Process	Count	Genes
GO:0097485 neuron projection guidance	66	ITGB1, GSK3B, NRP2, TRIO, LAMC1, RND1, ACTG1, RPS6KA3, CHL1, KIF5A, BOC, DAG1, ITGAV, NRCAM, RAC1, EPHA7, ARHGEF12, SEMA6A, UNC5A, CSNK2A1, SEMA6D, WNT5A, ANK2, GFRA1, ANK3, RGMB, RGMA, COL2A1, COL4A1, SCN8A, DCX, NUMB, MYH9, B3GNT1, MET, SEMA3C, ROCK2, NRXN1, SEMA3A, NTN4, CACNA1C, SEMA3F, EFNA5, EFNB2, SDCBP, FLRT3, GPC1, PLXNA2, PLXNA1, NCAM1, SRGAP2, MYH10, MYO10, BCL11B, BDNF, ST8SIA2, SIAH1, PTK2, POU4F2, VEGFA, COL3A1, NFASC, NFIB, KRAS, PVRL1, NEUROG2
GO:0007411 axon guidance	66	ITGB1, GSK3B, NRP2, TRIO, LAMC1, RND1, ACTG1, RPS6KA3, CHL1, KIF5A, BOC, DAG1, ITGAV, NRCAM, RAC1, EPHA7, ARHGEF12, SEMA6A, UNC5A, CSNK2A1, SEMA6D, WNT5A, ANK2, GFRA1, ANK3, RGMB, RGMA, COL2A1, COL4A1, SCN8A, DCX, NUMB, MYH9, B3GNT1, MET, SEMA3C, ROCK2, NRXN1, SEMA3A, NTN4, CACNA1C, SEMA3F, EFNA5, EFNB2, SDCBP, FLRT3, GPC1, PLXNA2, PLXNA1, NCAM1, SRGAP2, MYH10, BCL11B, MYO10, BDNF, ST8SIA2, SIAH1, PTK2, POU4F2, VEGFA, COL3A1, NFASC, NFIB, KRAS, PVRL1, NEUROG2
GO:0032989 cellular component morphogenesis	70	ITGB1, RB1, APP, GSK3B, ILK, LAMC1, RBPJ, IGF1R, SALL1, ITGAV, NRCAM, SOX9, RAC1, NDEL1, SS18, NKX2-8, MEF2C, DSCAM, MINK1, WNT5A, FNDC3B, ANK3, PAX2, TGFBR1, PAX8, ALS2, DCX, NUMB, MYH9, MET, SHANK3, PAFAH1B1, CUL3, SEMA3A, LEF1, PXN, FBXO45, STK4, HIF1A, LRP6, MTM1, EPB41L5, PARD6B, BTBD3, PDPN, CTNNA2, WASF1, SRGAP2, NTNG1, FOXF2, CADM1, BCL11B, FZD4, FZD7, FZD6, PARVA, MAPK14, BAIAP2, POU4F1, PTK2, RAB10, WEE1, BCL6, NFIB, ID1, ABI1, SNAI1, TEK, FGFR2, PICALM
GO:0051272 positive regulation of cellular component movement	51	RET, ITGB1, NRP2, BMPR2, ONECUT2, SERPINE1, ILK, FGF1, ETS1, IGF1R, AMOT, FGF7, ITGAV, SOX9, RAC1, JAK2, BCAS3, EDN1, SEMA6A, EDN3, DSCAM, SEMA6D, PRKCE, WNT5A, TGFBR1, PTP4A1, NUMB, MET, HBEGF, LRRC15, SEMA3A, LEF1, ACVR1B, HIF1A, HDAC9, EPB41L5, PODXL, PDPN, GCNT2, STAT5B, HSPA5, NTRK3, INSR, MAPK14, PTK2, VEGFA, MYO1C, BCL6, RPS6KB1, SNAI1, TEK
GO:0048598 embryonic morphogenesis	61	CDKN1C, NCKAP1, TCF21, CELSR1, AFF3, AMOT, SOX2, NIPBL, SALL1, SIX4, RSPO2, SOX9, RAC1, TEAD1, ACVR1, MEF2C, MBNL1, EDN1, WNT5A, EN1, SOX11, FRS2, TGFBR1, PAX2, COL2A1, MYCN, SFRP2, MMP16, PAX8, PPAP2B, HOXB7, INSIG2, INSIG1, COL11A1, CUL3, LEF1, TCF7, SHOX2, HIF1A, RNF2, LRP6, NEUROD1, EPB41L5, DVL2, OTX1, JAG2, FOXF2, PRRX1, PTCH1, FZD6, PCDH8, HIPK1, ACVR2B, GRHL2, ACVR2A, TBX2, SP1, ID2, ASXL2, KIAA1715, FGFR2

Table S4: Most significant Gene Ontology terms (Biological Process) associated with miRNA targeted genes for STAD.

Biological Process	Count	Genes
GO:0007399 nervous system development	97	SEMA5A, CHRM2, APP, GMFB, MYT1L, GDA, FGF2, RAPGEFL1, FGF5, EDNRB, OPHN1, DPYSL5, DPYSL2, EP300, PCDHAC2, PCDHAC1, CSGALNACT1, MYLIP, MBNL1, MEF2C, SEMA6A, DSCAM, TMOD2, DYRK1A, PCDHA11, PCDHA10, OLFM1, DOK4, SCN8A, MAP1B, NBL1, NOTCH2, NRN1, DLX6, KALRN, NEUROD2, PCDHA1, ST8SIA4, PCDHA5, PCDHA4, PCDHA3, NDP, PCDHA2, ZNF423, MARK4, PCDHA8, PCDHA7, PCDHA6, BDNF, ST8SIA2, SIAH1, CRIM1, INHBA, ARHGAP26, DCLK1, IL3, DLG4, CSPG5, MAFK, CNTN3, CNTN4, SCN2A, RAB5A, SCN2B, PCSK2, NRSN1, VLDLR, ROBO1, SMPD1, ENC1, TRIM3, VPS13A, FOS, ZEB2, DCX, RAPGEF5, ARHGEF7, HDAC4, NLGN1, SEMA3D, KIAA2022, NR2C2, PURA, ERBB4, PDPN, CAMK2G, MEF2D, LGI1, MBD5, SEMA4B, STAT3, LIMK1, CABLES1, SEMA4G, VEGFA, TTLL7, NEUROG1
GO:0032989 cellular component morphogenesis	123	PRDM8, RB1, APP, PID1, IHH, HOXA13, ANTXR1, CELSR2, PTPDC1, IGF1R, STK11, GJA1, SALL1, NRCAM, SOX6, BVES, EPHA4, MED1, MEF2C, DSCAM, SLC11A2, WNT5A, FNDC3B, ANK3, DICER1, SLC9A6, TBC1D20, TYRO3, LPPR4, UTRN, EPHA1, NBL1, SIDT2, CUL3, SDC2, ARL3, STC1, HIF1A, EPB41L5, ATXN2, PPP3R1, SPAST, MAP2, KIF3A, DVL1, MAP7, NPTX1, SRGAP2, PACSIN1, NTNG1, CADM1, BCL11B, FZD4, FZD7, FZD6, HMGA2, DCLK1, NR4A2, AHI1, DAB2, BMP2, BCL6, DLG4, BCL2, FAT3, CNTN4, FGFR2, FERMT2, PICALM, CNTNAP1, LRRK2, PTPRO, PTEN, LAMC1, TFCP2L1, SIX2, HEY2, BAI1, ITGAV, DNM1L, NEFH, NDEL1, DST, ITGA4, STRADB, HEG1, MINK1, SHROOM3, PAX2, SHROOM4, TGFBR1, TGFBR3, CREB1, RP2, CDH11, DCX, SHANK3, PAFAH1B1, RAB1A, POLDIP2
GO:0045664 regulation of neuron differentiation	115	SEMA5A, FOXA1, APP, DIXDC1, PREX1, STK11, EDNRB, SYNGAP1, SALL1, DPYSL2, ARHGDIA, CFL2, NRCAM, SOX5, MED1, EPHA4, MEF2C, DSCAM, MAGI2, WNT5A, OMG, SOX11, DICER1, POU3F2, ADCYAP1, TIAM1, MAP1B, UBE2V2, ULK1, PRKD1, UTRN, NBL1, SDC2, NEUROD1, NEUROD2, DVL1, INPP5J, ABL2, PACSIN1, NKX2-2, TFAP2A, SMAD1, ZFHX3, BCL11B, WNT3A, BDNF, BCL11A, SS18L1, SSH2, GFAP, PTPRD, BMP2, CAPRIN1, CAPRIN2, BCL2, ZNF536, FAT3, CNTN4, TCF3, CPEB3, FGFR1, RET, ROBO2, SNAP25, BMPR2, LRRK2, PTPRO, PTEN, AATK, VLDLR, RND2, SLC6A4, ROBO1, HEY2, TIMP2, SCARF1, YWHAH, NDEL1, ZHX2, TGIF2, TCF12, CIT, RAP2A, IL1RAPL1, RAPGEF1, RARB, RAPGEF2, SHANK3, PAFAH1B1, KANK1, NLGN1, SEMA3A, RNF6, TTL, DLL1, RELN, CNR1, BNIP2, PDLIM5, NTRK2
GO:0007411 axon guidance	106	SEMA5A, TRIO, PITPNA, RPS6KA4, RPS6KA3, RPS6KA5, OPHN1, KIF5C, DPYSL5, KIF5B, RPS6KA2, DPYSL2, KIF5A, RPS6KA1, NRCAM, SH3GL2, EPHA5, EPHA4, MAP2K1, EPHA7, SEMA6A, EPHA8, SEMA6D, WNT5A, ANK2, ANK3, RUNX3, CACNB1, COL4A1, COL4A4, SCN8A, COL4A3, RAF1, CACNA1C, EFNB2, EFNB1, SDCBP, DVL1, ST8SIA4, PLXNA2, PLXNA1, ABL2, SRGAP2, SPTBN1, SPTBN2, VASP, HSPA8, EGR2, BCL11B, WNT3A, BDNF, ST8SIA2, SIAH1, PTPRC, KIAA1598, DLG4, ITGA10, GRB2, CNTN4, FGFR1, ROBO2, CNTNAP1, NRP2, USP33, PTPRO, PTPRM, AP2A1, LAMC1, WASL, ROBO1, ITGAV, NEO1, NCK1, ARHGEF11, ARHGEF12, ITGA2, ITGA1, ETV1, RGMB, RHOC, RGMA, VAV2, ENAH, CREB1, DCX, GAS1, ROCK2, NRXN1, SEMA3A, NTN4, CDC42, ABLIM1, RELN, MAPK1, APBB2, PAK2, LGI1, LIMK1, AP2B1, POU4F2
GO:0097485 neuron projection guidance	106	SEMA5A, TRIO, PITPNA, RPS6KA4, RPS6KA3, RPS6KA5, OPHN1, KIF5C, DPYSL5, KIF5B, RPS6KA2, DPYSL2, KIF5A, RPS6KA1, NRCAM, SH3GL2, EPHA5, EPHA4, MAP2K1, EPHA7, SEMA6A, EPHA8, SEMA6D, WNT5A, ANK2, ANK3, RUNX3, CACNB1, COL4A1, COL4A4, SCN8A, COL4A3, RAF1, CACNA1C, EFNB2, EFNB1, SDCBP, DVL1, PLXNA2, ST8SIA4, PLXNA1, ABL2, SRGAP2, SPTBN1, SPTBN2, VASP, HSPA8, EGR2, BCL11B, BDNF, WNT3A, ST8SIA2, SIAH1, PTPRC, KIAA1598, DLG4, ITGA10, GRB2, CNTN4, FGFR1, ROBO2, NRP2, CNTNAP1, USP33, PTPRO, PTPRM, AP2A1, WASL, LAMC1, ROBO1, ITGAV, NEO1, NCK1, ARHGEF11, ARHGEF12, ITGA2, ITGA1, ETV1, RHOC, RGMB, RGMA, VAV2, ENAH, CREB1, DCX, GAS1, ROCK2, NRXN1, SEMA3A, NTN4, CDC42, ABLIM1, RELN, MAPK1, APBB2, PAK2, LGI1, LIMK1, AP2B1, POU4F2

Table S4: Most significant Gene Ontology terms (Biological Process) associated with miRNA targeted genes for THCA.
