# Supplementary material for: Genome-wide analysis of NGS data to compile cancer-specific panels of miRNA biomarkers
Source: PLoS One. 2018 Jul 26;13(7):e0200353. doi: 10.1371/journal.pone.0200353 (PMC6061989; doi:10.1371/journal.pone.0200353)
Supplement: S5 Table — Most significant Gene Ontology terms (Molecular Function) associated with miRNA targeted genes. (PDF) [file pone.0200353.s005.pdf]

Molecular Function	Count	Genes
GO:0000981 sequence-specific DNA binding RNA polymerase II transcription factor activity	96	RARG, THRB, ONECUT2, HNF4G, ZBTB20, AHR, CTCF, MEOX2, NR3C1, BACH1, SOX21, BACH2, CHCHD3, HEY1, CREB3L1, SIX3, MYBL2, SOX9, PITX2, SOX6, PITX1, TP63, SOX4, KLF11, MED1, KLF12, FOXD1, PAX3, PAX6, MITF, HNF1B, SOX11, SOX12, PROX1, FOXP4, PAX2, FOXP2, ETV6, AR, ELF1, NR5A2, CREB1, AEBP2, TAL1, HAND2, PAX9, ERF, RARB, PGR, ERG, PPARA, CLOCK, ZSCAN29, DLX3, NFIX, PLAG1, GATA6, GATA3, NEUROD1, NEUROD2, MNT, NEUROD6, PLAGL2, OTX1, MEF2D, E2F7, ESRRA, TFAP2A, ZFHX3, BCL11B, FOXF1, BCL11A, FOXJ2, NR1D2, ESRRG, ARID3A, NR2F2, ARID3B, NR1D1, KLF4, MEIS2, FLI1, POU4F2, NR4A2, MEIS1, NR6A1, BCL6, SP1, NFIB, SNAI1, SNAI2, TARDBP, RCOR1, FOXA2, NFE2L2, GZF1
GO:0000976 transcription regulatory region sequence-specific DNA binding	87	SUV39H2, RARG, ONECUT2, CTCF, MEOX2, NR3C1, BACH1, GLI3, BACH2, SIX3, MYBL2, SOX9, PITX2, PITX1, JUNB, SOX4, KLF10, MED1, KLF12, FOXD1, SOX11, PAX6, MITF, TBR1, SOX12, PROX1, FOXP4, PAX2, FOXP2, ETV6, AR, ELF1, CREB1, NR5A2, AEBP2, TAL1, HAND2, PAX9, HOXB3, RARB, PGR, TOP1, PPARA, CLOCK, SMARCD2, PLAG1, GATA6, GATA3, NEUROD1, NEUROD2, MNT, MTA1, NEUROD6, NRIP1, OTX1, ZNF148, MEF2D, E2F7, TFAP2A, ESRRA, SMAD1, BCL11B, NFYA, FOXF1, BCL11A, FOXJ2, ESRRG, ARID3A, NR1D2, NR1D1, ARID3B, KLF4, MEIS2, FLI1, POU4F2, PER2, NR4A2, MEIS1, NR6A1, BCL6, SP1, NFIB, SNAI1, HNRNPC, FOXA2, GZF1, NFE2L2
GO:0001012 RNA polymerase II regulatory region DNA binding	75	RARG, ONECUT2, CTCF, MEOX2, NR3C1, BACH1, GLI3, BACH2, SIX3, MYBL2, PITX2, PITX1, JUNB, NCOA1, MED1, KLF12, FOXD1, SOX11, PAX6, MITF, TBR1, FOXP4, PAX2, FOXP2, ETV6, NCOR2, AR, ELF1, CREB1, NR5A2, AEBP2, TAL1, HAND2, PAX9, HOXB3, RARB, PGR, PPARA, CLOCK, SMARCD2, PLAG1, GATA6, GATA3, NEUROD1, NEUROD2, MNT, NEUROD6, OTX1, MEF2D, E2F7, TFAP2A, ESRRA, SMAD1, BCL11B, FOXF1, BCL11A, FOXJ2, ESRRG, ARID3A, NR1D1, ARID3B, MEIS2, FLI1, POU4F2, NR4A2, MEIS1, NR6A1, BCL6, SP1, NFIB, SNAI1, HNRNPC, FOXA2, NFE2L2, GZF1
GO:0000977 RNA polymerase II regulatory region sequence-specific DNA binding	73	RARG, ONECUT2, CTCF, MEOX2, NR3C1, BACH1, GLI3, BACH2, SIX3, MYBL2, PITX2, JUNB, PITX1, MED1, KLF12, FOXD1, SOX11, PAX6, MITF, TBR1, FOXP4, PAX2, FOXP2, ETV6, AR, ELF1, CREB1, NR5A2, AEBP2, TAL1, HAND2, PAX9, HOXB3, RARB, PGR, PPARA, CLOCK, SMARCD2, PLAG1, GATA6, GATA3, NEUROD1, NEUROD2, MNT, NEUROD6, OTX1, MEF2D, E2F7, TFAP2A, ESRRA, SMAD1, BCL11B, FOXF1, BCL11A, FOXJ2, ESRRG, ARID3A, ARID3B, NR1D1, MEIS2, FLI1, POU4F2, NR4A2, MEIS1, NR6A1, BCL6, SP1, NFIB, SNAI1, HNRNPC, FOXA2, NFE2L2, GZF1
GO:0000982 RNA polymerase II core promoter proximal region sequence-specific DNA binding transcription factor activity	57	ONECUT2, ZBTB20, CTCF, MEOX2, NR3C1, BACH1, CREB3L1, MYBL2, SOX9, PITX2, PITX1, TP63, SOX4, FOXD1, MITF, SOX11, PAX6, SOX12, FOXP4, FOXP2, PAX2, ETV6, AR, ELF1, CREB1, NR5A2, AEBP2, HAND2, PGR, PPARA, CLOCK, DLX3, PLAG1, GATA3, NEUROD1, NEUROD6, PLAGL2, OTX1, TFAP2A, ESRRA, BCL11B, BCL11A, FOXJ2, NR1D1, ARID3B, KLF4, MEIS2, FLI1, POU4F2, MEIS1, NR6A1, SP1, NFIB, SNAI1, SNAI2, RCOR1, GZF1

Table S5: Most significant Gene Ontology terms (Molecular Function) associated with miRNA targeted genes for BRCA.

Molecular Function	Count	Genes
GO:0000981 sequence-specific DNA binding RNA polymerase II transcription factor activity	121	ATF2, EHF, ZNF292, RORB, AHR, RBPJ, NR3C1, BACH2, ETS2, YY1, HOXA10, DACH1, MYB, CREB3L2, PKNOX1, PITX2, SOX6, SOX4, TLE4, MEF2C, RFX3, SOX11, SOX12, OVOL1, HCFC1, FOXP1, SUB1, ERG, CLOCK, DLX5, EPAS1, MAZ, GATA6, GLIS3, GATA3, PHB, HIF1A, FOXO1, GLIS2, NEUROD1, ZKSCAN2, EGR1, TFAP2B, JUN, TFAP2C, CREBBP, ZFHX3, BCL11B, BCL11A, NR1D2, ESRRG, NR2F2, ESR1, NFATC4, NR4A2, NR4A3, SP1, FUBP1, SP3, SNAI2, MKX, MXD1, RCOR1, MYF5, NFE2L2, BTG2, ZNF131, THRB, ONECUT2, HNF4G, MEOX2, HEY2, MLX, MYBL2, MYBL1, GABPA, ZNF641, KLF12, ARNT, PAX6, ETV1, ETV4, ETV5, ETV6, CREB3, CREB1, ELF2, AEBP2, HAND2, MYOD1, MTF1, RARB, CREB5, PPARD, FOXC1, CEBPB, NFIX, PLAG1, ZNF518B, NR1I2
GO:0000982 RNA polymerase II core promoter proximal region sequence-specific DNA binding transcription factor activity	75	ATF2, EHF, BTG2, ONECUT2, ZNF292, RBPJ, MEOX2, NR3C1, ETS2, YY1, HOXA10, DACH1, MYB, CREB3L2, MYBL2, PITX2, MYBL1, GABPA, SOX4, MEF2C, SOX11, PAX6, ETV1, SOX12, OVOL1, ETV4, ETV6, CREB3, CREB1, AEBP2, MTF1, HAND2, CLOCK, CREB5, CEBPB, DLX5, EPAS1, PLAG1, GLIS3, FOXK2, GATA3, HIF1A, FOXO1, GLIS2, ARNTL, NEUROD1, NFIL3, PLAGL2, HES1, MSX1, HES5, EGR1, TFAP2B, JUN, CREBBP, TFAP2C, BCL11B, BCL11A, NR1H2, FOXJ2, KLF4, ESR1, POU4F2, NFATC4, MEIS1, TFAP4, NFIA, WT1, SP1, NFIB, SP3, SNAI2, MKX, MXD1, RCOR1
GO:0000976 transcription regulatory region sequence-specific DNA binding	97	ATF2, EHF, RBPJ, NR3C1, ETS2, BACH2, HOXA10, MYB, CREB3L2, EP300, PITX2, SOX4, SMARCC1, MEF2C, LMO4, SOX11, SOX12, OVOL1, ISL1, HOXB3, CLOCK, DLX5, MAZ, GATA6, GLIS3, GATA3, NEUROD1, ZNF148, EGR1, TFAP2B, JUN, TFAP2C, CREBBP, BCL11B, BCL11A, H3F3A, ESRRG, NR1D2, SMAD5, ESR1, FOSL2, NFATC4, NR4A2, SP1, SP3, MKX, MXD1, NFE2L2, ONECUT2, MEOX2, CHD2, GLI3, MLX, MYBL2, UTY, MYBL1, GABPA, KLF10, KLF12, MKL1, PAX6, ETV1, FOS, ETV4, ETV5, ETV6, CREB3, SFPQ, CREB1, AEBP2, HAND2, MYOD1, MTF1, RARB, TOP1, FOXC1, PLAG1, NR1I2, FOXK2, ARNTL, NFIL3, HSF2, NRIP1, MSX1, MEF2D, E2F7, NFYA, NR1H2, FOXJ2, KLF4, GATAD2B, POU4F2, PER1, MEIS1, TFAP4, NFIA, NFIB
GO:0008134 transcription factor binding	122	TCERG1, ATF2, JMJD1C, AHR, RORB, RBPJ, IKZF4, RNF19A, ENPP2, EP300, DIP2B, PITX2, DIP2C, HMGN3, TLE4, MEF2C, LMO4, KCTD1, ISL1, RUNX2, RUNX1, ZFPM2, TRIB2, BEX1, INSIG2, EPAS1, PRKDC, GATA6, GATA3, FOXO4, STK4, NLK, HIF1A, MED12L, MTDH, NEUROD1, NHLH2, TP53BP2, PPARGC1A, BPTF, NKX2-2, SMAD2, EGR1, CREBBP, EGR2, JUN, DR1, PHF12, ESR1, NFATC4, MAFB, SP1, CNOT1, CNOT2, HNRNPF, ID4, TAF4B, MKX, NFE2L2, PPP1R13B, DDX3X, CITED2, CHD6, MECP2, MED14, MED13, HEY2, MLX, TEAD3, NCOA1, NCOA6, TSC22D3, ANXA4, NCOA3, TCF12, ARNT, PAX6, FOS, SIRT1, EPN1, NCOR2, CREB1, TFDP1, HAND2, MYOD1, CRY1, LCOR, RARB, PPARD, HDAC4, GTF2A2, CEBPB, FOXC1, CEBPG, ASCL1, RNF4, HDAC9, ARNTL, PURB, PURA
GO:0001228 RNA polymerase II transcription regulatory region sequence-specific DNA binding transcription factor activity involved in positive regulation of transcription	68	ATF2, EHF, BTG2, ONECUT2, ZNF292, RBPJ, NR3C1, MEOX2, HOXA10, MYB, CREB3L2, MYBL2, PITX2, MYBL1, GABPA, SOX4, MEF2C, SOX11, PAX6, ETV1, SOX12, ETV4, ETV5, ETV6, HCFC1, CREB3, CREB1, MTF1, HAND2, MYOD1, SUB1, CREB5, FOXC1, CEBPB, DLX5, EPAS1, PLAG1, NR1I2, GLIS3, FOXK2, GATA3, HIF1A, GLIS2, NEUROD1, PLAGL2, MSX1, EGR1, TFAP2B, TFAP2C, JUN, BCL11B, NR1H2, FOXJ2, ESRRG, KLF4, ESR1, POU4F2, NR4A2, MEIS1, TFAP4, NFIA, WT1, SP1, NFIB, FUBP1, MKX, TARDBP, NFE2L2

Table S5: Most significant Gene Ontology terms (Molecular Function) associated with miRNA targeted genes for KIRC.

Molecular Function	Count	Genes
GO:0000981 sequence-specific DNA binding RNA polymerase II transcription factor activity	125	EHF, SPI1, GFI1, RORC, RORB, RBPJ, BACH1, ETS1, BACH2, HOXA10, SOX2, DACH1, MYB, CREB3L2, SOX8, PITX2, SOX6, TP63, SOX4, SOX5, TLE4, MEF2A, MED1, MEF2C, MITF, SOX11, EBF4, FOXP1, ERG, TP53, CLOCK, ATF3, EPAS1, GLIS3, GATA2, HIF1A, FOXO1, GLIS2, NEUROD1, SCRT2, ZKSCAN2, OTX2, ALX1, OTX1, ESRRA, ZFHX3, TFAP2D, BCL11B, FOXF1, BCL11A, NFATC3, HMGA2, NR1D2, ESRRG, ESR1, POU2F3, NFATC4, NR4A2, HNRNPK, NR4A3, SP1, FUBP1, SP3, TCF3, MKX, MXD1, RCOR1, RARG, BTG2, THRB, THRA, ONECUT2, FOXE1, NR2E1, MEOX2, HOXC11, HEY2, MLX, MYBL1, GABPA, NKX3-1, KLF12, PAX3, ARNT, ETV1, PAX2, POU5F1, ELF2, AEBP2, ELF4, ELF5, MTF1, RARB, PPARA, CREB5, CEBPB, NFIX, PLAG1, PTH, ZNF518B
GO:0000982 RNA polymerase II core promoter proximal region sequence-specific DNA binding transcription factor activity	76	EHF, BTG2, SPI1, ONECUT2, GFI1, NR2E1, RBPJ, MEOX2, BACH1, HOXC11, HOXA10, SOX2, DACH1, MYB, CREB3L2, PITX2, MYBL1, TP63, GABPA, SOX4, MEF2A, MEF2C, MITF, SOX11, ETV1, PAX2, AEBP2, ELF4, MTF1, PPARA, CLOCK, TP53, ATF3, CREB5, CEBPB, EPAS1, PLAG1, SRF, GLIS3, FOXK2, GATA2, HIF1A, RELA, FOXO1, GLIS2, NEUROD1, SCRT2, PLAGL2, OTX2, OTX1, ESRRA, BCL11B, BCL11A, NFATC3, HMGA2, ARID3B, MEIS3, KLF4, MEIS2, ESR1, POU2F3, POU4F2, NFATC4, CTCFL, MEIS1, TFAP4, HNRNPK, KLF5, NFIA, SP1, NFIB, SP3, TCF3, MKX, MXD1, RCOR1
GO:0000976 transcription regulatory region sequence-specific DNA binding	106	EHF, SPI1, RBPJ, BACH1, BACH2, HOXA10, SOX2, SOX1, MYB, CREB3L2, DPF2, EP300, SOX8, PITX2, SOX4, MEF2A, MED1, SMARCC1, MEF2C, SOX11, MITF, EBF4, ISL1, TP53, CLOCK, ATF3, ZNF431, GLIS3, GATA2, NEUROD1, OTX2, ALX1, OTX1, ESRRA, SMAD1, SMAD3, TFAP2D, BCL11B, FOXF1, BCL11A, H3F3A, NFATC3, ESRRG, NR1D2, SMAD5, ESR1, POU2F3, FOSL2, NFATC4, SMARCA4, NR4A2, HNRNPK, SP1, SP3, MKX, TCF3, MXD1, RARG, ONECUT2, CHD4, MEOX2, CHD2, HOXC11, MLX, MYBL1, GABPA, NKX3-1, KLF12, ETV1, FOS, PAX2, SREBF2, AEBP2, ELF4, ELF5, MTF1, CRY2, RARB, PPARA, SMARCD2, UHRF1, PLAG1, SRF, FOXK2, RELA, MNT, RXRA, CUX1, NRIP1, MTA2, MEF2D, E2F7, ARID3A, ARID3B, TBX5, KLF4, MEIS2, GATAD2B, POU4F2, CTCFL
GO:0001012 RNA polymerase II regulatory region DNA binding	94	EHF, RARG, SPI1, ONECUT2, CHD4, MEOX2, RBPJ, BACH1, HOXC11, BACH2, HOXA10, MYB, CREB3L2, DPF2, MLX, EP300, SOX8, PITX2, MYBL1, GABPA, NCOA1, MEF2A, MED1, SMARCC1, KLF12, MEF2C, SOX11, MITF, ETV1, FOS, EBF4, PAX2, SREBF2, NCOR2, AEBP2, ELF4, ELF5, MTF1, RARB, PPARA, TP53, CLOCK, ATF3, SMARCD2, PLAG1, SRF, GLIS3, FOXK2, GATA2, RELA, NEUROD1, MNT, RXRA, CUX1, OTX2, ALX1, OTX1, MTA2, MEF2D, E2F7, ESRRA, SMAD1, TFAP2D, BCL11B, FOXF1, BCL11A, H3F3A, NFATC3, ESRRG, ARID3A, ARID3B, TBX5, MEIS2, SMAD5, ESR1, POU2F3, GATAD2B, POU4F2, FOSL2, NFATC4, SMARCA4, CTCFL, NR4A2, MEIS1, TFAP4, KLF5, HNRNPK, NFIA, SP1, NFIB, SP3, MKX, TCF3, MXD1
GO:0000977 RNA polymerase II regulatory region sequence-specific DNA binding	92	EHF, RARG, SPI1, ONECUT2, CHD4, MEOX2, RBPJ, BACH1, HOXC11, BACH2, HOXA10, MYB, CREB3L2, DPF2, MLX, EP300, SOX8, PITX2, MYBL1, GABPA, MEF2A, MED1, SMARCC1, KLF12, MEF2C, SOX11, MITF, ETV1, FOS, EBF4, PAX2, SREBF2, AEBP2, ELF4, MTF1, ELF5, RARB, PPARA, TP53, CLOCK, ATF3, SMARCD2, PLAG1, SRF, GLIS3, FOXK2, GATA2, RELA, NEUROD1, MNT, RXRA, CUX1, OTX2, ALX1, OTX1, MTA2, MEF2D, E2F7, ESRRA, SMAD1, TFAP2D, BCL11B, FOXF1, BCL11A, H3F3A, NFATC3, ESRRG, ARID3A, ARID3B, TBX5, MEIS2, SMAD5, ESR1, POU2F3, GATAD2B, POU4F2, SMARCA4, FOSL2, NFATC4, CTCFL, NR4A2, MEIS1, TFAP4, KLF5, HNRNPK, NFIA, SP1, NFIB, SP3, TCF3, MKX, MXD1

Table S5: Most significant Gene Ontology terms (Molecular Function) associated with miRNA targeted genes for LGG.

Molecular Function	Count	Genes
GO:0000981 sequence-specific DNA binding RNA polymerase II transcription factor activity	87	FOXA1, RARG, THRA, ONECUT2, RORC, HNF4G, ETS1, BACH2, ELK3, YY1, MYC, HNF4A, CREB3L1, MYB, PKNOX1, SOX6, ZNF202, ZNF641, NKX3-2, SOX4, NKX3-1, TLE4, KLF11, MED1, KLF12, ARNT, MITF, ETV1, SOX11, RUNX3, FOXP2, ETV6, FOXP1, NR5A2, AEBP2, TAL1, MTF1, RARB, PPARA, CLOCK, CREB5, PPARD, NFIX, PLAG1, EPAS1, PTH, PTGER3, LEF1, NR1I2, GATA6, GLIS3, GATA3, HIF1A, NEUROD1, NEUROD2, PURA, SCRT2, PLAGL1, NHLH1, PLAGL2, OTX1, MEF2D, E2F7, ESRRA, TFAP2A, TFAP2C, BCL11B, FOXF1, STAT3, FOXJ2, HMGA2, ESRRG, TBX5, KLF4, ESR1, USF2, USF1, CTCFL, NFATC4, NR4A2, HNRNPK, NR4A3, BCL6, NFIB, MXD1, CC2D1A, MYF5
GO:0004674 protein serine/threonine kinase activity	91	BMPR2, ZAK, LRRK1, RPS6KA4, RPS6KA3, AKAP13, STK11, RPS6KA5, RPS6KA2, FASTK, RPS6KA1, AKT3, PBK, MAP3K8, MAP3K9, STK32B, MAP3K7, PRKACB, MAP3K5, MAP2K3, CSNK1G3, MAP2K1, PRKCH, CHUK, PRKCB, PRKCE, MINK1, LMTK3, DYRK1A, HUNK, OXSR1, MASTL, TGFBR2, CIT, LATS1, PIK3CA, PRKD2, PRKCQ, ULK1, PRKD1, SQSTM1, CSNK1G1, CAMK2D, CAB39, ROCK2, ACVR1B, NLK, LTBP1, TTN, MAPK9, NUAK1, STK33, PAK1, STK35, STK38, MKNK2, GRK6, PAK7, STK38L, MAPK1, MARK4, CAMK2G, PAK2, TRPM6, MAPK4, MAP4K4, SRPK2, MAP3K2, NEK9, MAP3K3, MAP3K1, LIMK1, HMGA2, GTF2H1, MAPK14, ACVR2B, SRPK1, WEE1, CDK6, RPS6KB1, TAOK3, STK17A, STK17B, TAOK2, WNK3, FOSB, PKN2, MAP3K14, MAP3K11, MAP3K12, CDK5R1
GO:0001012 RNA polymerase II regulatory region DNA binding	67	RARG, ONECUT2, BACH2, ELK3, MYC, HNF4A, MYB, DPF2, EP300, NKX3-2, NCOA1, MED1, SMARCC1, KLF12, SOX11, MITF, ETV1, RUNX3, FOXP2, ETV6, NCOR2, NR5A2, PAX8, AEBP2, TAL1, IRF1, MTF1, HOXB3, RARB, PPARA, CLOCK, PLAG1, LEF1, NR1I2, GATA6, GLIS3, GATA3, NEUROD1, NEUROD2, PLAGL1, NHLH1, OTX1, MTA2, MEF2D, E2F7, TFAP2A, ESRRA, SMAD1, TFAP2C, BCL11B, FOXF1, FOXJ2, ESRRG, TBX5, SMAD5, ESR1, USF2, GATAD2B, NFATC4, CTCFL, FOSL1, NR4A2, HNRNPK, BCL6, NFIB, MXD1, CC2D1A
GO:0000976 transcription regulatory region sequence-specific DNA binding	74	RARG, ONECUT2, CHD2, BACH2, ELK3, SIN3A, MYC, HNF4A, MYB, DPF2, EP300, NKX3-2, SOX4, NKX3-1, KLF10, MED1, SMARCC1, KLF12, SOX11, MITF, ETV1, RUNX3, FOXP2, ETV6, NR5A2, PAX8, AEBP2, TAL1, IRF1, MTF1, CRY2, HOXB3, RARB, PPARA, CLOCK, PLAG1, LEF1, NR1I2, GATA6, GLIS3, GATA3, NEUROD1, NEUROD2, PLAGL1, NHLH1, NRIP1, OTX1, ZNF148, MTA2, MEF2D, E2F7, TFAP2A, ESRRA, SMAD1, TFAP2C, BCL11B, FOXF1, FOXJ2, ESRRG, TBX5, KLF4, SMAD5, ESR1, USF2, GATAD2B, NFATC4, CTCFL, FOSL1, NR4A2, HNRNPK, BCL6, NFIB, MXD1, CC2D1A
GO:0000977 RNA polymerase II regulatory region sequence-specific DNA binding	65	RARG, ONECUT2, BACH2, ELK3, MYC, HNF4A, MYB, DPF2, EP300, NKX3-2, MED1, SMARCC1, KLF12, SOX11, MITF, ETV1, RUNX3, FOXP2, ETV6, NR5A2, PAX8, AEBP2, TAL1, IRF1, MTF1, HOXB3, RARB, PPARA, CLOCK, PLAG1, LEF1, NR1I2, GATA6, GLIS3, GATA3, NEUROD1, NEUROD2, PLAGL1, NHLH1, OTX1, MTA2, MEF2D, E2F7, TFAP2A, ESRRA, SMAD1, TFAP2C, BCL11B, FOXF1, FOXJ2, ESRRG, TBX5, SMAD5, ESR1, USF2, GATAD2B, NFATC4, CTCFL, FOSL1, NR4A2, HNRNPK, BCL6, NFIB, MXD1, CC2D1A

Table S5: Most significant Gene Ontology terms (Molecular Function) associated with miRNA targeted genes for LIHC.

Molecular Function	Count	Genes
GO:0000976 transcription regulatory region sequence-specific DNA binding	117	EHF, NR3C1, ELK1, BACH1, BACH2, HOXA10, SOX2, MYB, DPF2, EP300, SOX9, PITX2, SOX4, MED1, SMARCC1, SMARCC2, LMO4, SOX11, MITF, TBR1, FOXP4, ISL1, FOXP2, ATF5, TP53, CLOCK, HOXB5, MAZ, GATA6, GATA3, NEUROD2, OTX1, ZNF148, TFAP2A, ESRRA, STAT5A, TFAP2B, STAT5B, JUN, TFAP2C, SMAD3, TFAP2D, BCL11B, BCL11A, NFATC3, ESRRG, NR1D2, NR1D1, SMAD5, ESR1, NFKB1, FOSL2, FOSL1, SP1, MKX, TCF3, MXD1, NFE2L2, RARG, ONECUT2, CHD4, CTCF, MEOX2, CHD2, HOXC11, GLI3, MYBL1, GABPA, KLF12, H2AFZ, ETV1, FOS, PAX5, PROX1, PAX2, ETV5, NME1, SFPQ, CREB1, AEBP2, TAL1, ELF4, HAND2, PAX9, CRY2, RARB, TOP1, PPARA, SMARCD2, FOXC1, PLAG1, WBP2, SRF, ARNTL, MNT, CUX2, RXRA, CUX1, PLAGL1, NRIP1
GO:0001012 RNA polymerase II regulatory region DNA binding	101	EHF, NR3C1, BACH1, ELK1, BACH2, HOXA10, MYB, DPF2, EP300, PITX2, MED1, SMARCC1, SMARCC2, SOX11, MITF, TBR1, FOXP4, FOXP2, ATF5, TP53, CLOCK, HOXB5, MAZ, GATA6, GATA3, NEUROD2, OTX1, TFAP2A, ESRRA, STAT5A, TFAP2B, STAT5B, JUN, TFAP2C, TFAP2D, BCL11B, BCL11A, NFATC3, ESRRG, NR1D1, SMAD5, ESR1, FOSL2, FOSL1, SP1, MKX, TCF3, MXD1, NFE2L2, RARG, ONECUT2, CHD4, CTCF, MEOX2, HOXC11, GLI3, MYBL1, GABPA, NCOA1, KLF12, H2AFZ, ETV1, FOS, PAX5, PAX2, ETV5, NME1, NCOR2, CREB1, AEBP2, TAL1, ELF4, HAND2, PAX9, RARB, PPARA, SMARCD2, FOXC1, PLAG1, WBP2, SRF, MNT, CUX2, RXRA, CUX1, PLAGL1, MTA2, MEF2D, E2F7, ARID3A, ARID3B, TBX5, MEIS2, POU4F1, POU4F2, MEIS1, TFAP4, KLF5, NFIB, ZNF335
GO:0000981 sequence-specific DNA binding RNA polymerase II transcription factor activity	125	FOXA1, EHF, RORC, NR3C1, BACH1, ELK1, BACH2, YY1, HOXA10, SOX2, DACH1, SOX17, CREB3L1, MYB, SOX9, PITX2, TP63, SOX4, SOX5, TLE4, MED1, MITF, SOX11, RFX1, FOXP4, FOXP2, FOXP1, ERG, ATF5, TP53, CLOCK, HOXB5, MAZ, GATA6, GATA3, FOXO1, NEUROD2, SCRT2, ZKSCAN2, OTX1, ESRRA, TFAP2A, TFAP2B, JUN, TFAP2C, ZFHX3, TFAP2D, BCL11B, BCL11A, NFATC3, HMGA2, NR1D2, ESRRG, NR1D1, ESR1, SOX30, SP1, FUBP1, TCF3, MKX, MXD1, BMPR1A, NFE2L2, RARG, BTG2, THRB, THRA, ONECUT2, HOXC13, CTCF, MEOX2, HOXC11, HEY1, HEY2, MYBL1, GABPA, TEAD2, ZNF641, KLF12, PAX3, ARNT, ETV1, PAX5, PROX1, PAX2, ETV5, CREB1, ELF2, AEBP2, TAL1, ELF4, HAND2, PAX9, RARB, PPARA, CREB5, FOXC1, NFIX, PLAG1, PTH
GO:0000977 RNA polymerase II regulatory region sequence-specific DNA binding	99	EHF, NR3C1, BACH1, ELK1, BACH2, HOXA10, MYB, DPF2, EP300, PITX2, MED1, SMARCC1, SMARCC2, SOX11, MITF, TBR1, FOXP4, FOXP2, ATF5, TP53, CLOCK, HOXB5, MAZ, GATA6, GATA3, NEUROD2, OTX1, TFAP2A, ESRRA, STAT5A, TFAP2B, STAT5B, JUN, TFAP2C, TFAP2D, BCL11B, BCL11A, NFATC3, ESRRG, NR1D1, SMAD5, ESR1, FOSL2, FOSL1, SP1, TCF3, MKX, MXD1, NFE2L2, RARG, ONECUT2, CHD4, CTCF, MEOX2, HOXC11, GLI3, MYBL1, GABPA, KLF12, H2AFZ, ETV1, FOS, PAX5, PAX2, ETV5, NME1, CREB1, AEBP2, TAL1, ELF4, HAND2, PAX9, RARB, PPARA, SMARCD2, FOXC1, PLAG1, WBP2, SRF, MNT, CUX2, RXRA, CUX1, PLAGL1, MTA2, MEF2D, E2F7, ARID3A, ARID3B, TBX5, MEIS2, POU4F1, POU4F2, MEIS1, TFAP4, KLF5, NFIB, ZNF335, FOXA2
GO:0000982 RNA polymerase II core promoter proximal region sequence-specific DNA binding transcription factor activity	71	FOXA1, EHF, BTG2, ONECUT2, CTCF, HOXC13, MEOX2, NR3C1, ELK1, BACH1, HOXC11, YY1, HOXA10, SOX2, DACH1, CREB3L1, MYB, SOX9, PITX2, MYBL1, TP63, GABPA, SOX4, MITF, SOX11, ETV1, PAX5, FOXP4, FOXP2, PAX2, CREB1, AEBP2, ELF4, HAND2, PPARA, CLOCK, TP53, CREB5, PLAG1, SRF, GATA3, FOXO1, ARNTL, SCRT2, PLAGL2, OTX1, TFAP2A, ESRRA, TFAP2B, JUN, TFAP2C, BCL11B, BCL11A, NFATC3, HMGA2, NR1D1, ARID3B, MEIS3, MEIS2, ESR1, POU4F1, POU4F2, MEIS1, TFAP4, KLF5, WT1, SP1, NFIB, TCF3, MKX, MXD1

Table S5: Most significant Gene Ontology terms (Molecular Function) associated with miRNA targeted genes for LUAD.

Molecular Function	Count	Genes
GO:0004674 protein serine/threonine kinase activity	106	ZAK, NRK, TESK2, STK19, MYLK3, MYLK, RPS6KA3, CCND3, RPS6KA5, CHEK1, AKT3, PDK4, PIM1, PIM3, MAP3K9, PRKACA, MAP3K7, PRKACB, MAP3K4, PSKH1, MAP3K5, PDK1, CDKL2, ACVR1, MAP2K4, PRKAB2, MAP2K1, PRKCE, LMTK2, DYRK1B, BCR, PRKD3, MAK, ULK2, RAF1, CSNK1G1, LTBP1, PRKCZ, KALRN, NUAK1, STK33, STK38, STK38L, MARK1, HMGA2, DCLK1, SRPK1, CDK6, WNK1, WNK3, AAK1, MAP3K14, MAP3K12, DYRK2, BMPR2, LRRK2, LRRK1, AATK, IKBKB, AKAP13, FASTK, TLK1, PRKG1, CAMK1D, KSR1, CHUK, MINK1, MASTL, TGFBR1, TGFBR2, CIT, TGFBR3, LATS2, PDIK1L, SGK1, PRKAA1, PRKAA2, ROCK1, CAB39, CAMK2A, TTK, TTN, MAPK9, PAK1, MAPK8, MKNK1, PAK7, PAK6, MAPK1, CAMK2G, PAK2, MAP4K3, MAP3K3, MAP3K1, GTF2H1, MAPK14, ACVR2B, ACVR2A, FKBP1A, MAPK10
GO:0000981 sequence-specific DNA binding RNA polymerase II transcription factor activity	94	RARG, BTG2, THRB, ONECUT2, ZNF292, HNF4G, MEOX2, NR3C1, HOXC11, SOX21, BACH2, ELK3, HOXA10, DACH1, HEY2, MYB, SOX9, MYBL1, GABPA, TP63, NKX3-2, SOX4, SOX5, TLE4, KLF11, MED1, KLF12, PAX3, ARNT, MITF, ETV1, SOX11, SOX12, KLF15, RUNX3, PAX2, ETV5, FOXP2, FOXP1, ELF1, NR5A2, AEBP2, MTF1, IRF8, RARB, PPARG, ERG, PPARA, CLOCK, CREB5, FOXC1, DLX5, PLAG1, EPAS1, CEBPE, PTH, ZNF518B, SRF, TWIST1, HIF1A, FOXO1, NEUROD1, PURA, MNT, RXRA, ZKSCAN2, OTX1, E2F7, E2F8, ESRRA, ZFHX3, TFAP2D, FOXF2, BCL11B, BCL11A, NFATC3, HMGA2, NR1D2, ESRRG, ESR1, POU4F1, POU4F2, NFATC4, MEIS1, TFAP4, NFIA, SP1, NFIB, FUBP1, TCF3, MKX, TARDBP, MXD1, NFE2L2
GO:0000976 transcription regulatory region sequence-specific DNA binding	81	RARG, ONECUT2, SUV39H1, MEOX2, CHD2, NR3C1, HOXC11, BACH2, ELK3, HOXA10, MYB, EP300, SOX9, MYBL1, GABPA, NKX3-2, SOX4, MED1, KLF12, SOX11, MITF, ETV1, SOX12, RUNX3, MYT1, PAX2, ETV5, FOXP2, SFPQ, ELF1, NR5A2, AEBP2, IRF1, MTF1, HOXB3, RARB, TOP1, PPARA, CLOCK, SMARCD2, FOXC1, PLAG1, DLX5, CEBPE, SRF, NEUROD1, MNT, RXRA, NRIP1, OTX1, ZNF148, E2F7, E2F8, ESRRA, SMAD1, SMAD3, TFAP2D, FOXF2, BCL11B, BCL11A, NFATC3, ESRRG, NR1D2, SMAD5, ESR1, POU4F1, GATAD2B, POU4F2, FOSL2, NFATC4, SMARCA4, FOSL1, MEIS1, TFAP4, NFIA, SP1, NFIB, MKX, TCF3, MXD1, NFE2L2
GO:0001012 RNA polymerase II regulatory region DNA binding	72	RARG, ONECUT2, MEOX2, NR3C1, HOXC11, BACH2, ELK3, HOXA10, MYB, EP300, MYBL1, GABPA, NKX3-2, NCOA1, MED1, KLF12, SOX11, MITF, ETV1, RUNX3, PAX2, ETV5, FOXP2, NCOR2, ELF1, NR5A2, AEBP2, IRF1, MTF1, HOXB3, RARB, PPARG, PPARA, CLOCK, SMARCD2, FOXC1, PLAG1, DLX5, CEBPE, SRF, NEUROD1, MNT, RXRA, OTX1, E2F7, E2F8, ESRRA, SMAD1, TFAP2D, FOXF2, BCL11B, BCL11A, NFATC3, ESRRG, SMAD5, ESR1, POU4F1, GATAD2B, POU4F2, FOSL2, NFATC4, SMARCA4, FOSL1, MEIS1, TFAP4, NFIA, SP1, NFIB, MKX, TCF3, MXD1, NFE2L2
GO:0019900 kinase binding	102	RB1, CCNT2, IRS1, MAML1, WWC1, DIXDC1, IRS2, LIPE, RPS6KA3, CCND3, CCND2, CCND1, DPYSL2, SOX9, PRKACA, MAP3K5, NSF, MAP2K4, CASR, MAP2K1, ANK2, ACSL3, CDC25A, CDC25B, TOM1L1, CCNE1, ADAM9, RAF1, STXBP1, IQGAP1, HIF1A, ZBTB4, PRKCZ, BAG5, RACGAP1, PRKAR2A, DVL1, STK38, PLCG1, RAB11FIP2, BARD1, SMAD1, SMAD3, PTK2, GFAP, CCDC88A, CYLD, NBEA, GRB2, TCF3, PAM, MAP3K12, RYR2, PTPRR, BTG1, CLTC, PDE3B, PTEN, SLC2A1, TNFAIP3, LDB3, AP2A1, PTPRK, CD3E, GHR, IKBKB, SPRED1, RB1CC1, APPL1, KSR1, PGAM1, PPP2R5A, AXIN2, YWHAZ, ADRA2A, TGFBR2, DCX, CRY1, HDAC4, PRKAA1, STX17, CAB39, AKAP7, JAKMIP1, TTN, MAPK9, PAK1, RNF138, UBQLN1, MAPK1, RICTOR, PDLIM5, PAK2, SASH1, SPAG9, MAP3K1, SLC12A5, DAB2IP, APC, NBR1

Table S5: Most significant Gene Ontology terms (Molecular Function) associated with miRNA targeted genes for PAAD.

Molecular Function	Count	Genes
GO:0000981 sequence-specific DNA binding RNA polymerase II transcription factor activity	83	FOXA1, EHF, BTG2, THRB, THRA, ONECUT2, HNF4G, TCF21, CTCF, HOXC11, ELK1, SPIB, HOXA10, SOX2, DACH1, HEY2, MYB, SOX6, MYBL1, TP63, SOX5, MEF2A, KLF12, MEF2C, SOX11, FOXP4, FOXP2, NR5A2, CREB1, ELF2, PAX9, RARA, RARB, PPARG, ERG, PPARA, TP53, CLOCK, ATF3, HOXB5, NFIX, PLAG1, SRF, PTGER3, GATA6, GATA3, PHB, NRF1, GATA2, FOXO1, NEUROD2, MNT, RXRA, NFIL3, NEUROD6, PLAGL1, ZKSCAN2, PLAGL2, OTX2, MEF2D, E2F7, EGR1, TFAP2B, ZFHX3, BCL11A, NR1D2, ARID3A, NR2F2, TBX5, MEIS2, POU2F3, NR2F6, MEIS1, NR4A3, SP1, NFIB, FUBP1, SP3, SNAI1, TARDBP, BMPR1A, NFE2L2, GZF1
GO:0001012 RNA polymerase II regulatory region DNA binding	68	EHF, ONECUT2, CTCF, HOXC11, ELK1, HOXA10, RUVBL2, MYB, EP300, MYBL1, NCOA1, MEF2A, KLF12, MEF2C, SMARCC2, H2AFZ, SOX11, TBR1, FOXP4, FOXP2, NME1, CREB1, NR5A2, PAX9, RARA, HOXB3, RARB, PPARG, PPARA, TP53, CLOCK, ATF3, HOXB5, PLAG1, SRF, GATA6, GATA3, NRF1, GATA2, NEUROD2, MNT, RXRA, NFIL3, NEUROD6, PLAGL1, OTX2, MTA2, MEF2D, E2F7, SMAD1, TFAP2B, EGR1, BCL11A, ARID3A, TBX5, MEIS2, SMAD5, POU2F3, PHOX2B, NR2F6, PER1, MEIS1, SP1, NFIB, SP3, SNAI1, NFE2L2, GZF1
GO:0000976 transcription regulatory region sequence-specific DNA binding	75	EHF, ONECUT2, CTCF, HOXC11, ELK1, HOXA10, SOX2, RUVBL2, MYB, EP300, MYBL1, MEF2A, KLF12, MEF2C, SMARCC2, LMO4, H2AFZ, SOX11, TBR1, FOXP4, ISL1, MYT1, FOXP2, NME1, CREB1, NR5A2, PAX9, RARA, HOXB3, RARB, PPARA, TP53, CLOCK, ATF3, HOXB5, PLAG1, SRF, GATA6, GATA3, NRF1, GATA2, NEUROD2, MNT, RXRA, NFIL3, NEUROD6, PLAGL1, NRIP1, OTX2, ZNF148, MTA2, MEF2D, E2F7, EGR1, SMAD1, TFAP2B, SMAD4, BCL11A, ARID3A, NR1D2, TBX5, MEIS2, SMAD5, POU2F3, PHOX2B, NFKB1, NR2F6, PER1, MEIS1, SP1, NFIB, SP3, SNAI1, GZF1, NFE2L2
GO:0000977 RNA polymerase II regulatory region sequence-specific DNA binding	66	EHF, ONECUT2, CTCF, HOXC11, ELK1, HOXA10, RUVBL2, MYB, EP300, MYBL1, MEF2A, KLF12, MEF2C, SMARCC2, H2AFZ, SOX11, TBR1, FOXP4, FOXP2, NME1, CREB1, NR5A2, PAX9, RARA, HOXB3, RARB, PPARA, TP53, CLOCK, ATF3, HOXB5, PLAG1, SRF, GATA6, GATA3, NRF1, GATA2, NEUROD2, MNT, RXRA, NFIL3, NEUROD6, PLAGL1, OTX2, MTA2, MEF2D, E2F7, SMAD1, TFAP2B, EGR1, BCL11A, ARID3A, TBX5, MEIS2, SMAD5, POU2F3, PHOX2B, NR2F6, PER1, MEIS1, SP1, NFIB, SP3, SNAI1, NFE2L2, GZF1
GO:0004674 protein serine/threonine kinase activity	79	MYLK2, CCNK, BMPR2, ZAK, NRK, RPS6KA5, RPS6KA1, TLK2, PRKG2, MAP3K8, PRKACA, MAP3K4, PDK1, ACVR1, MAP2K4, PRKCI, PDPK1, PRKCE, DAPK2, PRKCD, MINK1, DYRK1A, HUNK, CASK, CSNK1D, TGFBR1, CIT, MAPKAPK3, PRKD3, SGK1, STK40, CSNK1G1, BRSK1, CAMK2D, CAB39, CAMK2A, STK39, PRKX, ACVR1B, NLK, KALRN, EGFR, STK3, TTBK2, CAMKK2, PPP4C, MAPK7, IRAK1, PRKY, MKNK2, GRK6, PAK6, MARK4, MAP2K7, PAK2, MARK1, SRPK2, RIOK3, NEK6, LIMK2, PLK2, LIMK1, CDC42BPB, GTF2H2, HIPK1, MAPK14, ACVR2B, ACVR2A, WEE1, CDK6, RPS6KB1, WNK1, TAOK1, FOSB, PKN2, MAP3K14, MAP3K12, CDK5R1, BMPR1A

Table S5: Most significant Gene Ontology terms (Molecular Function) associated with miRNA targeted genes for PRAD.

Molecular Function	Count	Genes
GO:0000976 transcription regulatory region sequence-specific DNA binding	110	EHF, SPI1, NR3C1, BACH1, BACH2, HOXA10, SOX1, RUVBL2, MYB, DPF2, SOX9, PITX1, JUNB, SOX4, MEF2A, SMARCC1, MEF2C, SOX11, MITF, TBR1, SOX12, FOXP4, ISL1, MYT1, FOXP2, AR, HOXB3, PGR, TP53, CLOCK, ATF3, HOXB5, GATA6, GATA3, GATA2, NEUROD1, NEUROD6, OTX2, ZNF148, TFAP2A, SMAD1, TFAP2B, BCL11B, BCL11A, H3F3A, ESRRG, NR1D2, SMAD5, POU2F3, NR2F6, SMARCA4, NR4A2, BCL6, SP1, SP3, SNAI1, HNRNPC, MXD1, NFE2L2, SUV39H2, RARG, ONECUT2, CHD4, CTCF, MEOX2, HOXC11, MYBL2, MYBL1, GABPA, NKX3-1, KLF10, KLF12, FOXD1, H2AFZ, ETV1, FOS, ELF1, CREB1, NR5A2, ELF4, PAX9, CRY2, RARA, RARB, PPARA, SMARCD2, PLAG1, RELA, MNT, RXRA, MTA1, NFIL3, CUX1, NRIP1, MEF2D, E2F7, FOXJ2, ARID3A, ARID3B, TBX5
GO:0001012 RNA polymerase II regulatory region DNA binding	98	EHF, SPI1, NR3C1, BACH1, BACH2, HOXA10, RUVBL2, MYB, DPF2, PITX1, JUNB, MEF2A, SMARCC1, MEF2C, SOX11, MITF, TBR1, FOXP4, FOXP2, AR, HOXB3, PGR, TP53, CLOCK, ATF3, HOXB5, GATA6, GATA3, GATA2, NEUROD1, NEUROD6, OTX2, TFAP2A, SMAD1, TFAP2B, BCL11B, BCL11A, H3F3A, ESRRG, SMAD5, POU2F3, NR2F6, SMARCA4, NR4A2, BCL6, SP1, SP3, SNAI1, HNRNPC, MXD1, NFE2L2, RARG, ONECUT2, CHD4, CTCF, MEOX2, HOXC11, MYBL2, MYBL1, GABPA, NCOA1, KLF12, FOXD1, H2AFZ, ETV1, FOS, NCOR2, ELF1, CREB1, NR5A2, ELF4, PAX9, RARA, RARB, PPARG, PPARA, SMARCD2, PLAG1, RELA, MNT, RXRA, NFIL3, CUX1, MEF2D, E2F7, FOXJ2, ARID3A, ARID3B, TBX5, MEIS2, GATAD2B, POU4F2, CTCFL, MEIS1, NR6A1, NFIA, NFIB, GZF1
GO:0000981 sequence-specific DNA binding RNA polymerase II transcription factor activity	122	EHF, SPI1, GFI1, RORC, NR3C1, BACH1, ETS1, BACH2, HOXA10, DACH1, MYB, SOX9, SOX6, PITX1, SOX4, TLE4, MEF2A, MEF2C, MITF, SOX11, SOX12, FOXP4, FOXP2, AR, PGR, ERG, TP53, CLOCK, ATF3, HOXB5, ZSCAN29, GATA6, GATA3, PHB, GATA2, HIF1A, FOXO1, NEUROD1, SCRT2, NEUROD6, ZKSCAN2, OTX2, TFAP2A, TFAP2B, ZFHX3, BCL11B, BCL11A, HMGA2, NR1D2, ESRRG, NR2F2, POU2F3, NR2F6, NR4A2, NR4A3, BCL6, SP1, SP3, SNAI1, SNAI2, MXD1, RCOR1, BMPR1A, NFE2L2, RARG, BTG2, ZNF131, THRB, ONECUT2, FOXE1, HNF4G, CTCF, MEOX2, HOXC11, MYBL2, MYBL1, GABPA, NKX3-1, KLF11, KLF12, FOXD1, PAX3, ETV1, ELF1, NR5A2, CREB1, ELF2, ELF4, PAX9, RARA, RARB, PPARG, PPARA, CREB5, CEBPB, NFIX, PLAG1, PTGER3, RELA, MNT
GO:0000977 RNA polymerase II regulatory region sequence-specific DNA binding	95	EHF, RARG, SPI1, ONECUT2, CHD4, CTCF, MEOX2, NR3C1, BACH1, HOXC11, BACH2, HOXA10, RUVBL2, MYB, DPF2, MYBL2, JUNB, PITX1, MYBL1, GABPA, MEF2A, SMARCC1, KLF12, MEF2C, FOXD1, H2AFZ, SOX11, MITF, TBR1, ETV1, FOS, FOXP4, FOXP2, AR, ELF1, CREB1, NR5A2, ELF4, PAX9, RARA, HOXB3, RARB, PGR, PPARA, TP53, CLOCK, ATF3, HOXB5, SMARCD2, PLAG1, GATA6, GATA3, GATA2, RELA, NEUROD1, MNT, RXRA, NFIL3, CUX1, NEUROD6, OTX2, MEF2D, E2F7, TFAP2A, SMAD1, TFAP2B, BCL11B, BCL11A, H3F3A, FOXJ2, ESRRG, ARID3A, ARID3B, TBX5, MEIS2, SMAD5, POU2F3, GATAD2B, POU4F2, SMARCA4, NR2F6, CTCFL, NR4A2, MEIS1, NR6A1, BCL6, NFIA, SP1, NFIB, SP3, SNAI1, HNRNPC, MXD1, NFE2L2, GZF1
GO:0000982 RNA polymerase II core promoter proximal region sequence-specific DNA binding transcription factor activity	75	EHF, BTG2, SPI1, ONECUT2, GFI1, CTCF, MEOX2, NR3C1, BACH1, HOXC11, HOXA10, DACH1, MYB, MYBL2, SOX9, PITX1, MYBL1, GABPA, SOX4, MEF2A, MEF2C, FOXD1, MITF, SOX11, ETV1, SOX12, FOXP4, FOXP2, AR, ELF1, CREB1, NR5A2, ELF4, PGR, PPARA, CLOCK, TP53, ATF3, CREB5, CEBPB, PLAG1, GATA3, GATA2, HIF1A, RELA, FOXO1, NEUROD1, SCRT2, NFIL3, NEUROD6, PLAGL2, OTX2, TFAP2A, TFAP2B, BCL11B, BCL11A, FOXJ2, HMGA2, ARID3B, MEIS3, MEIS2, POU2F3, POU4F2, CTCFL, MEIS1, NR6A1, NFIA, SP1, NFIB, SP3, SNAI1, SNAI2, MXD1, RCOR1, GZF1

Table S5: Most significant Gene Ontology terms (Molecular Function) associated with miRNA targeted genes for SKCM.

Molecular Function	Count	Genes
GO:0000982 RNA polymerase II core promoter proximal region sequence-specific DNA binding transcription factor activity	44	CEBPA, BTG2, ONECUT2, PLAG1, ZNF292, LEF1, TCF21, CTCF, NRF1, RBPJ, NR3C1, HIF1A, BACH1, ETS2, YY1, NEUROD1, SOX2, DACH1, NACC2, MYB, OTX1, SOX9, SOX4, MEF2C, BCL11B, BCL11A, MITF, SOX11, KLF4, MEIS2, ESR1, POU4F1, FOXP2, PAX2, POU4F2, ELF1, NR5A2, NFIA, SP1, NFIB, MTF1, SNAI1, MXD1, CREB5
GO:0000976 transcription regulatory region sequence-specific DNA binding	57	ONECUT2, CHD4, CTCF, RBPJ, CHD2, NR3C1, BACH1, ETS2, BACH2, SOX2, SIN3A, MYB, SOX9, JUNB, SOX4, KLF12, MEF2C, LMO4, SOX11, MITF, PAX2, FOXP2, SFPQ, ELF1, NR5A2, PAX8, MTF1, CGGBP1, CEBPA, PLAG1, LEF1, NRF1, NEUROD1, MNT, RBBP4, NACC2, NRIP1, OTX1, ZNF148, SMARCE1, STAT5B, FOXF2, BCL11B, BCL11A, ESRRG, KLF4, MEIS2, ESR1, POU4F1, POU4F2, BCL6, NFIA, SP1, NFIB, SNAI1, HNRNPC, MXD1
GO:0000981 sequence-specific DNA binding RNA polymerase II transcription factor activity	61	BTG2, ZNF131, ONECUT2, ZNF292, TCF21, RORB, CTCF, RBPJ, NR3C1, BACH1, ETS1, BACH2, ETS2, YY1, SOX2, DACH1, MYB, SOX9, SOX4, KLF12, MEF2C, PAX3, MITF, SOX11, PAX2, FOXP2, FOXP1, ELF1, NR5A2, MTF1, CGGBP1, CREB5, CEBPA, NFIX, PLAG1, LEF1, NRF1, HIF1A, NEUROD1, PURA, MNT, NACC2, OTX1, ZFHX3, XBP1, FOXF2, BCL11B, BCL11A, STAT3, ESRRG, KLF4, MEIS2, ESR1, POU4F1, POU4F2, BCL6, NFIA, SP1, NFIB, SNAI1, MXD1
GO:0001077 RNA polymerase II core promoter proximal region sequence-specific DNA binding transcription factor activity involved in positive regulation of transcription	33	CEBPA, BTG2, ONECUT2, PLAG1, ZNF292, LEF1, TCF21, NRF1, RBPJ, NR3C1, HIF1A, NEUROD1, SOX2, MYB, OTX1, SOX9, SOX4, MEF2C, BCL11B, MITF, SOX11, KLF4, ESR1, MEIS2, POU4F1, POU4F2, ELF1, NR5A2, NFIA, SP1, NFIB, MTF1, CREB5
GO:0000977 RNA polymerase II regulatory region sequence-specific DNA binding	47	CEBPA, ONECUT2, PLAG1, LEF1, CHD4, CTCF, NRF1, RBPJ, NR3C1, BACH1, ETS2, BACH2, NEUROD1, MNT, RBBP4, NACC2, MYB, OTX1, JUNB, SMARCE1, STAT5B, KLF12, MEF2C, FOXF2, BCL11B, BCL11A, SOX11, MITF, ESRRG, MEIS2, ESR1, POU4F1, PAX2, FOXP2, POU4F2, ELF1, NR5A2, PAX8, BCL6, NFIA, SP1, NFIB, MTF1, SNAI1, HNRNPC, MXD1, CGGBP1

Table S5: Most significant Gene Ontology terms (Molecular Function) associated with miRNA targeted genes for STAD.

Molecular Function	Count	Genes
GO:0004674 protein serine/threonine kinase activity	123	TRIO, ZAK, STK19, MYLK, RPS6KA4, RPS6KA3, STK11, CCND3, RPS6KA5, RPS6KA2, CHEK1, RPS6KA1, AKT3, PDK4, PIM1, MAP3K8, PIM3, MAP3K9, PRKACA, MAP3K7, PRKACB, MAP3K4, PSKH1, MAP3K5, MAP2K4, PRKAB2, MAP2K1, DYRK1A, DYRK1B, CSNK1D, BCR, MAK, ULK1, PRKD1, RAF1, SQSTM1, CSNK1G1, NLK, PKMYT1, KALRN, NUAK1, STK33, STK38, MARK4, TRPM6, MARK1, SRPK2, NEK9, HMGA2, PHKB, DCLK1, SRPK1, ICK, CDK8, CDK6, WNK3, MAP3K14, MAP3K11, MAP3K12, BMPR1A, SMG1, DYRK2, BMPR2, LRRK2, LRRK1, AATK, IKBKB, AKAP13, FASTK, PBK, TLK1, PRKG1, CHUK, MINK1, HUNK, OXSR1, MASTL, TGFBR1, TGFBR2, CIT, TGFBR3, LATS2, PIK3CA, PDIK1L, SGK1, CAMK2D, PRKAA1, CAB39, ROCK2, ACVR1B, TTN, MAPK9, MKNK1, MKNK2, PAK7, MAPK1, MAPK6, CAMK2G, PAK2, MAPK4
GO:0046332 SMAD binding	34	USP15, TGFB1I1, ACVR1B, PURB, PURA, PMEPA1, RNF111, SMAD2, SMAD1, JUN, SMAD3, RANBP3, SMURF2, SMURF1, USP9X, MAGI2, TCF12, HMGA2, AXIN2, FOS, SMAD6, TGFBR1, TGFBR2, SMAD7, HIPK2, SKI, TGFBR3, FKBP1A, PPM1A, DAB2, ZEB2, BMP2, TRIM33, BMPR1A
GO:0000981 sequence-specific DNA binding RNA polymerase II transcription factor activity	107	FOXA1, RORC, NR3C1, BACH1, ELK1, BACH2, ELK3, HOXA10, DACH1, MYB, PKNOX1, SOX6, ZNF202, SOX4, SOX5, TLE4, MED1, MEF2C, RFX3, SOX11, SOX12, RUNX3, CLOCK, EPAS1, GLIS3, HIF1A, FOXO1, NEUROD1, NEUROD2, SCRT2, NHLH1, ZKSCAN2, OTX1, ESRRA, TFAP2A, TFAP2B, JUN, ZFHX3, TFAP2D, BCL11B, FOXF1, BCL11A, NFATC3, HMGA2, ESRRG, ESR1, NR4A2, NR4A3, BCL6, FUBP1, TCF3, MKX, MXD1, CC2D1A, CC2D1B, MYF5, BMPR1A, RARG, BTG2, ZNF131, THRB, THRA, ONECUT2, HNF4G, MEOX2, HOXC11, HEY2, MYBL1, GABPA, NKX3-2, NKX3-1, KLF11, KLF12, PAX4, ARNT, ETV1, PAX2, CREB1, AEBP2, TAL1, MTF1, RARB, PPARA, CREB5, PPARD, NFIX, PLAG1, PTH, ZNF518B, PTGER3, PURA, MNT, PLAGL1, PLAGL2, MEF2D, E2F7, STAT3, FOXJ2, MEIS2, USF1
GO:0005057 receptor signaling protein activity	48	ARF1, BMPR2, ZAK, ITPR1, CD3E, ACVR1B, NLK, RAPGEFL1, MAPK9, ERBB4, KDR, MAPK1, MAP3K8, MAP3K9, MAPK6, MAP3K7, MAP3K4, MAPK4, MAP3K5, MAP3K2, SMAD2, MAP3K3, SMAD1, MAP2K1, MAP3K1, SMAD3, INSR, OXSR1, SMAD6, MAPK14, SMAD5, ACVR2B, DCLK1, ACVR2A, TGFBR1, SMAD7, TGFBR2, TIAM1, ADCYAP1, TBL3, DOK4, TYRO3, RAF1, MAP3K14, MAP3K11, NSMAF, BMPR1A, MAP3K12
GO:0019901 protein kinase binding	109	CCNT2, IRS1, DIXDC1, IRS2, RPS6KA4, RPS6KA3, LIPE, CCND3, CCND2, CCND1, DPYSL2, PRKACA, MAP3K5, MAP2K4, MAP2K1, CASR, MEF2C, ANK2, CDC25A, CDC25B, EEF1A1, CCNE2, MSH2, CCNE1, ADAM9, ULK1, UTRN, RAF1, EPHA1, SQSTM1, PFKFB2, STXBP1, IQGAP1, HIF1A, ZBTB4, BAG5, RACGAP1, PRKAR2A, STK38, DVL1, RAB11FIP2, BCL2L14, NEK9, SMAD1, SMAD3, STK11IP, MSN, CYLD, PTPRC, NBEA, PKIA, GRB2, TCF3, PAM, MAP3K11, MAP3K12, USP37, PTPRR, CLTC, PDE3B, PTEN, AP2A1, CD3E, PKD1, GHR, IKBKB, SPRED1, RB1CC1, ITGAV, NEFH, DUSP2, AXIN2, YWHAZ, PJA2, ADRA2A, TGFBR2, KAT2B, CRY2, DCX, RAPGEF2, ARHGEF7, HDAC4, STX17, TTN, CDC42, MAPK9, RNF138, CAMK2N1, CAMK2N2, MAPK1, RICTOR, PDLIM5, MAPK6, PAK2, SASH1, MAPK4, MAP3K2, PTPN1, MAP3K1, SLC12A5

Table S5: Most significant Gene Ontology terms (Molecular Function) associated with miRNA targeted genes for THCA.
