# Supplementary material for: Genome-wide analysis of NGS data to compile cancer-specific panels of miRNA biomarkers
Source: PLoS One. 2018 Jul 26;13(7):e0200353. doi: 10.1371/journal.pone.0200353 (PMC6061989; doi:10.1371/journal.pone.0200353)
Supplement: S1 Appendix — Detailed results of the experiments. (PDF) [file pone.0200353.s006.pdf]

S1 Appendix for
"Genome-wide analysis of NGS data to compile cancer-specific panels of miRNA biomarkers"

1 Extended Results

1.1 Statistical Significance Test

Statistical significance of DE+KPCA+SVM or DE+PCA+SVM with respect to the other methods is analyzed by using non-parametric test like Friedman test, at 5% significance level. According to Friedman test, it is assumed that for a null hypothesis there is no significant difference between the accuracy values of different methods. Whereas, according to the alternative hypothesis, it is considered that there is a significant difference in the accuracy values within the used methods. The Friedman statistic (Chi-Square value) is computed as follows, where the number of datasets and methods are represented by U and Q, respectively.

$$H_F = \frac{12}{UQ(Q+1)} \sum_{j=1}^{Q} R_j^2 - 3U(Q+1) \tag{1}$$

Square sum of the ranks, scored by different methods are given by $\sum_{j=1}^{Q} R_j^2$. Table A1 reports the ranks computed by Friedman test corresponding to all the methods for 10 different miRNA datasets. In this regard, average rank of DE+KPCA+SVM or DE+PCA+SVM is found to be promising among other methods by achieving minimum average rank as 2 and 2.3, respectively. Moreover, the corresponding Chi-Square value H_F and p-value are 108.8857 and 0.00001 respectively at 5% significance level, which also emphasize the acceptance of the alternative hypothesis. Therefore, the results produced by the proposed method are statistically significant for miRNA data classification.

Table A1: Friedman ranks of all the methods for different miRNA datasets

Tissue	Our Method	DE+ KPCA	DE+ PCA	KPCA	PCA	KECA	ICA	RFFS	MIFS	t-test	mRMR	RankSum	JMI	SNR
BRCA	1	3	2	5	4	14	13	6	8	7	11.5	9.5	11.5	9.5
KIRC	1	2	3	6	7	9	8	10	4.5	12	4.5	13.5	11	13.5
LGG	1	2	3	4	5	6	8	7	9	12	10	13.5	11	13.5
LIHC	2	3	1	5	4	6	8	7	10	9	14	12	12	12
LUAD	1	2	3	4	5	6	8	7	9	10	14	12.5	11	12.5
PAAD	1	2	3	4	5	7	6	8	14	9	12	10.5	13	10.5
PRAD	3	1	2	4	5	6	8	7	14	9.5	11	9.5	12.5	12.5
SKCM	1	2	3	4	5	6	8	7	9	12.5	11	10	14	12.5
STAD	5	2	1	4	3	6	8	7	9	12	14	10	12	12
THCA	3	1	2	4	5	6	8	7	10	9	11.5	13.5	11.5	13.5
**Average Rank**	**1.9**	**2**	**2.3**	**4.4**	**4.8**	**7.2**	**8.3**	**7.3**	**9.65**	**10.2**	**11.35**	**11.45**	**11.95**	**12.2**

Table A2: **Average values of Classification performance metrics for different feature selection methods**

	Algorithm	BRCA	KIRC	LGG	LIHC	LUAD	PAAD	PRAD	SKCM	STAD	THCA	Average
Accuracy	Our Method	0.969	0.973	0.990	0.948	0.962	0.964	0.943	0.970	0.924	0.933	0.958
	DE+KPCA	0.945	0.960	0.961	0.941	0.961	0.962	0.960	0.962	0.947	0.959	0.956
	DE+PCA	0.952	0.937	0.947	0.961	0.943	0.937	0.948	0.956	0.960	0.947	0.949
	KPCA	0.917	0.928	0.929	0.928	0.929	0.930	0.929	0.930	0.927	0.928	0.928
	PCA	0.921	0.923	0.926	0.929	0.928	0.926	0.926	0.929	0.928	0.927	0.926
	KECA	0.516	0.906	0.922	0.914	0.918	0.916	0.920	0.918	0.914	0.923	0.877
	ICA	0.618	0.911	0.841	0.870	0.840	0.919	0.839	0.844	0.860	0.840	0.838
	RFFS	0.901	0.897	0.914	0.891	0.902	0.879	0.882	0.908	0.884	0.899	0.896
	MIFS	0.767	0.932	0.818	0.811	0.815	0.788	0.803	0.834	0.808	0.813	0.819
	t-test	0.768	0.808	0.815	0.813	0.810	0.814	0.809	0.817	0.806	0.814	0.807
	mRMR	0.757	0.932	0.817	0.807	0.805	0.811	0.809	0.821	0.804	0.810	0.817
	RankSum	0.764	0.803	0.811	0.808	0.806	0.812	0.809	0.823	0.808	0.808	0.805
	JMI	0.757	0.842	0.817	0.808	0.808	0.809	0.805	0.816	0.806	0.810	0.808
	SNR	0.764	0.803	0.811	0.808	0.806	0.812	0.805	0.817	0.806	0.808	0.804
F-Measure	Our Method	0.951	0.964	0.972	0.944	0.954	0.952	0.941	0.961	0.893	0.935	0.947
	DE+KPCA	0.940	0.950	0.958	0.938	0.959	0.955	0.959	0.960	0.944	0.958	0.952
	DE+PCA	0.950	0.934	0.932	0.959	0.934	0.938	0.937	0.942	0.959	0.940	0.942
	KPCA	0.940	0.941	0.947	0.938	0.945	0.951	0.941	0.951	0.942	0.942	0.944
	PCA	0.940	0.930	0.947	0.946	0.946	0.947	0.946	0.944	0.946	0.941	0.943
	KECA	0.656	0.947	0.923	0.952	0.962	0.931	0.962	0.976	0.970	0.928	0.921
	ICA	0.709	0.949	0.893	0.922	0.932	0.933	0.933	0.937	0.916	0.889	0.901
	RFFS	0.905	0.891	0.914	0.889	0.903	0.854	0.884	0.908	0.883	0.900	0.893
	MIFS	0.747	0.974	0.877	0.865	0.882	0.804	0.854	0.927	0.864	0.864	0.866
	t-test	0.748	0.864	0.871	0.871	0.891	0.829	0.871	0.917	0.862	0.864	0.859
	mRMR	0.743	0.974	0.875	0.858	0.887	0.820	0.879	0.922	0.856	0.860	0.867
	RankSum	0.744	0.859	0.867	0.867	0.887	0.827	0.871	0.923	0.863	0.858	0.856
	JMI	0.743	0.824	0.874	0.867	0.889	0.816	0.867	0.907	0.862	0.860	0.851
	SNR	0.744	0.859	0.867	0.867	0.887	0.827	0.867	0.917	0.862	0.858	0.855
MCC	Our Method	0.910	0.911	0.924	0.902	0.910	0.911	0.903	0.917	0.897	0.890	0.907
	DE+KPCA	0.904	0.900	0.910	0.900	0.910	0.900	0.910	0.910	0.905	0.910	0.906
	DE+PCA	0.890	0.896	0.905	0.910	0.900	0.895	0.902	0.914	0.910	0.905	0.903
	KPCA	0.910	0.904	0.910	0.903	0.907	0.915	0.909	0.914	0.903	0.908	0.909
	PCA	0.907	0.906	0.908	0.913	0.904	0.907	0.905	0.904	0.902	0.908	0.906
	KECA	0.690	0.890	0.900	0.900	0.900	0.900	0.900	0.900	0.900	0.900	0.870
	ICA	0.730	0.900	0.880	0.880	0.880	0.900	0.890	0.890	0.880	0.880	0.870
	RFFS	0.880	0.872	0.908	0.862	0.884	0.826	0.844	0.896	0.848	0.878	0.870
	MIFS	0.810	0.880	0.820	0.830	0.870	0.800	0.850	0.880	0.830	0.840	0.840
	t-test	0.810	0.850	0.820	0.850	0.850	0.840	0.890	0.880	0.820	0.840	0.840
	mRMR	0.750	0.880	0.820	0.820	0.870	0.820	0.850	0.880	0.820	0.830	0.830
	RankSum	0.790	0.820	0.810	0.830	0.840	0.830	0.890	0.900	0.830	0.820	0.830
	JMI	0.750	0.870	0.820	0.830	0.840	0.810	0.830	0.870	0.820	0.830	0.820
	SNR	0.790	0.820	0.810	0.830	0.840	0.830	0.830	0.880	0.820	0.820	0.820
AUC	Our Method	0.922	0.950	0.965	0.900	0.922	0.911	0.900	0.950	0.900	0.899	0.922
	DE+KPCA	0.900	0.911	0.940	0.900	0.931	0.940	0.922	0.950	0.910	0.904	0.921
	DE+PCA	0.911	0.900	0.900	0.910	0.910	0.900	0.900	0.899	0.900	0.900	0.903
	KPCA	0.899	0.900	0.900	0.900	0.900	0.910	0.900	0.910	0.900	0.900	0.902
	PCA	0.900	0.900	0.900	0.900	0.900	0.900	0.900	0.900	0.900	0.900	0.900
	KECA	0.711	0.850	0.900	0.910	0.910	0.900	0.900	0.899	0.900	0.900	0.878
	ICA	0.730	0.899	0.881	0.860	0.866	0.900	0.865	0.870	0.865	0.860	0.859
	RFFS	0.896	0.906	0.910	0.894	0.897	0.887	0.885	0.901	0.877	0.906	0.896
	MIFS	0.778	0.901	0.880	0.839	0.865	0.779	0.783	0.886	0.831	0.861	0.840
	t-test	0.779	0.831	0.865	0.851	0.839	0.865	0.834	0.875	0.830	0.865	0.843
	mRMR	0.769	0.901	0.880	0.831	0.812	0.842	0.834	0.880	0.801	0.834	0.838
	RankSum	0.771	0.790	0.842	0.834	0.821	0.842	0.834	0.886	0.831	0.831	0.828
	JMI	0.769	0.886	0.875	0.834	0.831	0.834	0.812	0.875	0.830	0.834	0.838
	SNR	0.771	0.790	0.842	0.834	0.821	0.842	0.812	0.875	0.830	0.831	0.824
Execution Time	Our Method	54189.274	37008.048	46154.238	30883.225	44502.113	30800.532	48356.480	44565.942	30646.211	44510.915	10963.640
	DE+KPCA	1367.005	739.975	922.935	792.880	889.815	615.840	966.980	891.120	807.590	890.960	888.510
	DE+PCA	1083.570	618.985	754.335	617.815	774.190	516.135	772.640	696.380	612.660	726.155	717.287
	KPCA	13.902	5.476	7.970	6.427	7.916	4.606	8.056	7.230	7.620	7.915	7.712
	PCA	9.296	3.706	5.426	4.648	5.385	3.294	4.839	5.328	6.813	5.095	5.383
	KECA	16.159	5.516	11.859	7.407	10.883	4.692	8.553	8.788	7.628	8.659	9.014
	ICA	14.239	5.305	8.712	6.901	8.548	5.480	8.088	7.970	7.405	8.743	8.139
	RFFS	10.468	6.962	5.895	7.715	8.409	6.703	8.487	7.190	8.300	8.450	7.858
	MIFS	1.343	0.712	0.957	0.900	0.977	0.629	1.103	0.921	0.883	1.011	0.944
	t-test	62.553	61.939	65.332	64.305	67.431	66.021	65.087	60.640	65.720	63.540	64.257
	mRMR	0.802	0.402	0.429	0.455	0.641	0.443	0.652	0.486	0.479	0.460	0.525
	RankSum	1.711	0.756	1.049	0.842	0.999	0.645	0.917	0.902	0.918	0.944	0.968
	JMI	0.920	0.506	0.453	0.559	0.624	0.346	0.608	0.508	0.553	0.519	0.559
	SNR	0.961	0.414	0.789	0.482	0.603	0.311	0.582	0.469	0.428	0.620	0.566

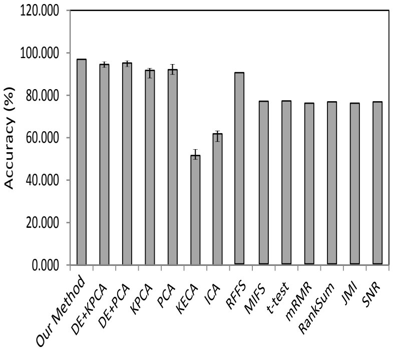

(a) BRCA

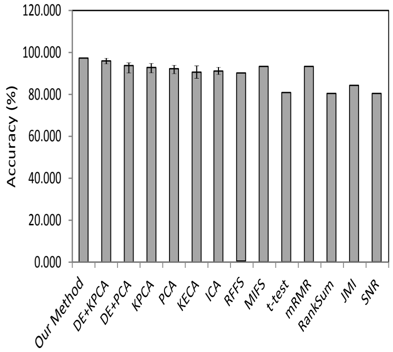

(b) KIRC

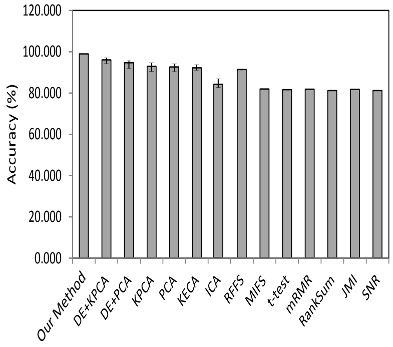

(c) LGG

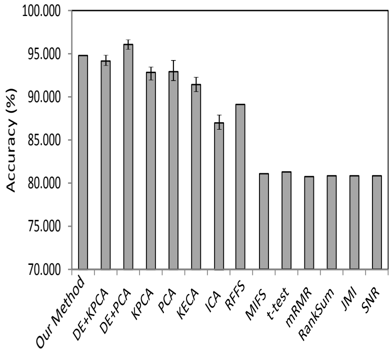

(d) LIHC

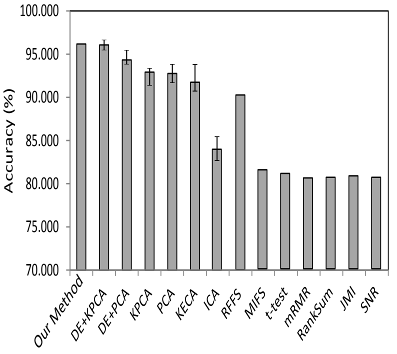

(e) LUAD

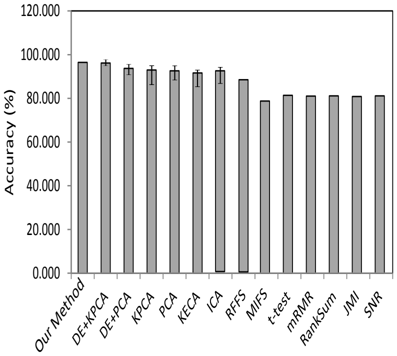

(f) PAAD

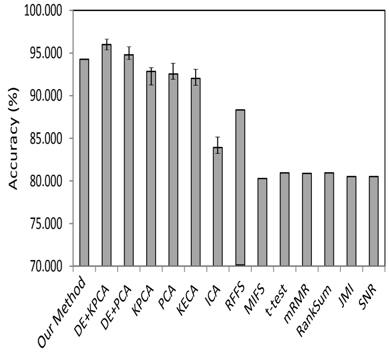

(g) PRAD

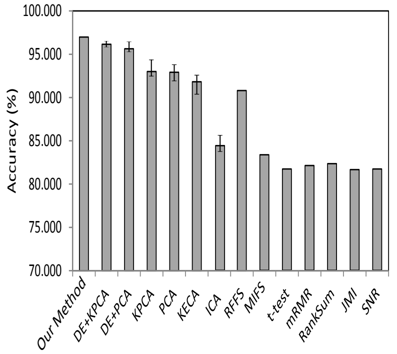

(h) SKCM

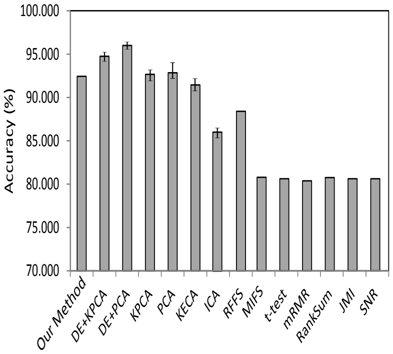

(i) STAD

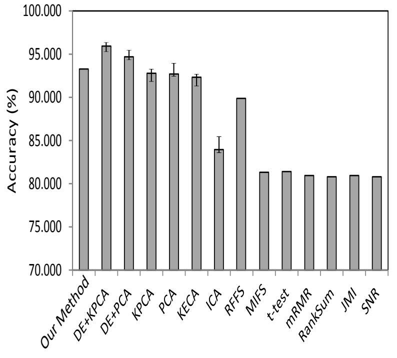

(j) THCA

Figure A1: Comparison of average accuracy value of different feature selection methods

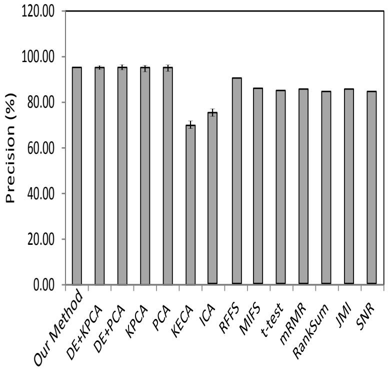

(a) BRCA

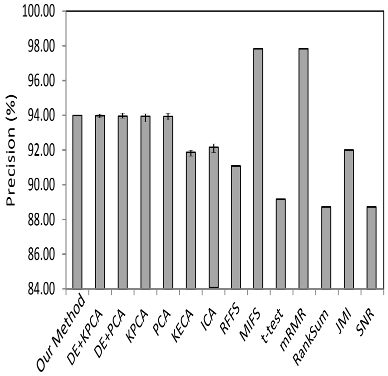

(b) KIRC

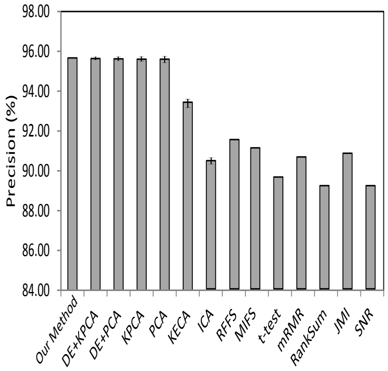

(c) LGG

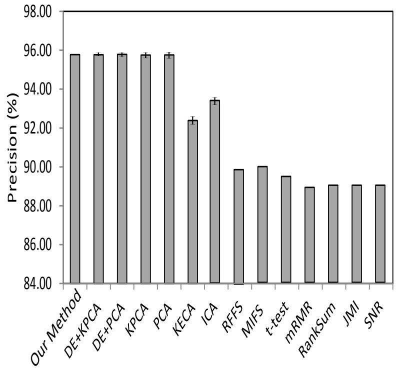

(d) LIHC

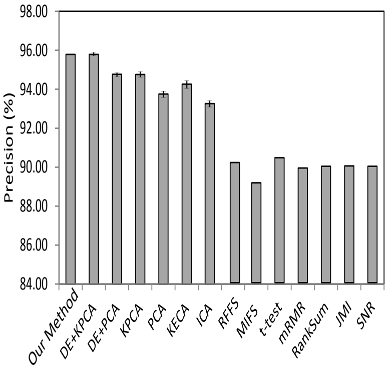

(e) LUAD

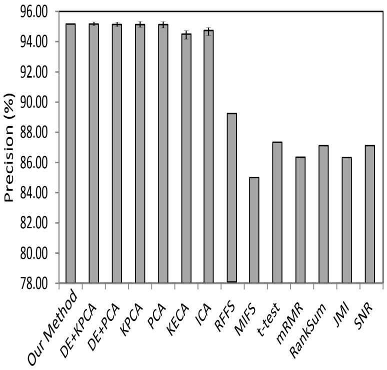

(f) PAAD

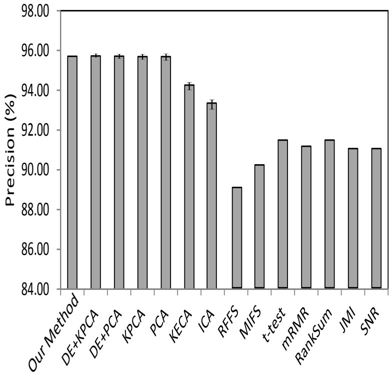

(g) PRAD

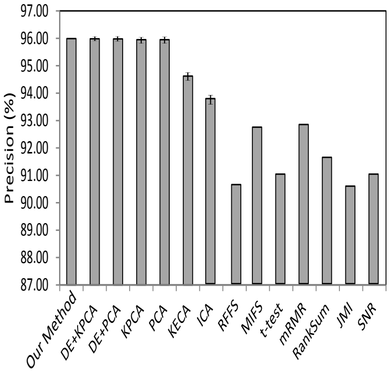

(h) SKCM

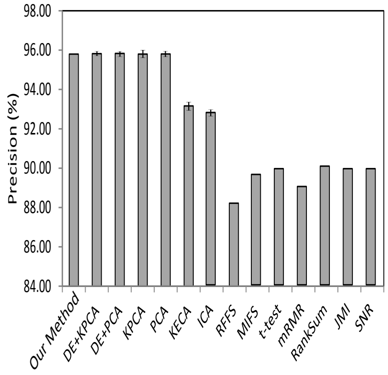

(i) STAD

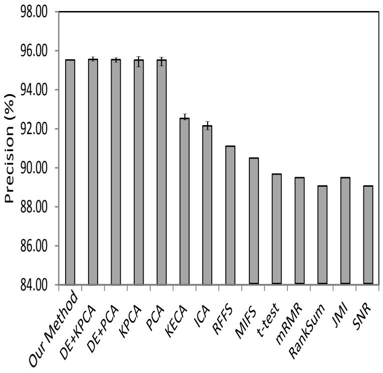

(j) THCA

Figure A2: Comparison of average precision value of different feature selection methods

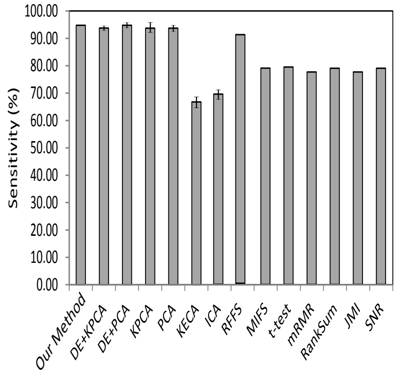

(a) BRCA

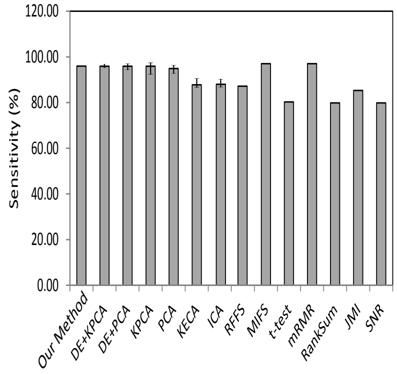

(b) KIRC

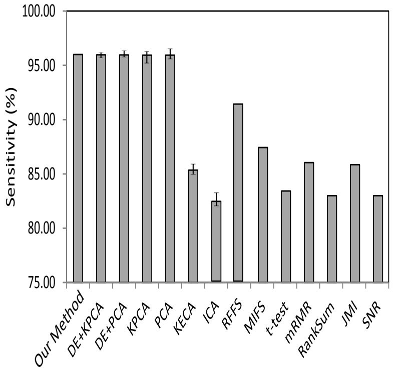

(c) LGG

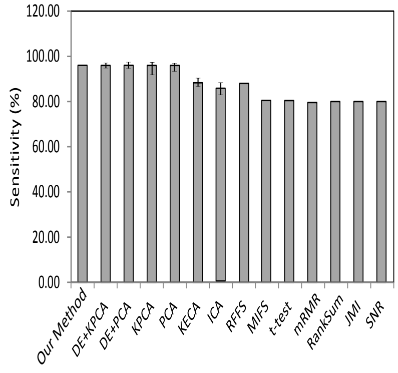

(d) LIHC

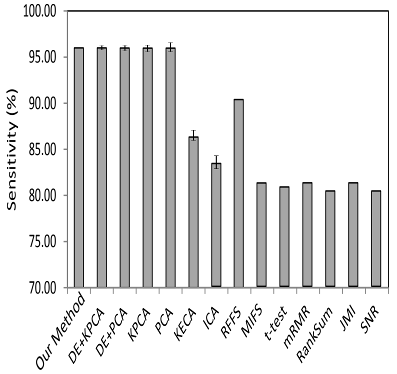

(e) LUAD

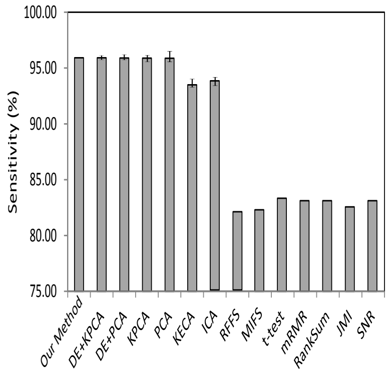

(f) PAAD

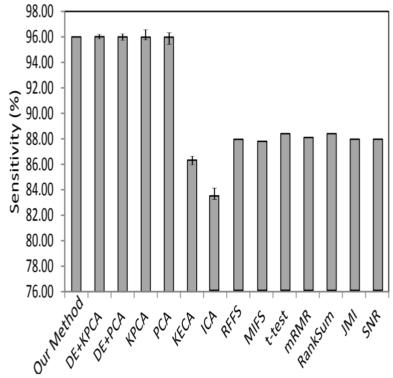

(g) PRAD

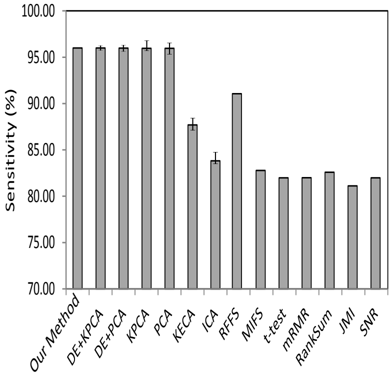

(h) SKCM

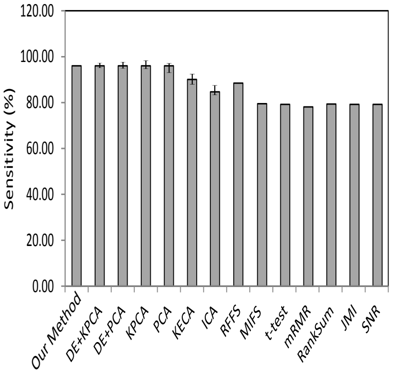

(i) STAD

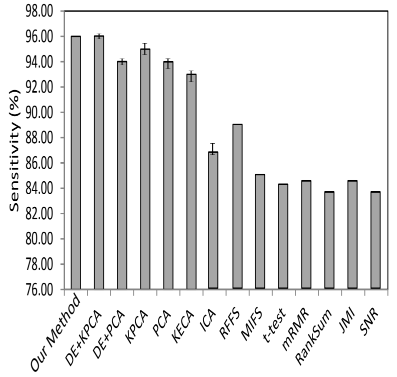

(j) THCA

Figure A3: Comparison of average sensitivity value of different feature selection methods

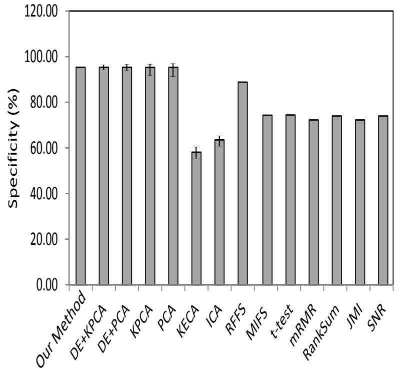

(a) BRCA

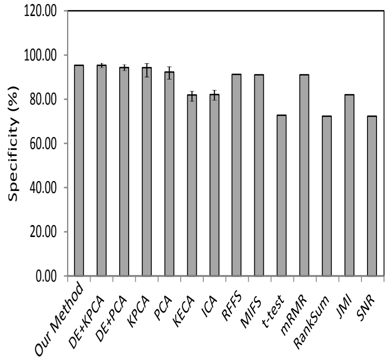

(b) KIRC

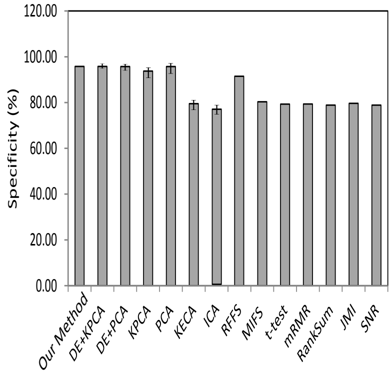

(c) LGG

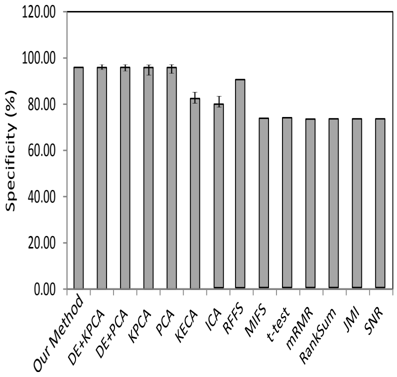

(d) LIHC

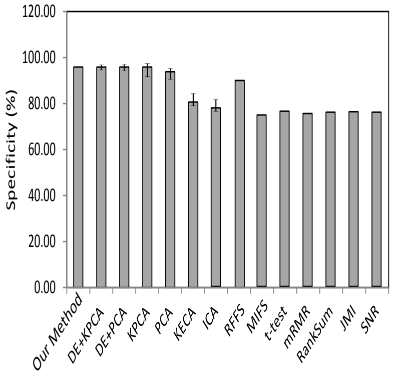

(e) LUAD

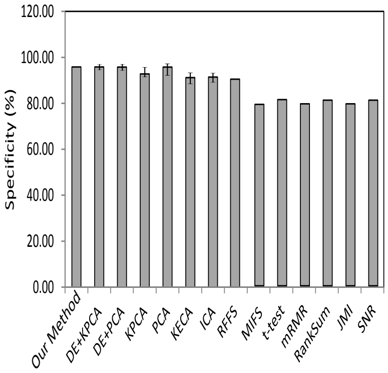

(f) PAAD

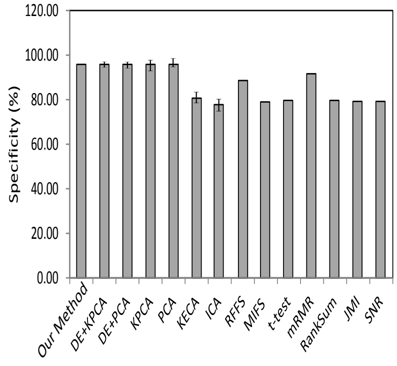

(g) PRAD

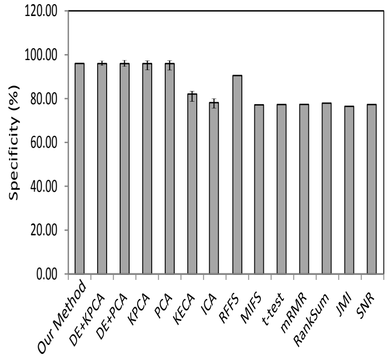

(h) SKCM

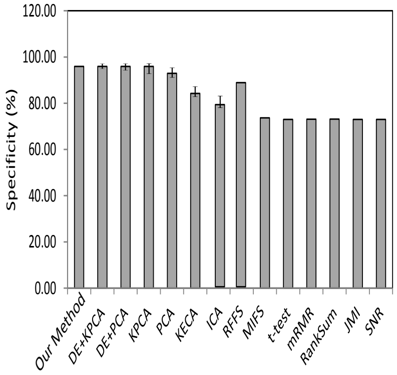

(i) STAD

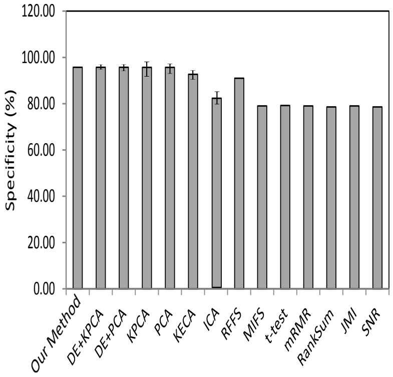

(j) THCA

Figure A4: Comparison of average specificity value of different feature selection methods

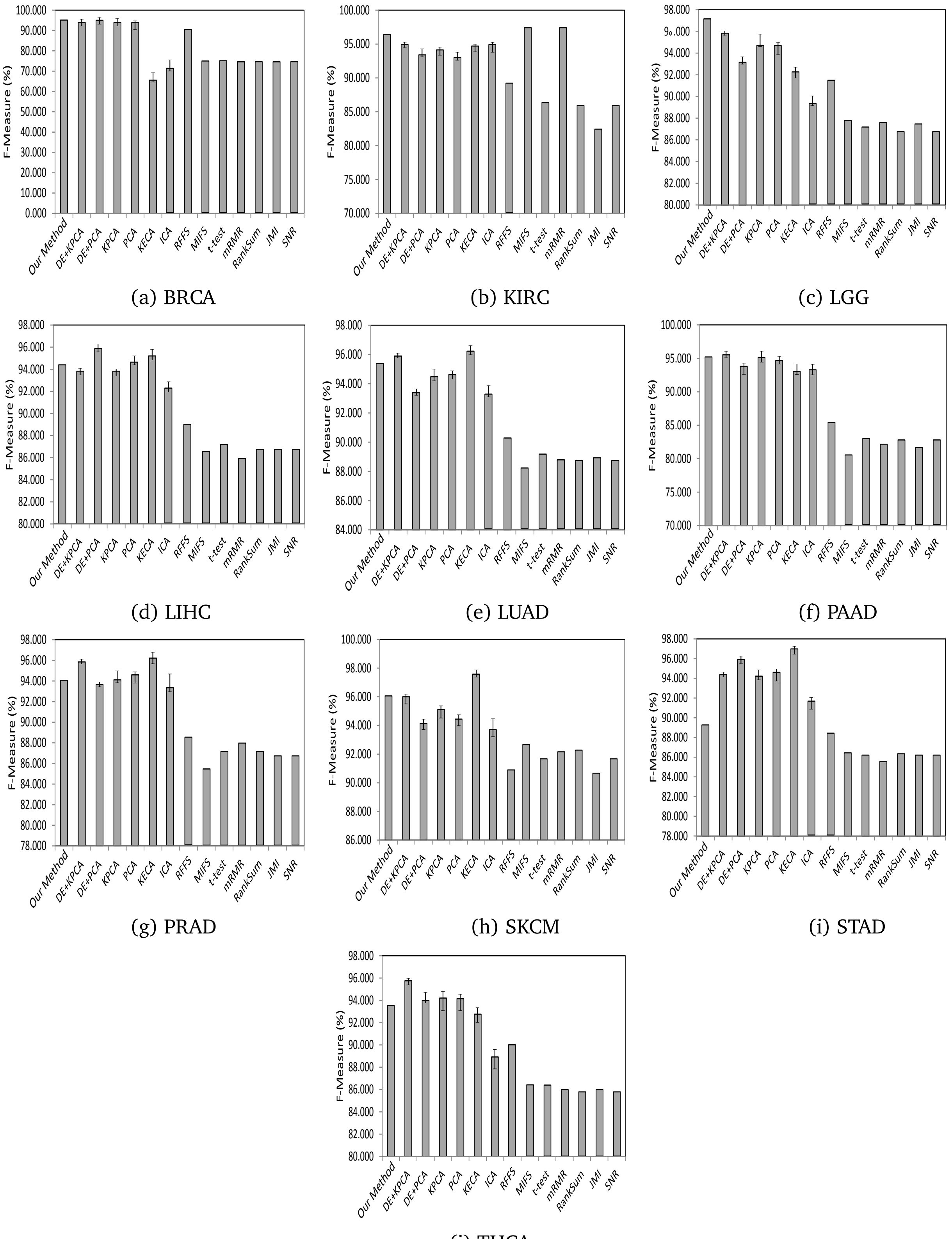

(a) BRCA (b) KIRC (c) LGG

(d) LIHC (e) LUAD (f) PAAD

(g) PRAD (h) SKCM (i) STAD

(j) THCA

Figure A5: Comparison of average F-Measure value of different feature selection methods

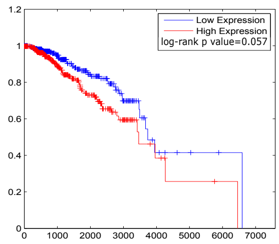

(a) hsa-mir-140

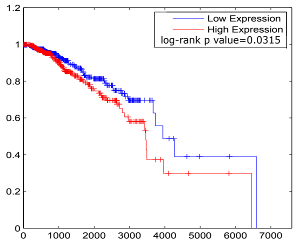

(b) hsa-mir-375

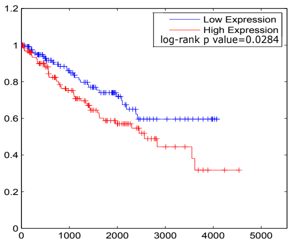

(c) hsa-mir-361

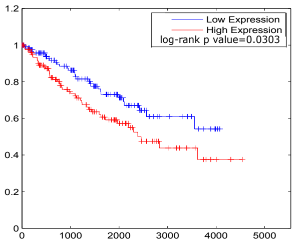

(d) hsa-mir-500a

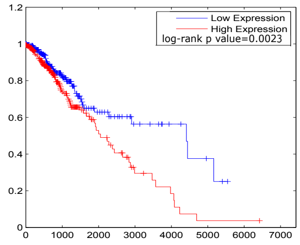

(e) hsa-mir-21

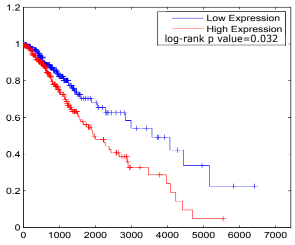

(f) hsa-mir-155

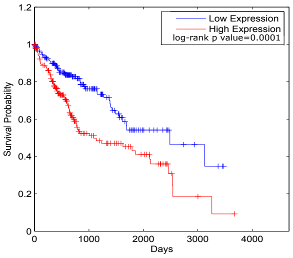

(g) hsa-mir-10b

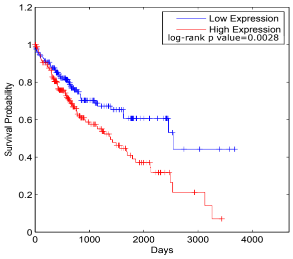

(h) hsa-mir-17

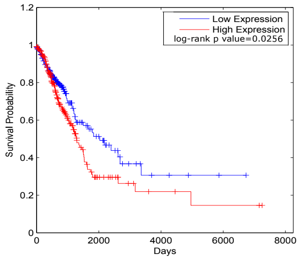

(i) hsa-mir-195

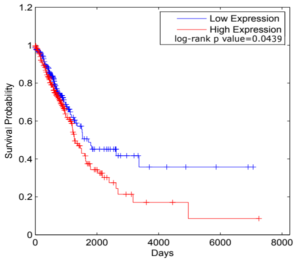

(j) hsa-mir-574

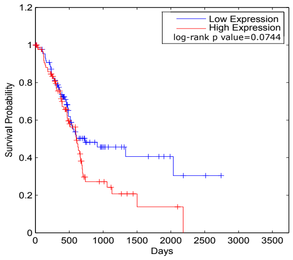

(k) hsa-mir-194-2

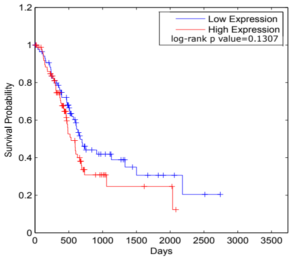

(l) hsa-mir-598

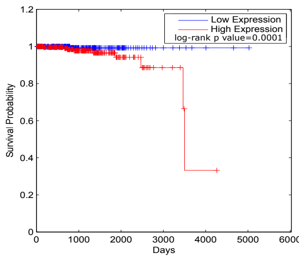

(m) hsa-mir-27b

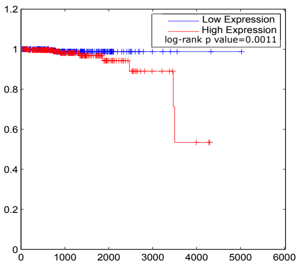

(n) hsa-mir-342

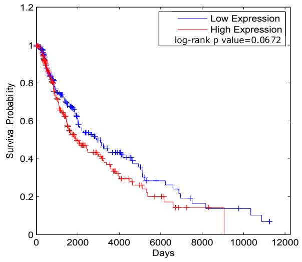

(o) hsa-mir-30a

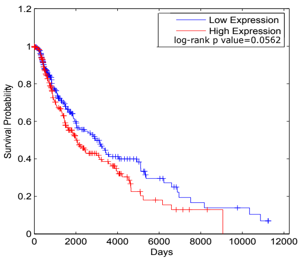

(p) hsa-mir-425

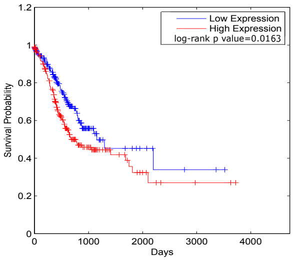

(q) hsa-mir-542

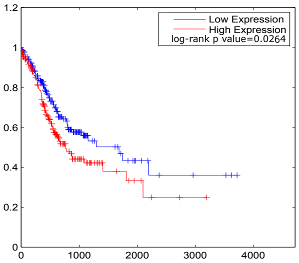

(r) hsa-mir-744

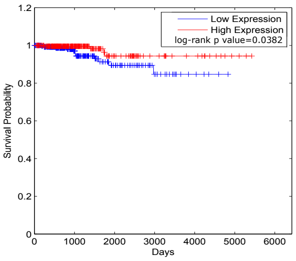

(s) hsa-mir-33a

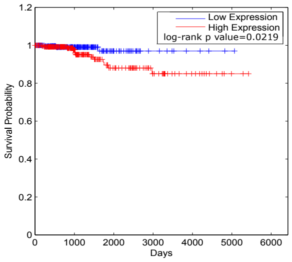

(t) hsa-mir-576

Figure A6: Kaplan-Meier survival plots of 2nd and 3rd best miRNAs, in total *20*, based on log-rank *p*-values to their respective cancer types, (a)-(b) BRCA, (c)-(d) KIRC, (e)-(f) LGG, (g)-(h) LIHC, (i)-(j) LUAD, (k)-(l) PAAD, (m)-(n) PRAD, (o)-(p) SKCM, (q)-(r) STAD and (s)-(t) THCA
